# Synthesis and Antiplasmodial Activity of Novel Fosmidomycin Derivatives and Conjugates with Artemisinin and Aminochloroquinoline

**DOI:** 10.3390/molecules25204858

**Published:** 2020-10-21

**Authors:** Despina Palla, Antonia I. Antoniou, Michel Baltas, Christophe Menendez, Philippe Grellier, Elisabeth Mouray, Constantinos M. Athanassopoulos

**Affiliations:** 1 Synthetic Organic Chemistry Laboratory, Department of Chemistry, University of Patras, GR-26504 Patras, Greece; despina.pal.5@gmail.com (D.P.); tonadoniou@upatras.gr (A.I.A.); 2 LSPCMIB, UMR-CNRS 5068, Université Paul Sabatier-Toulouse III, CEDEX 9, 31062 Toulouse, France or michel.baltas@lcc-toulouse.fr (M.B.); menendez@chimie.ups-tlse.fr (C.M.); 3 CNRS, LCC (Laboratoire de Chimie, de Coordination), Université de Tolouse, UPS, INPT, 205 Route de Narbonne, BP 44099, CEDEX 4 F-31077 Toulouse, France; 4 MCAM, UMR 7245, Muséum National d’Histoire Naturelle, CNRS, CP52, 63 rue Buffon, 75005 Paris, France; grellier@mnhn.fr (P.G.); mouray@mnhn.fr (E.M.)

**Keywords:** fosmidomycin, chloroquine, artemisinin, hybrid, conjugates, antimalarial activity

## Abstract

Malaria, despite many efforts, remains among the most problematic infectious diseases worldwide, mainly due to the development of drug resistance by *Plasmodium falciparum.* The antibiotic fosmidomycin (FSM) is also known for its antimalarial activity by targeting the non-mevalonate isoprenoid synthesis pathway, which is essential for the malaria parasites but is absent in mammalians. In this study, we synthesized and evaluated against the chloroquine-resistant *P. falciparum FcB1/Colombia* strain, a series of FSM analogs, derivatives, and conjugates with other antimalarial agents, such as artemisinin (ART) and aminochloroquinoline (ACQ). The biological evaluation revealed four new compounds with higher antimalarial activity than FSM: two FSM-ACQ derivatives and two FSM-ART conjugates, with 3.5–5.4 and 41.5–23.1 times more potent activities than FSM, respectively.

## 1. Introduction

Despite worldwide efforts, malaria remains among the most dangerous infectious diseases. Malaria incidence has decreased significantly since 2010, but, unfortunately, after 2014, the number of malaria cases is increasing again, according to the World Health Organization [1]. The main reason is the elevated resistance to artemisinin combination therapy (ACT) [2]. In that respect, there is an urgent need for antimalarial agents with a novel mechanism of action (MOA).

**Figure 1:**
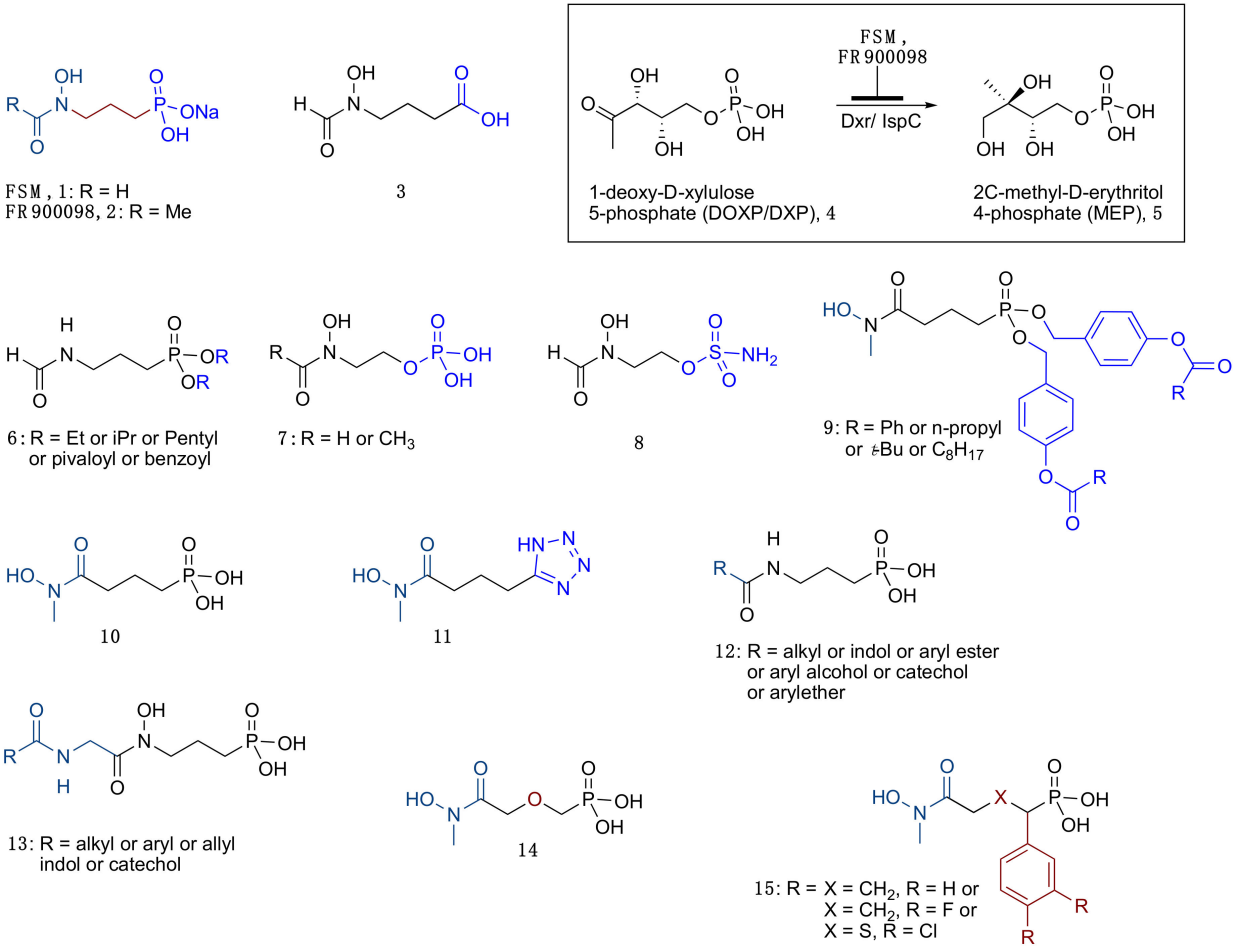
Structures of FSM, FR9000098, and various FSM derivatives.

During the last decade of the 20th century, the discovery of the non-mevalonate isoprenoid biosynthesis pathway as an essential target raised hope for potential therapeutic opportunities [3]. The pathway is proved to be essential for malaria parasites, while it is absent in mammalian hosts who generate isoprenoids exclusively via the mevalonate pathway [4,5]. The enzymes of the non-mevalonate pathway are located in the apicoplast organelle of malaria parasites, which only host small numbers of proteins, thus becoming attractive drug targets [6,7,8]. In this respect, the seminal work of Jomaa et al. [9] set the basis of an intense research work on compounds targeting the non-mevalonate pathway of the *Plasmodium*. These authors reported in 1999 that the antibiotic fosmidomycin (FSM, **1, Figure 1**) inhibits the IspC (see Figure 1), which catalyzes the first committed step of the non-mevalonate pathway and is believed to be rate limiting. FSM, which was initially isolated from *Streptomyces lavendulae* [10,11], is a phosphonate structural analogue of the IspC substrate DOXP **(4)**, which furthermore bears a hydroxamic acid moiety in replacement of the alpha hydroxy ketone of **4** and thus is not subject to hydrolysis by cellular phosphatases [12]. FSM may act as a transition state intermediate, with the hydroxamic acid group chelating the metal ion and the phosphono moiety resembling the phosphate group of **4** [13]. FSM was first developed as an antibacterial agent, but due to its less than ideal pharmacokinetics [10,11,14], it was abandoned and later repositioned as a potential antimalarial, inhibiting a new different target compared to artemisinin (ART). The shortcomings of FSM have prompted attempts by several research groups to improve its activity by performing several chemical modifications, suitable for structure–activity relationship studies [15,16,17,18,19,20,21,22]. One of the most studied FSM’s analogues is FR900098 **(2)**, a phosphonic acid antibiotic, where the formyl group of FSM is replaced by an acetyl one. Although it is structurally very close to FSM, it is about twice as active as FSM in vitro and in a mouse model [9,23,24].

In brief, structural modifications of FSM have focused on four main areas: (i) replacement of the phosphonate motif by (bio)isosteres **(3, 7, 8, 11)**, synthesis of phosphonate prodrugs **(6, 9)** [25]; (ii) replacement or modification of the hydroxamate moiety that chelates the catalytically essential divalent cation, e.g., compounds **10**–**14** [26,27]; (iii) modulation of the aliphatic linker between the anionic anchor group and the chelating head group, e.g., compounds **14**–**15** [28]; and (iv) modification of the aliphatic chain by introduction of (typically aromatic) substituents, e.g., compounds **15** [22]. As an example, FR900098-prodrugs, where an acetyl group replaced the formyl one of the FSM, displayed improved in vitro and enhanced in vivo activity [9]. Furthermore, lipophilic phosphonate prodrug derivatives of FSM have previously been reported to display significantly enhanced antiplasmodial [29] and antimicrobial activities [30]. 

Recent studies have shown that the combination of two antimalarial drugs with different molecular targets in one single entity either as a hybrid or a conjugate proved to be more efficient against diseases, such as malaria and cancer [31]. Hybrid molecules are rather promising since they have less possibilities of developing drug resistance [32].

**Figure 2:**
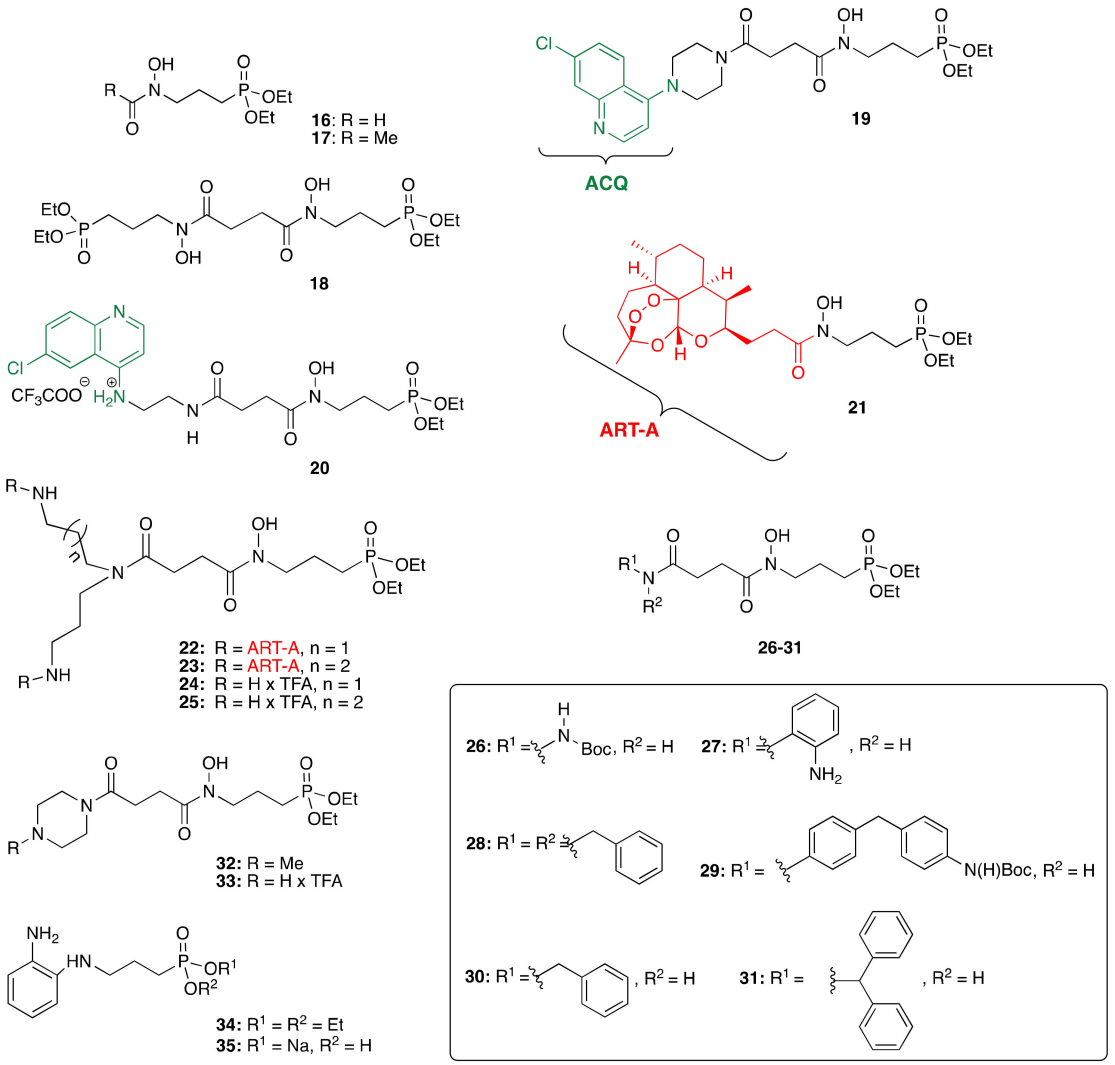
Structures of the FSM derivatives synthesized in this work (**16**–**35)**.

In this work, a synthesis and antiplasmodial evaluation along with the cytotoxicity of three focused families of compounds possessing the propyl phosphonate frame (ester or acid form) of FSM are reported. The first family contains five compounds: FSM, FR900098, and their phosphonate diesters **(16, 17)**, along with a dimeric form of FSM **(18)**. The second family was constructed by attaching active antimalarial pharmacophores like ACQ **(19**–**20)** [33] or ART **(21**–**23)** [34,35] next to the carbonyl group of the hydroxamate thus preparing five new compounds (hybrids or conjugates). The third family (10 compounds, **26**–**33**) possesses various amines connected to the hydroxamic function. For the last two compounds **(34, 35)**, the hydroxamate moiety was replaced by *o*-phenylenediamine, thus changing the nature of the groups that might intervene in the chelation process (Figure 2).

## 2. Results and Discussion

### 2.1. Synthesis of the Key Intermediate **39**, FSM, FR9000098, and Dimer **18**

**Scheme 1:**
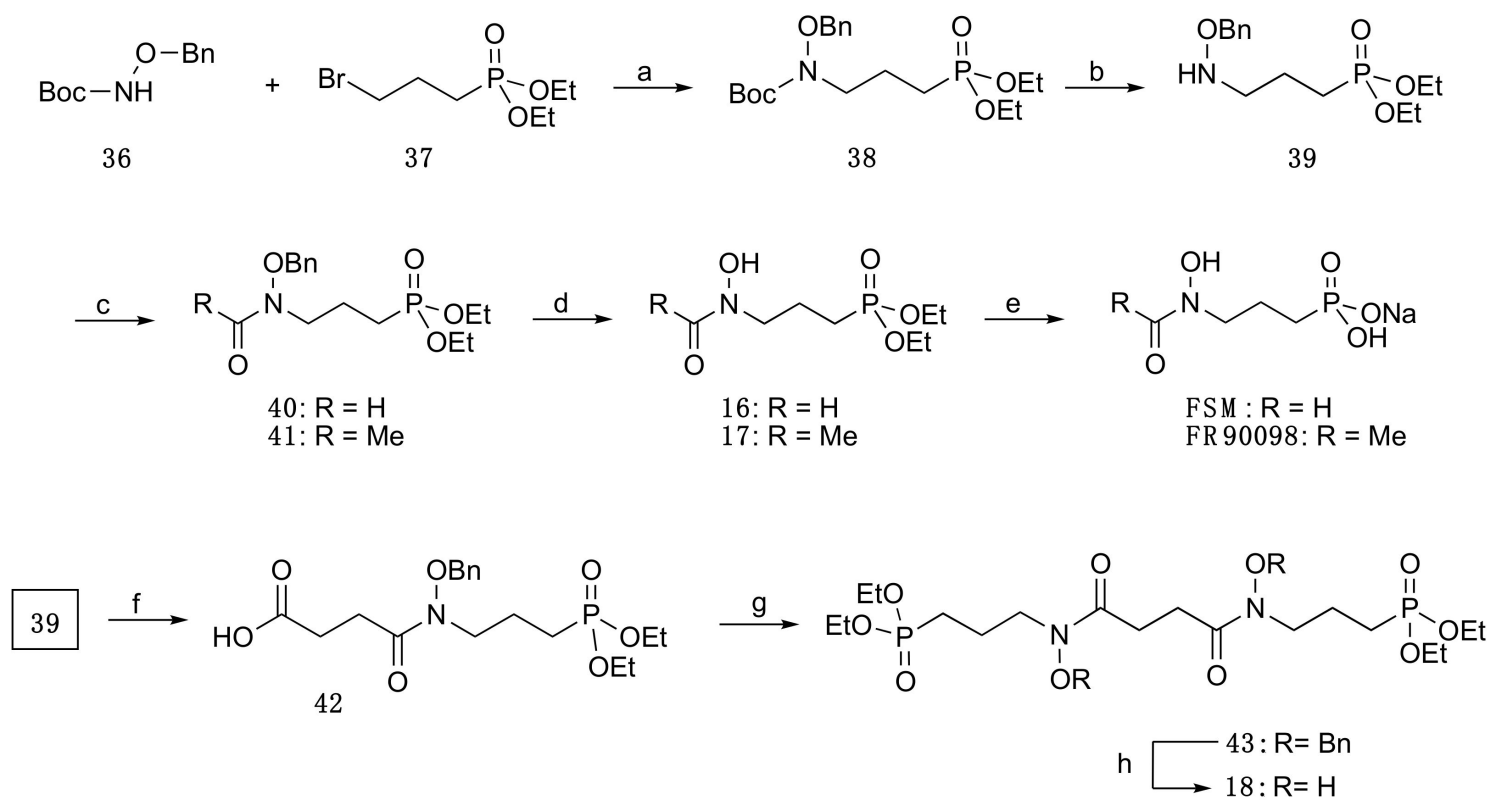
Synthesis of key intermediate **39**, FSM and FR9000098 monosodium salts, and FSM dimer **18**; Reagents and conditions: (a) NaH, NaI, DMF, rt to 60 °C, 17 h, 96%; (b) TFA, CH_2_Cl_2_, 0 °C to rt, 1.5 h, 98%; (c) for **40**: HCOOH, (CH_3_CO)O_2_, −5 °C to rt, overnight, 91% and for **41**: CH_3_COCl, Et_3_N, DCM, 0 °C to rt, overnight, 98%; (d) H_2_, 10% Pd/C, MeOH, rt (3 h, 91% for **16** and 4 h, 99% for **17**); (e) (i) TMSBr, CH_2_Cl_2_, 0 °C to rt, 8 h (ii) 6N aq. NaOH/MeOH (87% for FSM and 90% for FR900098 over 2 steps); (f) succinic anhydride, DIPEA, DMAP, THF, rt, 3 h, 88%; (g) **39**, HBTU, Et_3_N, CHCl_3_, rt, 3.5 h, 61% (h) H_2_, 10% Pd/C, MeOH, rt, 3 h, 50%.

The first family includes the known compounds FSM and FR9000098 and their corresponding esters (compounds **16** and **17**, Scheme 1), along with the tetraethylphosphonate dimer **18**. Synthesis of FSM and FR900098 was conducted as reported in the literature by Suresh et al. [36] and Uh et al. [37]. *tert*-Butyl *N*-(benzyloxy)carbamate **36** and the bromophosphonic diester **37** were synthesized in an 89% and 50% total yield, respectively, according to literature procedures [21,25]. Coupling of compounds **36** and **37** followed by TFA-mediated cleavage of the Boc protective group, yielded the key-intermediate synthon **39** in a 94% yield (two steps).

FSM was obtained in three steps and 72% total yield through formylation of the secondary amine of compound **39**, hydrogenolysis of the benzyl protective group affording compound **16**, and deprotection of the ethyl ester functions followed by a basic work-up.

Compounds **17** and FR900098 were obtained through a similar sequence of reactions involving acetylation of **39**, hydrogenolysis, and ethyl ester groups’ elimination. This sequence afforded better yields concerning acetylation and hydrogenolysis; thus compound **17** and FR900098 were obtained in a 68% and 83% yield, respectively. Based on this procedure, the FSM dimer **18** was synthesized (Scheme 1) by introducing a succinic acid spacer between the two fragments. Thus, compound **39** was allowed to react with succinic anhydride in the presence of a catalytic amount of 4-(dimethylamino)pyridine (DMAP), providing the acid **42** in an 88% yield. Then, coupling of **42** with the amine **39** was performed using the system HBTU/Et_3_N in chloroform to give the protected fosmidomycin dimer **43** in a 61% yield. Cleavage of the benzyl groups by cat. hydrogenolysis led to the tetraethyl phosphonate **18** in moderate yield (50%, Scheme 1).

### 2.2. Synthesis of FSM-ACQ **19**–**20** and FSM-ART Conjugates **21**–**23**

**Scheme 2:**
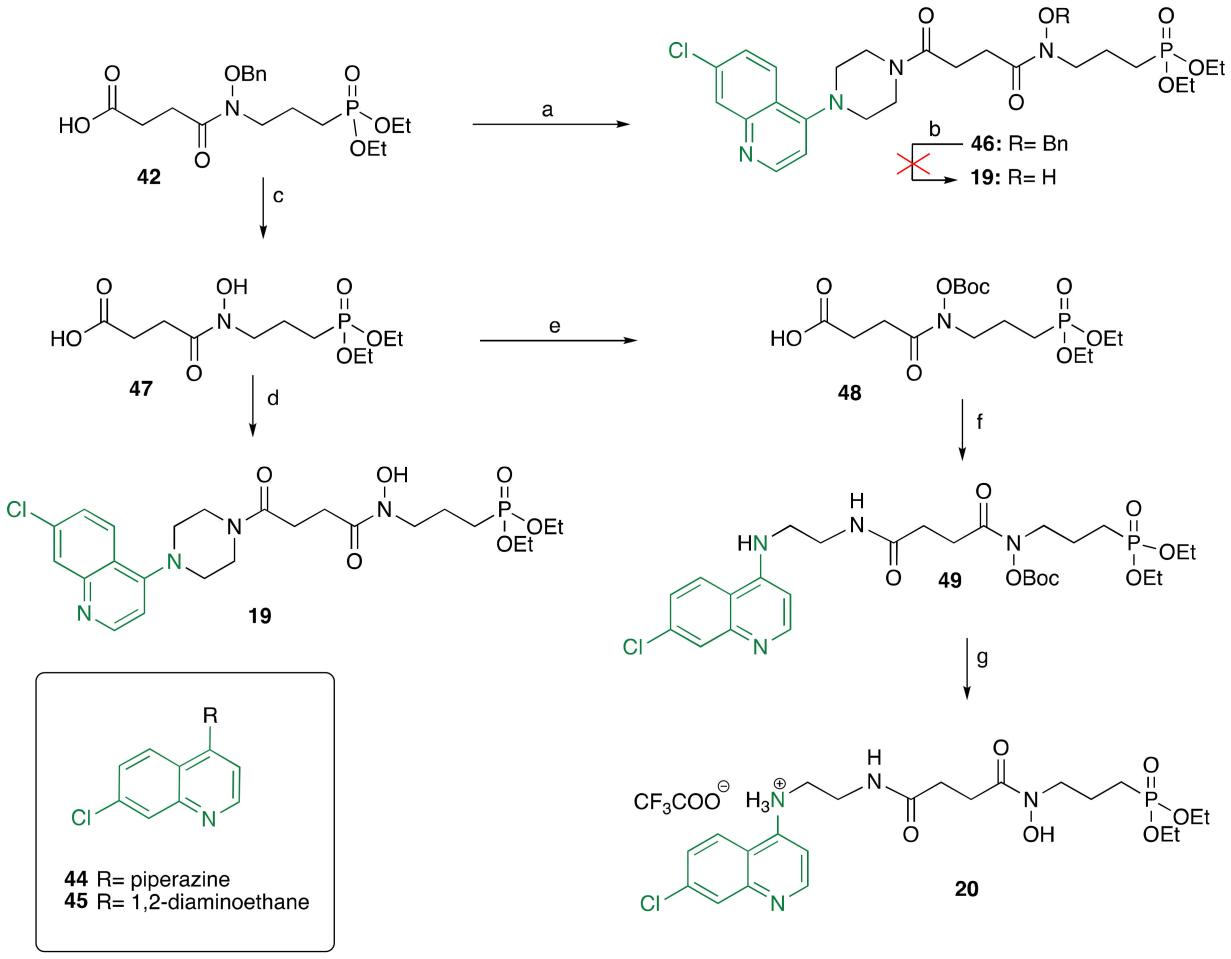
Synthesis FSM-ACQ conjugates **19** and **20**; Reagents and conditions: (a) **44**, HBTU, Et_3_N, CHCl_3_, rt, 3.5 h, 61% (b) H_2_, 10% Pd/C, MeOH, rt, 3 h; (c) H_2_, 10% Pd/C, MeOH, rt, 5 h, 97%; (d) **44**, HBTU, DIPEA, CHCl_3_, rt, 2 h, 60%; (e) Boc_2_O, Et_3_N, DMAP, CH_2_Cl_2_, rt, overnight, 30%; (f) **45**, HBTU, Et_3_N, CHCl_3_, DMF, 4 h, 50%; (g) TFA, CH_2_Cl_2_, overnight, 60%.

**Scheme 3:**
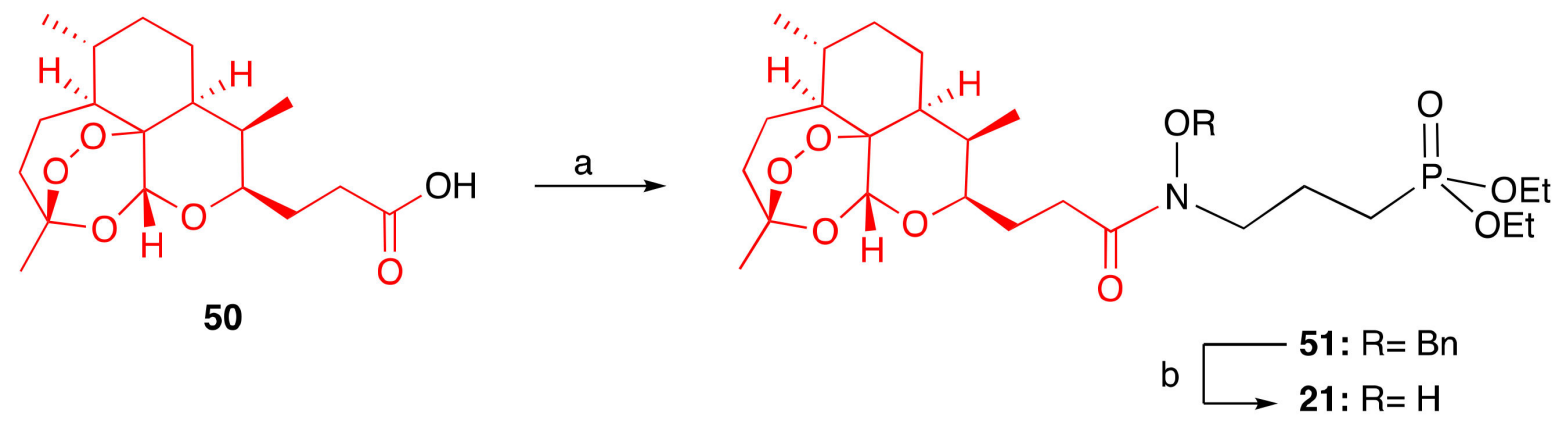
Synthesis of FSM-ART conjugate **21**; Reagents and conditions: (a) **39**, HBTU, Et_3_N, CH_2_Cl_2_, overnight, 50%; (b) H_2_, 10% Pd/C, MeOH, rt, 4 h, 90%.

**Scheme 4:**
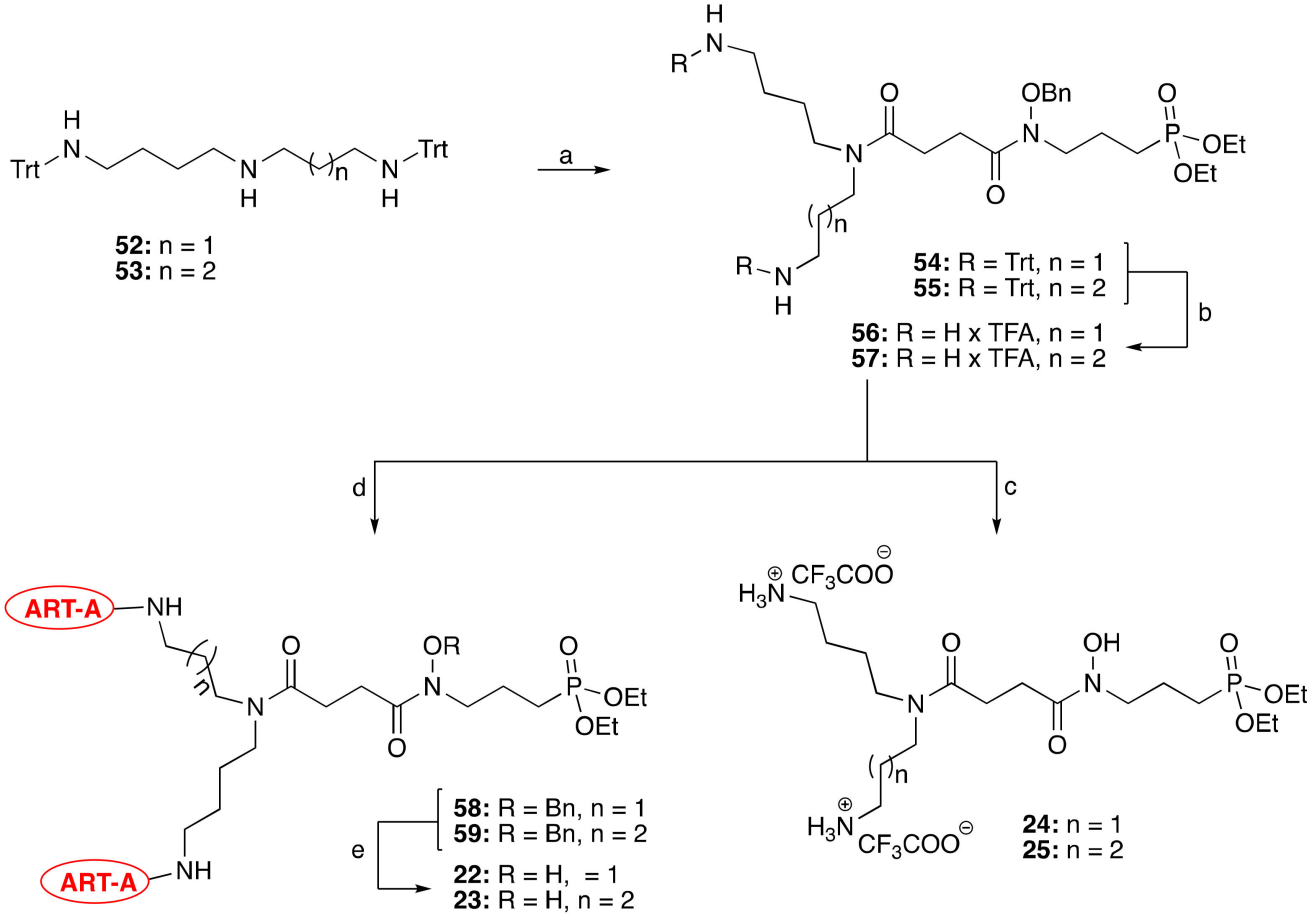
Synthesis of the FSM-ART conjugates **22**–**23** and FSM derivatives **24**–**25**; Reagents and conditions: (a) **42**, HBTU, DIPEA, CHCl_3_, rt, 7 h (72% for **54** and 96% for **55**) (b) TFA, TFE, CH_2_Cl_2_, 5 h (90% for **56** and 98% for **57**); (c) H_2_, 10% Pd/C, MeOH, rt, 6.5 h (91% for **24** and 95% for **25**); (d) 2× **50**, HBTU, DIPEA, CH_2_Cl_2_, overnight, 50%; (e) H_2_, 10% Pd/C, MeOH, rt, 4 h (60% for **22** and 80% for **23**).

Concerning the second family of compounds, namely the FSM conjugates, the FSM core is attached through a linker to either 4-amino-7chloro-quinoline (ACQ) or artemisinin frames. In this respect, five compounds were synthesized, two of them bearing the ACQ and three the ART moiety (compounds **19**–**20** (Scheme 2) and **21**–**23**, respectively, Scheme 3 and Scheme 4). For the FSM-ACQ conjugates, two different ACQ amine derivatives (**44** and **45**), bearing a piperazine or an ethylenediamine linker attached in position 4 of the 4,7-dichloroquinoline [38], were prepared. While coupling of *O*-benzyl compound **42** with the piperazino chloroquinoline **44** worked well, the following cleavage of the benzyl group proved to be harsh, affording a mixture of compounds, where hydrogenolysis of the quinoline group was also observed. By reversing the sequence of reactions, thus first performing hydrogenolysis and then coupling of the hydroxamic acid **47** with piperazine **44**, the desired hybrid **19** was obtained in a 58% yield over two steps.

For the second hybrid, possessing an ethylene diamine linker, this sequence of reactions could not be applied. A complex mixture of compounds was obtained that was difficult to purify. In this respect, the Boc group was used as alternative protection for the hydroxamic function; treatment of **47** with the system Boc_2_O/Et_3_N/DMAP afforded acid **48** and then followed a coupling reaction with the primary amino group of compound **45** to give the desired compound **49** in a 50% yield, after FCC purification. Boc-deprotection, by TFA-mediated acidolysis, yielded the FSM-ACQ hybrid **20** in a 60% yield.

For FSM-ART conjugates, one artemisinin fragment was first introduced through the acid derivative **50** (Scheme 3)**,** which was prepared as previously described [35]. Reaction of compound **50** with benzyloxyamine **39** under peptide coupling conditions afforded derivative **51**, which upon hydrogenolysis reaction led to FSM-ART hybrid **21** in a 45% yield over 2 steps, after FCC purification.

Moreover, based on our recent work, where hybrids possessing two artemisinin moieties linked with polyamine spacers, such as spermidine or homospermidine, present very good activities against *Plasmodium falciparum* [28], compounds **22** and **2**, possessing a FSM moiety attached through a spermidine or homospermidine linkers to two artemisinin moieties, were synthesized. Protected spermidine and homospermidine derivatives **52** and **53**, respectively, were prepared according to the procedure described in our previous work [35]. Reaction of **52** and **53** with the acid **42** under different and optimal coupling conditions afforded compounds **54** and **55** in 72% and 96% yields, respectively. One-pot deprotection of both benzyl and trityl groups under catalytic hydrogenation conditions unfortunately afforded a mixture of compounds. We thus proceeded in two steps: first with TFA-mediated acidolysis of the trityl groups, followed by hydrogenation for the benzyl group cleavage. The two-step procedure afforded the valuable intermediates **24** and **25** in 91% and 95% yields, respectively (Scheme 4).

Attempts to couple compounds **24** or **25** with the artemisinin acid derivative **50** as for the ACQ derivatives failed to proceed in a clean manner; a mixture of derivatives bearing two and three artemisinin moieties appeared in the mass spectrum, also indicating coupling on the N-OH function. So, precursors **56** and **57** were allowed to react with artemisinin acid **50** (Scheme 4) using HBTU as the coupling agent, in order to afford the benzylated derivatives **58** and **59** in a 50% yield. The latter were then hydrogenated, leading to the final FSM-ART hybrids **22** and **23** in 60% and 80% yields, respectively, after flash column chromatography (FCC) purification.

### 2.3. Synthesis of FSM Derivatives **26–35**

**Scheme 5:**
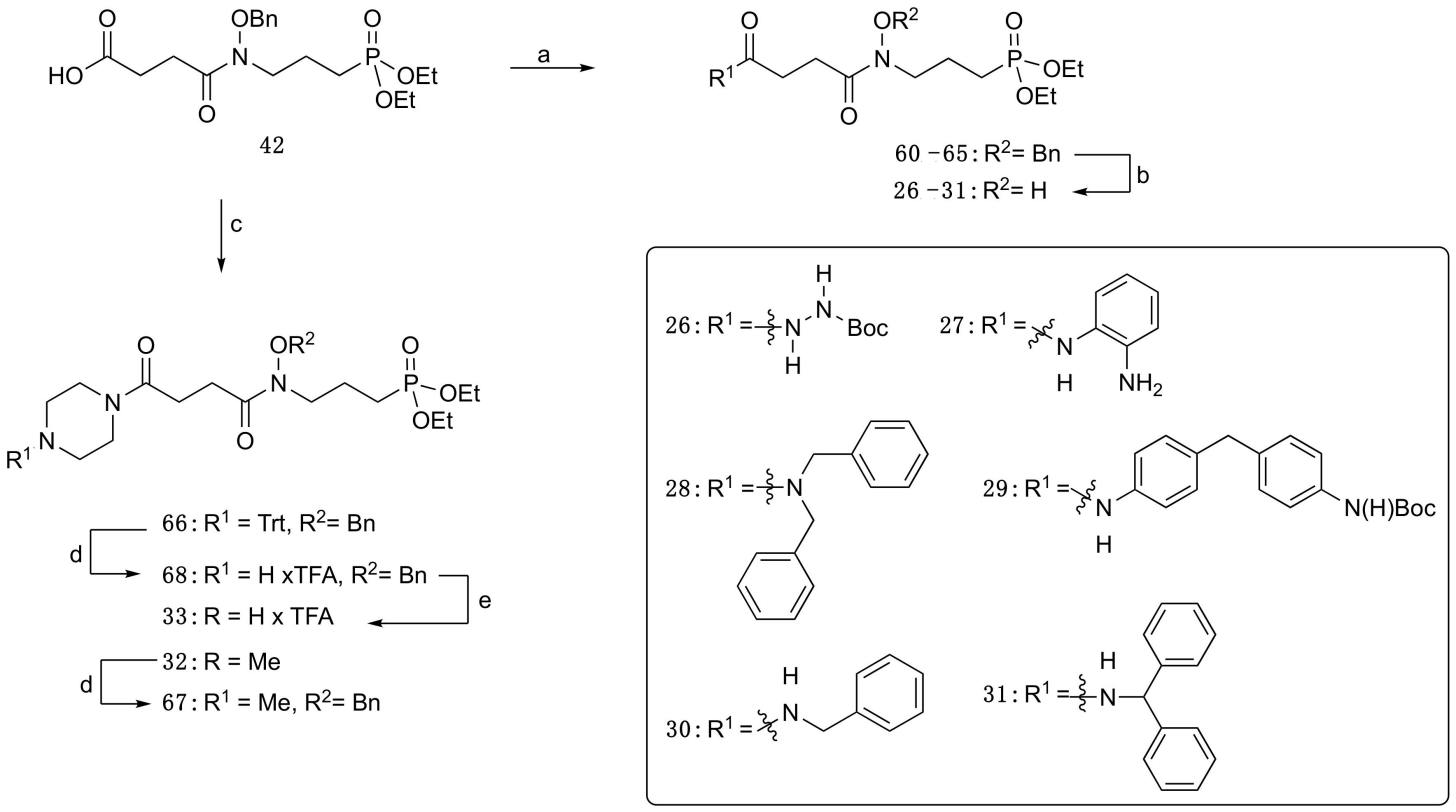
Synthesis of FSM-amido derivatives **26**–**33**; Reagents and conditions: (a) **R^1^H**, HBTU, DIPEA, CH_2_Cl_2_, rt, 3–6 h, 62%–84%; (b) H_2_, 10% Pd/C, MeOH, rt, 3–5 h, 80%–90%; (c) **R^1^H**, HBTU, DIPEA, CH_2_Cl_2_, rt, 2 h, 70% for **66** and 6 h, 84% for **68**; (d) TFA, TFE, CH_2_Cl_2_, 0 °C to rt, 1 h, 70%; (e) H_2_, 10% Pd/C, MeOH, rt, 2 h, 90% for **32** and 4 h, 60% for **33**.

The third family of FSM derivatives synthesized is based on the functionalization of the acid **42**. This compound can be an interesting platform, where the main functions of FSM are present, and a propyl carboxylic acid is attached as a linker. In this respect, the two first compounds of this family (**56** and **57**, Scheme 4) have already been synthesized as valuable intermediates in the synthesis of FSM-ART conjugates with spermidine and homospermidine linkages. By using analogous peptide coupling conditions, eight new compounds were synthesized possessing various amino and/or amido frameworks connected to the hydroxamic function (Scheme 5).

Among the different amine reagents, Boc-monoprotected dianiline (used for derivative **29**) and Trt-piperazine (used for derivative **66**) were synthesized according to known procedures [39,40], while all other amines were commercially available.

**Scheme 6:**
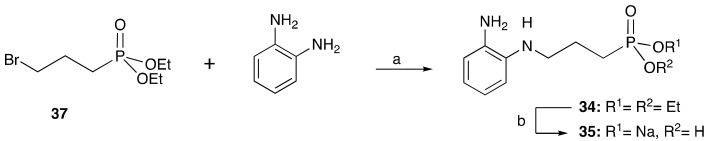
Synthesis of phosphonate derivatives **34** and **35**; Reagents and conditions: (a) DBU, DMF, 1 h, ultrasound, rt, 33%; (b) (i) TMSBr, DCM, 0 °C to rt, 8 h (ii) 6N aq. NaOH/MeOH 75% over 2 steps.

Finally, two more compounds were synthesized, where the hydroxamic part was replaced by a fragment that potentially can interact with metal ions as is the case and the role of the hydroxamic function of FSM. In this respect, *o*-phenylenediamine (a known pharmacophore) was chosen due to its chelating capacity [41]. Synthesis of the two final compounds **34** and **35** was straightforward. Alkylation of *o*-phenylenediamine under sonicating conditions using the already available bromophosphonate **37** afforded in fair yield compound **34**, which upon ethyl ester deprotection, followed by basic work-up, led to compound **35** (Scheme 6).

**Figure 3:**
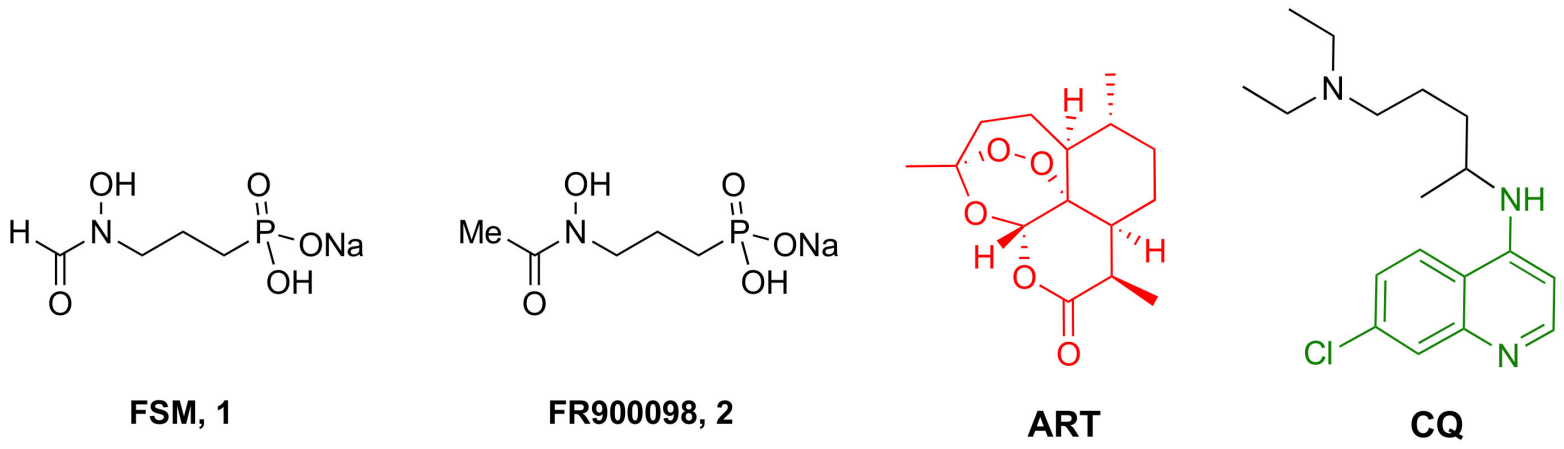
Structures of the control drugs (FSM, FR900098, ART, CQ).

The synthesized compounds were evaluated for their antiplasmodial activity against the CQ-resistant *P. falciparum* FcB1/Colombia strain [42,43], using as control drugs FSM, FR900098, ART, and CQ (see Figure 3). Moreover, their cytotoxicity was measured, as previously reported, upon the primary human fibroblast cell line AB943, which allowed the calculation of their selectivity index (SI) [35]. 

Specifically, IC_50_ (μM) values for the antiplasmodial activities were determined from four independent experiments as the mean values ± standard deviations. IC_50_ (μM) values for the cytotoxicity activities were determined from two independent experiments as the mean values ± standard deviations. IC_50,_ SIs (ratio between IC_50_ (μM) of cytotoxicity/IC_50_ (μM) of the antiplasmodial activity), and XLogGP3 values for the FSM derivatives, hybrid, and conjugates are reported in Table 1. The IC_50_ values of ART, FSM, FR900098, and CQ were also measured and are provided as controls.

Concerning the first family of compounds (FSM, **17**, FR900098, **18**), it was noticed that phosphonate esters are inactive. For example, FR900098′s diethyl phosphonate analogue **17** and the dimer **18**, which possesses two diethylphosphonate ester FSM frames linked together with a succinic acid spacer, have IC_50_ values >100 μΜ. FSM appears 2-fold less active than FR900098, with IC_50_ values of 15.0 and 7.0 μM respectively, while their selectivity indexes also follow the same trends (>6.6 vs. >14.2 μM respectively).

The second family, constructed by introducing next to the carbonyl group of the FSM hydroxamate group active antimalarial pharmacophores like aminochloroquinoline or artemisinin, presents very interesting results. The hybrid compounds with aminochloroquinoline **19** and **20** showed strong and comparable activities of 2.7 and 4.2 μM, respectively. Compound **19** possesses the structure of piperazine linked through an amide chain to the FSM diester fragment. The **19** and **20** compounds are 3.5–5.5 times more active than FSM itself and more than 25–40-fold more active in comparison to the diester compound **17**. Their activities are lower but closer to the CQ, indicating a major influence of the ACQ fragment, largely compensating the absence of acid functionality on the FSM. In addition, they showed higher selectivity indexes in comparison to FSM but much lower in comparison to CQ.

Concerning the hybrid compounds containing artemisinin, we observed a weaker activity for compound **21**, where one artemisinin moiety is linked directly to the FSM diester fragment (IC_50_ = 28.0 μM). On the contrary, the conjugation of the two ART frames with one FSM diester fragment through a spermidine and to a lesser extent to an homospermidine linker (**22**, **23**) afforded the most active molecules of our series with IC_50_ values of 0.36 and 0.65 μM; and the best selectivity among all new compounds, with SI values of 94 and 34, respectively. Here, again, it is observed that the IC_50_ values are 6.5–12 times higher than ART itself but much lower than FSM’s (23–41 fold). These values indicate a stronger influence of the ART fragment to the conjugates. It is noteworthy that all molecules of this family, which exhibit strong antimalarial activities, possess the diethyl phosphonate group.

According to our recently reported work on other antimalarial hybrid compounds [35], the presence of two artemisinin moieties linked through spermidine or homospermidine to an amide chain bearing a third endoperoxide (GMeP) gave IC_50_ values of the order of 10 nM. In the case of compounds **22** and **23**, the presence of FSM diester (in the place of GMeP) was observed again, even if the activities were lower in this case, probably confirming that hybrid compounds with two different potential targets of *Plasmodium* can be an interesting option in the search for new antimalarial compounds.

Compounds possessing only the spermidine or homospermidine fragments attached through an amido linker to FSM diester (**24**, **25**) are much less active; however, a better IC_50_ value was observed for compound **25** bearing the homospermidine frame. Among the other compounds of this third family, those bearing two aromatic rings attached to the amido function (**28**, **29**, **31**) present activities in the range between 22 and 44 μM, while compounds **26**, **27**, and **30** are inactive. Nevertheless, the second-best compound of this family series (**29**) is cytotoxic since it has an SI value of only 1.7. Finally, the two compounds possessing a piperazine or *N*-methyl piperazine fragment (**32**, **33**) are inactive, indicating that piperazine alone (as spermidine and homospermidine alone) lacks activity like the diester **17**. For the last two compounds of the third family (**34**, **35**), where the hydroxamic fragment was replaced by an *ortho*-benzenediamine fragment, we observed good activity for the diester **34** (IC_50_ = 8.2 μM), while the mono sodium salt of the corresponding diacid (**35**) is inactive. However, despite the good antimalarial activity of **34**, it seems to be cytotoxic, with an SI value of only 3.7.

In Table 1, the MWs, IC_50_ values, and the evaluation of the XLogGP3 SwissADME values according to the online calculation [44] are also reported. The software can combine physicochemical properties along with pharmacokinetics predictions for the drug likeness of the examined compounds. Since the families of the synthesized and tested compounds are quite different, we first examined and compared the values for each family. The active and known compounds FSM and FR900098 show the most negative values (−2.22 and −2.24, respectively) in comparison to values of −0.43 and −1.22 for diester **17** and the dimer **18**. For the second and most promising family of compounds, values vary between +1.42 and +5.33, with the two most active compounds having the highest ones. Concerning the third family of compounds, the most active show values between +1.92 and +2.80, while the inactive ones have negative values. In summary, apart from the first family, where all compounds possess negative XLOGP3 ADME values, the others show positive values for the active ones, the highest being for the most actives. Even if the calculation of the XLOGP3 values takes into consideration a number of different parameters, including the molecular weight, we feel that XLOGP3 could be one of many valuable tools to include when searching to optimize structures of the same family.

Results concerning comparative activities and the XLogGP3 SwissADME for all compounds tested indicated that different factors intervene for the activities of the compounds. Most important of them are related to the fragment-attached FSM derivative and to the linker next to that. The influence of the acid group of the FSM has to be more extensively studied, as its rol is not apparent in regard to some of the results obtained. Moreover, it is noteworthy to observe that compounds with a potential protonation site on the fragment adjacent to the FSM core (compounds **19**, **20**, **34**) can be very active, along with those possessing two highly active pharmacophores (compounds **22**, **23**) connected through a polyamino linker to the FSM core.

In conclusion, a series of FSM analogs, derivatives, and conjugates with ART and ACQ were synthesized and evaluated against *P. falciparum*. The most active compounds, which in addition to possessing a good SI, are those that combine two ART frames with the one of FSM diester, through a spermidine or homospermidine linker and exhibit IC_50_ values of 0.36 and 0.65 μM, respectively (compounds **22** and **23**). Interestingly, the hybrid compounds with one ACQ frame (compounds **19** and **20**) are 3.5–5.5 times more active than FSM itself with fairly good SIs. While for FSM and FR900098 the monosodium salt **17** is the most active form, for the hybrid compounds mentioned above, the diethyl phosphonates **19** and **20** show the best activity. 

Further work is in progress to examine whether for the most active compounds, (i) the diester forms are better than the acidic ones and (ii) if they are active against strains of *P. falciparum* showing a different resistance status. Finally, according to our recent findings on hybrid compounds possessing the endoperoxide GMeP [35], we are now engaged in incorporating this frame in a series of FSM analogues and evaluating them. 

## 3. Materials and Methods

### 3.1. General Methods

Melting points were determined with a Buchi SMP-20 apparatus and are uncorrected. ^1^H NMR spectra were obtained at 600.13 MHz and ^13^C NMR spectra at 150.90 MHz on a Bruker AVANCEIII HD spectrometer. Chemical shifts (*δ*) are indicated in parts per million (ppm) downfield from TMS and coupling constants (*J*) are reported in hertz (Copies of NMR spectra are available in the Supplementary Materials. ESI-HRMS spectra were recorded on a Waters Q-TOF UPLC Xevo G2 and ESI mass spectra were recorded at 30 V, on a Micromass-Platform LC spectrometer using MeOH as solvent. All solvents were dried and/or purified according to standard procedures prior to use. Anhydrous Na_2_SO_4_ was used for drying solutions, and the solvents were then routinely removed at ca. 40 °C under reduced pressure using a rotary vacuum evaporator. All reagents employed in the present work were commercially available and used without further purification. When required, reactions were carried out under dry argon atmosphere in preflamed glassware. Flash column chromatography (FCC) was performed on Merck silica gel 60 (230–400 mesh) and analytical thin layer chromatography (TLC) was performed on Merck silica gel 60F_254_ (0.2 mm) precoated on aluminum foil. Spots on the TLC plates were visualized with UV light at 254 nm and ninhydrine solution or charring agents.

### 3.2. Experimental Procedures

#### 3.2.1. Synthesis of FSM Dimer **18**

4-((Benzyloxy)(3-(diethoxyphosphoryl)propyl)amino)-4-oxobutanoic acid (**42**): To an ice-cold solution of protected amine **39** (1 mmol) and 4-dimethylaminopyridine (DMAP) (0.1 mmol) in freshly distilled tetrahydrofuran (THF) (2 mL), *N,N*-diisopropylethylamine (DIPEA) (1.5 mmol) was added. Then, the addition of succinic anhydride (1.1 mmol) was followed in small portions over 30 min and the reaction mixture was stirred at ambient temperature for 3h. Upon completion of the reaction, THF was removed under reduced pressure and the residue thus obtained was diluted with CH_2_Cl_2_ and washed with 5% aqueous citric acid, water, and brine. The organic layer was dried over Na_2_SO_4_ and evaporated to dryness under vacuum. The residue was subjected to FCC to give acid 42 as yellow oil (283 mg, 88%); R_f_ (CHCl_3_/MeOH 95:5): 0.15; ^1^H NMR (CDCl_3_): *δ* 7.37 (5H, s), 4.84 (2H, s), 4.16–3.98 (4H, m), 3.71 (2H, t, *J* = 6.2 Hz), 2.71 (2H, t, *J* = 6.4 Hz), 2.62 (2H, t, *J* = 6.4 Hz), 1.98–1.86 (2H, m), 1.79–1.68 (2H, m), 1.29 (6H, t, *J* = 7.1 Hz); ^13^C NMR (CDCl_3_): *δ* 175.9, 134.3, 129.3, 129.1, 128.8, 76.5, 61.9, 29.0, 28.7, 27.3, 23.2, 22.3, 20.2, 16.4; ^31^P NMR (CDCl_3_): *δ* 34.18; ESI-MS (30eV): *m*/*z* 825.04 [2M + Na]^+^, 440.16 [M + K]^+^, 424.16 [M + Na]^+^, 402.12 [M + H]^+^.

Tetraethyl ((4,7-dioxo-1,10-diphenyl-2,9-dioxa-3,8-diazadecane-3,8-diyl) bis(propane-3,1-diyl)) bis(phosphonate) (**43**): To a solution of acid **42** (60 mg, 0.15 mmol) and HBTU (63 mg, 0.165 mmol) in CHCl_3_ (1.4 mL), amine **39** (45 mg, 0.15 mmol), and Et_3_N (32 μL, 0.225 mmol) were added. After 3.5 h, the reaction mixture was diluted with CHCl_3_, washed with 5% aqueous citric acid, water, 5% aqueous NaHCO_3_, water, and brine. The organic layer was dried over Na_2_SO_4_, filtered, and evaporated to dryness under reduced pressure. The residue thus obtained was subjected to FCC, affording the corresponding pure conjugate; Yellow oil (72 mg, 70%); R_f_ (CHCl_3_/MeOH 95:5): 0.37; ^1^H NMR (CDCl_3_) *δ* 7.41–7.34 (m, 10H), 4.90 (s, 4H), 4.12–4.02 (m, 8H), 3.71 (t, *J* = 6.4 Hz, 4H), 2.77 (s, 4H), 1.99–1.89 (m, 4H), 1.75–1.69 (m, 4H), 1.29 (t, *J* = 7.1 Hz, 12H); ^13^C NMR (CDCl_3_) *δ* 175.5, 134.5, 129.2, 128.9, 128.7, 76.5, 61.6, 26.9, 23.5, 22.5, 20.3, 16.5; ESI-MS (30eV): *m*/*z* 723.42 [M + K]^+^, 707.54 [M + Na]^+^, 685.54 [M + H]^+^, 384.64 [M−C_14_H_23_NO_4_P]^+^.

Tetraethyl-((succinylbis(hydroxyazanediyl))bis (propane-3,1-diyl))bis(phosphonate) (**18**): A solution of **43** (50 mg, 0.073 mmol) in methanol (3 mL) was subjected to hydrogenolysis over 10% Pd/C (15 mg) at ambient temperature for 3 h. Thus, the reaction mixture was filtered through Celite and the filter cake was washed several times with methanol. After evaporation of the solvent to dryness under reduced pressure, the residue was subjected to FCC, affording the corresponding the dimer **18**; Yellow oil (18 mg, 50%); R_f_ (CHCl_3_/MeOH 95:5): 0.12; ^1^H NMR (CDCl_3_) *δ* 9.92 (s, 1H), 4.11–4.01 (m, 8H), 3.75–3.59 (m, 4H), 2.82 (s, 4H), 1.97–1.85 (m, 4H), 1.82–1.71 (m, 4H), 1.30 (t, *J* = 7.0 Hz, 12H); ^13^C NMR (CDCl_3_) *δ* 170.7, 62.0, 49.0 27.8, 22.9, 21.9, 19.5, 16.4; HRMS (ESI/Q-TOF): *m*/*z* 527.1907 [M + Na]^+^ for the compound C_18_H_38_N_2_O_10_P_2_ requires 527.1894.

#### 3.2.2. Synthesis of FSM-ACQ Derivatives **19** and **20**

4-((3-(Diethoxyphosphoryl)propyl)(hydroxy)amino)-4-oxobutanoic acid (**47**): A solution of **42** (80 mg, 0.2 mmol) in methanol (3 mL) was subjected to hydrogenolysis over 10% Pd/C (12 mg) at ambient temperature for 5 h. Thus, the reaction mixture was filtered through Celite and the filter cake was washed several times with methanol. After evaporation of the solvent to dryness under reduced pressure, the residue was subjected to FCC, affording pure **47**; Orange oil (60.4 mg, 97%); R_f_ (CHCl_3_/MeOH 9:1): 0.23; ^1^H NMR (CDCl_3_) *δ* 4.14–4.02 (m, 4H), 3.73 (t, *J* = 6.2 Hz, 2H), 2.86 (t, *J* = 6.2 Hz, 2H), 2.66 (t, *J* = 7.3 Hz, 2H), 2.00–1.90 (m, 2H), 1.83 & 1.80 (dt, *J* = 18.3, 7.1 Hz, 2H), 1.32 (t, *J* = 7.1 Hz, 6H); ^13^C NMR (CDCl_3_) *δ* 177.2, 173.7, 62.4, 47.9, 29.4, 27.4, 22.9, 22.7, 21.9, 19.1, 16.4; ESI-MS (30eV): *m*/*z* 661.14 [2M + K]^+^, 645.14 [2M + Na]^+^, 350.37 [M + K]^+^, 334.43 [M + Na]^+^.

Diethyl (3-(4-(4-(7-chloroquinolin-4-yl)piperazin-1-yl)-*N*-hydroxy-4-oxobutanamido) propyl) phosphonate (**19**): To a solution of acid **47** (32 mg, 0,1 mmol) and 2-(1Hbenzotriazol-1-yl)-1,1,3,3-tetramethyluronium hexafluorophosphate (HBTU) (42 mg, 0.1 mmol) in CHCl_3_ (0.2 mL), amine **44** (25 mg, 0.1 mmol) and DIPEA (30 μL, 0.15 mmol) were added at ambient temperature. After 2 h, the mixture was evaporated under reduced pressure and the residue thus obtained subjected to FCC providing the conjugate **19**; Orange oil (32 mg, 60%); R_f_ (CHCl_3_/MeOH 95:5): 0.12; ^1^H NMR (CDCl_3_) *δ* 9.82 (s, 1H), 8.75 (d, *J* = 5.0 Hz, 1H), 8.07 (d, *J* = 2.2 Hz, 1H), 7.95 (d, *J* = 9.0 Hz, 1H), 7.47 (dd, *J* = 9.0, 2.1 Hz, 1H), 6.85 (d, *J* = 5.0 Hz, 1H), 4.14–4.05 (m, 4H), 3.90 (s, 2H), 3.83–3.78 (m, 2H), 3.75 (t, *J* = 6.1 Hz, 2H), 3.25–3.16 (m, 4H), 2.88 (t, *J* = 6.4 Hz, 1H), 2.77 (t, *J* = 6.4 Hz, 1H), 2.03–1.90 (m, 2H), 1.80 (dt, *J* = 18.4, 7.0 Hz, 4H), 1.32 (t, *J* = 7.1 Hz, 6H); ^13^C NMR (CDCl_3_) *δ* 173.2, 171.3, 156.4, 151.9, 150.1, 135.2, 129.0, 126.7, 124.7, 121.8, 109.4, 62.0, 52.1, 47.6, 45.5, 41.8, 29.0, 27.2, 22.8, 21.9, 19.3, 16.4; HRMS (ESI/Q-TOF): *m*/*z* 541.1997 [M + H]^+^; for the compound C_24_H_34_ClN_4_O_6_P requires 541.1977.

4-(((Tert-butoxycarbonyl)oxy)(3-(diethoxy-phosphoryl)propyl)amino)-4-oxobutanoic acid (**48**): To a solution of **47** (55 mg, 0.176 mmol) in CH_2_Cl_2_ (5.5 mL), catalytic amount of DMAP, Et_3_N (25 μL, 0.176 mmol), and di-tert-butyl-dicarbonate (38 mg, 0.176 mmol) were added and the reaction mixture was stirred overnight at ambient temperature. It was then diluted with DCM and washed with pre-cooled 5% aqueous citric acid, water, and brine. The organic layer was dried over Na_2_SO_4_, filtered, and evaporated to dryness under reduced pressure. The residue thus obtained was subjected to FCC, affording **48** as yellow oil (21.7 mg, 30%); R_f_ (CHCl_3_/MeOH 95:5): 0.15; ^1^H NMR (CDCl_3_) *δ* 4.15–4.02 (m, 4H), 3.79 (t, *J* = 6.3 Hz, 2H), 2.67 (d, *J* = 6.2 Hz, 2H), 2.62 (d, *J* = 4.1 Hz, 2H), 1.90–1.84 (m, 2H), 1.83–1.75 (m, 2H), 1.54 (s, 9H), 1.31 (t, *J* = 7.1 Hz, 6H); ^13^C NMR (CDCl_3_) *δ* 175.1, 61.8, 61.7, 28.4, 27.6, 16.4; ^31^P NMR (CDCl_3_) *δ* 34.18.

Diethyl (3-(*N*-((tert-butoxycarbonyl)oxy)-4-((2-((7-chloroquinolin-4-yl)amino)ethyl) amino)-4-oxobutanamido)propyl)phosphonate (**49**): To a solution of acid **48** (13 mg, 0.03 mmol) and HBTU (13 mg, 0.033 mmol) in CHCl_3_/DMF 5:1 (0.3 mL), amine **45** (7 mg, 0.03 mmol), and Et_3_N (7 μL, 0.045 mmol) were added. After 4h, the reaction mixture was diluted with DCM, washed with pre-cooled 5% aqueous citric acid, water, 5% aqueous NaHCO_3_, water, and brine. The organic layer was dried over Na_2_SO_4_, filtered, and evaporated to dryness under reduced pressure. The residue thus obtained was subjected to FCC, affording the corresponding pure conjugate **49** as yellow oil (9.2 mg, 50%); R_f_ (CHCl_3_/MeOH 95:5): 0.18; ^1^H NMR (CDCl_3_) δ 8.41 (d, *J* = 5.6 Hz, 1H), 7.93 (d, *J* = 2.0 Hz, 1H), 7.91 (s, 1H), 7.39 (dd, *J* = 8.9, 2.0 Hz, 1H), 7.16 (s, 1H), 7.05 (s, 1H), 6.28 (d, *J* = 5.7 Hz, 1H), 4.13–4.01 (m, 4H), 3.67 (s, 4H), 3.44–3.38 (m, 2H), 2.69 (s, 2H), 2.56 (s, 2H), 1.83–1.75 (m, 2H), 1.74 (d, *J* = 13.1 Hz, 2H), 1.51 (s, 9H), 1.30 (t, *J* = 7.1 Hz, 6H); ^13^C NMR (CDCl_3_) δ 174.7, 151.3, 149.9, 135.8, 126.5, 125.8, 122.8, 117.0, 98.0, 61.7, 45.1, 38.6, 27.5, 20.2, 16.4; ESI-MS (30eV): *m*/*z* 1251.76 [2M + Na]^+^, 653.26[M + K]^+^, 637.49[M + Na]^+^, 615.52[M + H]^+^.

7-Chloro-*N*-(2-(4-((3-(diethoxyphosphoryl)propyl)(hydroxy)amino)-4oxobutanamido) ethyl)quinolin-4-aminium 2,2,2-trifluoroacetate (**20**): To an ice-cold solution of **49** (6 mg, 0.01 mmol) in CH_2_Cl_2_ (0.4 mL), trifluoroacetic acid (TFA) (12 μL) was added and the reaction mixture was stirred overnight at ambient temperature. Then, volatile components were evaporated under vacuum and the oily residue thus obtained was subjected to FCC, affording pure conjugate **20**. Colorless oil (3.8 mg, 60%); R_f_ (CHCl_3_/MeOH 85:15): 0.3; ^1^H NMR (CDCl_3_) δ 9.15 (s, 1H), 8.28 (d, *J* = 6.6 Hz, 1H), 8.15 (d, *J* = 9.0 Hz, 1H), 7.93 (s, 2H), 7.45 (d, *J* = 8.9 Hz, 1H), 6.40 (d, *J* = 6.8 Hz, 1H), 4.13–4.02 (m, 4H), 3.69–3.61 (m, 4H), 3.53 (s, 2H), 2.89 (t, *J* = 6.4 Hz, 2H), 2.59 (t, *J* = 6.1 Hz, 2H), 1.97–1.88 (m, 2H), 1,81–1.75 (m,23H), 1.32 (t, *J* = 7.0 Hz, 6H); ^13^C NMR (CDCl_3_) δ 176.0, 173.5, 155.6, 142.7, 139.8, 138.7, 127.9, 124.4, 120.0, 115.3, 97.5, 62.3, 47.9, 45.4, 38.1, 31.2, 29.7, 28.3, 22,6 21.8, 19.2, 16.4; HRMS (ESI/Q-TOF): m/z 537.1649 [M + Na]^+^; for the compound C_22_H_32_ClN_4_O_6_P requires 537.1640.

#### 3.2.3. Synthesis of FSM-ART Conjugates **21**–**23**

Diethyl (3-(*N*-(benzyloxy)-3-((3S,5aS,6R,8aS, 9R,10R,12R)-3,6,9-trimethyldecahydro-12H-3,12epoxy [1,2] dioxepino [4,3-i]isochromen-10-yl)propanamido) propyl) phosphonate (**51**): To a solution of artemisinin derivative **50** (25 mg, 0.07 mmol) in DCM (0.47 mL), HBTU (29 mg, 0.077 mmol) and Et_3_N (15 μL, 0.105 mmol) were added. The reaction mixture was stirred overnight at ambient temperature. Subsequently, it was diluted with CH_2_Cl_2_, washed with pre-cooled 5% aqueous citric acid, water, 5% aqueous NaHCO_3_, water, and brine. The organic layer was dried over Na_2_SO_4_, filtered, and evaporated to dryness under reduced pressure. The residue thus obtained was subjected to FCC, affording conjugate **51** as yellow oil (22 mg, 50%); R_f_ (AcOEt): 0.17; ^1^H NMR (CDCl_3_) δ 7.44–7.39 (m, 2H), 7.38–7.33 (m, 3H), 5.29 (s, 1H), 4.85 (s, 2H), 4.12–4.03 (m, 4H), 3.78–3.65 (m, 2H), 2.80 (s, 1H), 2.76–2.71 (m, 1H), 2.55–2.47 (m, 1H), 2.33 (td, *J* = 14.0, 3.9 Hz, 1H), 2.04–1.99 (m, 1H), 1.98–1.91 (m, 2H), 1.91–1.86 (m, 1H), 1.83–1.78 (m, 1H), 1.77–1.69 (m, 3H), 1.68–1.62 (m, 4H), 1.59–1.53 (m, 1H), 1.50–1.40 (m, 2H), 1.39 (s, 3H), 1.30 (t, *J* = 7.1 Hz, 6H), 1.27–1.20 (m, 2H), 0.94 (d, *J* = 6.1 Hz, 3H), 0.88 (d, *J* = 7.5 Hz, 3H); ^13^C NMR (CDCl_3_) δ 129.4, 128.9, 128.7, 103.4, 88.6, 81.2, 75.9, 61.6, 52.5, 44.6, 37.4, 36.6, 34.5, 30.2, 26.2, 24.9, 24.7, 24.0, 20.2, 16.5, 13.3; ESI-MS (30eV): *m*/*z* 1285.61 [2M + K]^+^, 1269.36 [2M + Na]^+^, 662.46 [M + K]^+^, 646.46 [M + Na]^+^.

Diethyl (3-(N-hydroxy-3-((3S,5aS,6R,8aS,9R,10R,12R)-3,6,9-trimethyldecahydro-12H-3, 12-epoxy[1,2]dioxepino[4,3-i]isochromen-10-yl)propanamido)propyl)phosphonate (**21**): A solution of conjugate **51** (20 mg, 0.032 mmol) in methanol (3 mL) was subjected to hydrogenolysis over 10% Pd/C (5 mg) at ambient temperature for 4 h. Thus, the reaction mixture was filtered through Celite and the filter cake was washed several times with methanol. After evaporation of the solvent to dryness under reduced pressure, the residue was subjected to FCC, affording pure **21**; Orange oil (15 mg, 90%); R_f_ (DCM/MeOH 95:5): 0.35; ^1^H NMR (CDCl_3_) δ 8.52 (br s, 1H), 8.08 (s, 1H), 5.28 (s, 1H), 4.10–4.01 (m, 4H), 3.87 (dt, *J* = 13.5, 6.6 Hz, 1H), 3.74–3.69 (m, 1H), 3.57–3.50 (m, 1H), 2.69–2.63 (m, 1H), 2.57–2.47 (m, 2H), 2.11 (s, 2H) 1.99–1.89 (m, 4H), 1.85–1.74 (m, 7H), 1.51 (s, 3H), 1.431–1.28 (m, 7H), 1.05 (d, *J* = 6.3 Hz, 3H), 0.96 (d, *J* = 6.7 Hz, 3H); ^13^C NMR (CDCl_3_) δ 175.0, 161.0, 107.6, 97.2, 82.6, 69.3, 61.7, 57.4, 54.3, 48.0, 45.2, 41.4, 40.0, 35.5, 34.5, 27.8, 25.1, 23.4, 22.0, 20.5, 19.7, 18.8, 16.4, 12.5, 11.9; HRMS (ESI/Q-TOF): *m*/*z* 556.2650 [M + Na]^+^; for the compound C_25_H_44_NO_9_P requires 556.2646.

General procedure for the synthesis of FSD-polyamine conjugates. To a solution of **42** (60 mg, 0.15 mmol) and HBTU (62 mg, 0.165 mmol) in CHCl_3_ (300 μL), the suitable protected spermidine **52** (94 mg, 0.15 mmol) or homospermidine **53** (96 mg, 0.15 mmol) and Et_3_N (32 μL, 0.225 mmol) were added. After 7–12 h, the mixture was diluted with CHCl_3_ and washed with pre-cooled 5% aqueous citric acid, water, 5% aqueous NaHCO_3_, water, and brine. The organic layer was dried over Na_2_SO_4_, filtered and evaporated to dryness under reduced pressure. The residues thus obtained were subjected to FCC, affording the corresponding conjugates **54** and **55**.

*Diethyl (3-(N-(benzyloxy)-4-oxo-4-((4-(tritylamino)butyl)(3-(tritylamino)propyl)amino)butanamido) propyl) phosphonate* (**54**): Reaction time: 7h; White foam (109 mg, 72%); R_f_ (AcOEt): 0.2; ^1^H NMR (CDCl_3_) *δ* 7.50–7.42 (m, 12H), 7.42–7.32 (m, 5H), 7.31–7.21 (m, 12H), 7.20–7.12 (m, 6H), 4.91 (s, 1H), 4.88 (s, 1H), 4.10–4.02 (m, 4H), 3.73–3.64 (m, 2H), 3.38 (t, *J* = 7.1 Hz, 1H), 3.33 (t, *J* = 7.7 Hz 1H), 3.25 (t, *J* = 7.1 Hz, 1H), 3.14 (t, *J* = 7.6 Hz, 1H), 2.76 (s, 1H), 2.60 (t, *J* = 6.0 Hz, 1H), 2.51 (t, *J* = 5.9 Hz, 1H), 2.18–2.10 (m, 3H), 2.08–2.05 (m, 1H), 1.97–1.87 (m, 2H), 1.74 (s, 3H), 1.72–1.65 (m, 2H), 1.60–1.55 (m, 1H), 1.53–1.42 (m, 3H), 1.30–1.25 (m, 6H); ^13^C NMR (CDCl_3_) *δ* 171.2, 146.2, 146.1, 145.9, 134.5, 129.1, 128.8, 128.6, 128.5, 127.8, 127.7, 126.3, 126.2, 126.1, 76.4, 70.8, 61.5, 60.4, 47.5, 46.2, 43.7, 43.5, 43.3, 41.3, 40.9, 38.6, 30.3, 28.7, 28.4, 28.1, 27.6, 27.3, 26.7, 25.8, 23.5, 22.5, 21.0, 20.2, 16.4, 14.2; ESI-MS (30eV): *m*/*z* 1035.75 [M + Na]^+^, 1013.39 [Μ + H]^+^, 301.48 [M−C_49_H_50_H_3_O_2_]^+^, 243.28 [Trt]^+^.

*Diethyl (3-(N-(benzyloxy)-4-(bis(4-(tritylamino)butyl)amino)-4-oxobutanamido) propyl) phosphonate* (**55**): Yellow pale foam (148 mg, 96%); R_f_ (AcOEt/Et_3_N 1%): 0.18; ^1^H NMR (CDCl_3_) *δ* 7.49–7.41 (m, 9H), 7.40–7.32 (m, 6H), 7.30–7.21 (m, 12H), 7.19–7.12 (m, 5H), 4.90 (s, 1H), 4.87 (s, 1H), 4.15–4.05 (m, 6H), 3.70 (dt, *J* = 14.0, 7.0 Hz, 2H), 3.22 (dt, *J* = 15.0, 7.1 Hz, 2H), 3.22–3.17 (m, 1H), 2.82–2.72 (m, 2H), 2.55 (t, *J* = 6.4 Hz, 1H), 2.12 (dt, *J* = 14.3, 7.0 Hz, 2H), 1.97–1.78 (m, 4H), 1.74–1.64 (m, 4H), 1.60–1.41 (m, 7H), 1.34–1.24 (m, 10H); ^13^C NMR (CDCl_3_) *δ* 171.2, 146.2, 146.1, 129.2, 128.6, 127.8, 127.7, 126.2, 70.2, 61.5, 60.6, 59.2, 47.7, 46.0, 43.4, 28.7, 28.4, 28.2, 27.7, 27.4, 26.7, 25.7, 24.0, 23.5, 23.1, 22.5, 20.3, 16.4, 14.2.

Trityl-deprotection of conjugates **54** and **55.** To an ice-cold solution of **54** or **55** (0.12 mmol) in DCM (1.0 mL), TFE (50 μL, 0.65 mmol) and TFA (50 μL, 0.65 mmol) were added. The reaction mixture was stirred at ambient temperature for 6h. Volatile components were evaporated under vacuo, and the oily residue was triturated with Et_2_O/Hex and refrigerated overnight. The corresponding trifluoroacetate salts **56** and **57** were received after decanting of the solvents.

*4-(N-(3-ammoniopropyl)-4-((benzyloxy)(3-(diethoxyphosphoryl)propyl)amino)-4-oxobutanamido)butan-1-aminium 2,2,2-trifluoroacetate* (**56**): Yellow oil (82 mg, 90%); ^1^H NMR (MeOD): δ 7.49–7.45 (m, 2H), 7.44–7.39 (m, 3H), 4.97 (br s, 2H), 4.13–4.05 (m, 4H), 3.78 (t, *J* = 6.5 Hz, 2H), 3.52–3.47 (m, 3H), 3.45 (t, *J* = 7.4 Hz, 1H), 3.03–2.97 (m, 2H), 2.90 (t, *J* = 7.0 Hz, 2H), 2.82 (br s, 2H), 2.71–2.65 (m, 2H), 1.94–1.86 (m, 4H), 1.85–1.68 (m, 6H), 1.65–1.61 (m, 1H), 1.31 (t, *J* = 7.1 Hz, 6H); ^13^C NMR (MeOD) δ 174.0, 172.8, 134.7, 129.2, 128.7, 128.3, 75.9, 65.5, 61.9, 44.4, 41.9, 39.0, 36.8, 36.5, 30.9, 27.1, 26.8, 26.3, 25.4, 25.2, 24.4, 24.2, 24.0, 22.2, 21.3, 19.7, 15.3, 15.3, 14.0, 12.9; HRMS (ESI/Q-TOF): m/z 461.2503 [M + Na]^+^; for the compound C_25_H_45_N_4_O_6_P requires 461.2499.

*4,4′-((4-((Benzyloxy)(3-(diethoxyphosphoryl)propyl)amino)-4-oxobutanoyl)azanediyl) bis(butan-1-aminium) 2,2,2-trifluoroacetate* (**57**): Yellow oil (83 mg, 90%); ^1^H NMR (MeOD): δ 7.48–7.44 (m, 2H), 7.42–7.36 (m, 3H), 4.96 (s, 2H), 4.11–4.04 (m, 4H), 3.76 (t, *J* = 6.6 Hz, 2H), 3.41 (t, *J* = 6.9 Hz, 2H), 3.37 (br s, 2H), 3.31–3.28 (m, 2H), 2.98 (t, *J* = 6.9 Hz, 2H), 2.94 (br s, 2H), 2.80 (s, 1H), 2.79 (br s, 1H), 2.67–2.63 (m, 2H), 1.93–1.86 (m, 2H), 1.83–1.76 (m, 2H), 1.75–1.66 (m, 4H), 1.62 (br s, 4H), 1.30 (t, *J* = 7.1 Hz, 6H); ^13^C NMR (MeOD) δ 172.7, 161.2, 160.9, 160.7, 160.5, 134.7, 129.2, 128.6, 128.3, 127.9, 127.2, 126.6, 119.0, 117.4, 115.5, 112.5, 75.9, 61.9, 46.8, 44.6, 39.0, 27.0, 25.2, 24.4, 24.3, 24.0, 22.2, 21.3, 19.7, 15.3; HRMS (ESI/Q-TOF): *m*/*z* 453.2848 [M + H]^+^; for the compound C_26_H_47_N_4_O_6_P requires 453.2836.

General procedure for the synthesis of ART-FSD conjugates **58** & **59.** To a solution of **50** (20 mg, 0.06 mmol) and HBTU (25 mg, 0.066 mmol) in CHCl_3_ (300 μL), compound **56** (22 mg, 0.03 mmol) or **57** (21 mg, 0.03 mmol), and Et_3_N (20 μL, 0.135 mmol) were added. After 12 h, the mixture was diluted with CHCl_3_ and washed with pre-cooled 5% aqueous citric acid, water, 5% aqueous NaHCO_3_, water, and brine. The organic layer was dried over Na_2_SO_4_, filtered, and evaporated to dryness under reduced pressure. The residues thus obtained were subjected to FCC, affording the corresponding conjugates **58** and **59**.

*Diethyl(3-(N-(benzyloxy)-4-oxo-4-((4-(3-((3S,5aS,6R,8aS,9R,10R,12R)-3,6,9-trimethyldecahydro-12H-3,12-epoxy[1,2]dioxepino[4,3-i]isochromen-10-yl)propanamido)butyl)(3-(3-((3S,5aS,6R,8aS,9R,10R,12R)-3,6,9-trimethyldecahydro-12H-3,12-epoxy[1,2]dioxepino[4,3-i]isochromen-10-yl)propanamido)propyl)amino) butanamido)propyl)phosphonate* (**58**): Yellow oil (18 mg, 50%); R_f_ (CHCl_3_/MeOH 95:5): 0.3; ^1^H NMR (CDCl_3_) δ 7.43–7.35 (m, 5H), 5.29 (s, 2H), 4.92 (s, 2H), 4.12–3.98 (m, 6H), 3.70 (s, 2H), 3.44–3.35 (m, 2H), 3.33–3.23 (m, 5H), 3.20–3.13 (m, 2H), 2.81 (s, 2H), 2.74–2.68 (m, 2H), 2.66–2.57 (m, 2H), 2.51–2.38 (m, 2H), 2.36–2.24 (m, 4H), 2.05–1.98 (m, 3H), 1.96–1.85 (m, 6H), 1.77 (s, 8H), 1.70–1.60 (m, 7H), 1.58–1.51 (m, 4H), 1.48–1.43 (m, 3H), 1.41–1.35 (m, 7H), 1.29 (t, *J* = 7.0 Hz, 6H), 1.26–1.20 (m, 3H), 0.94 (t, *J* = 5.8 Hz, 6H), 0.87 (t, *J* = 7.3 Hz, 6H); ^13^C NMR (CDCl_3_) δ 173.1, 173.0, 172.2, 129.2, 128.9, 128.7, 103.4, 88.7, 81.2, 61.6, 52.5, 47.3, 44.6, 44.5, 43.0, 38.9, 37.3, 36.5, 36.2, 34.6, 34.4, 30.2, 27.4, 27.0, 26.2, 24.9, 24.6, 20.2, 16.5, 13.2; ESI-MS (30eV): m/z 1211.62 [M + K]^+^, 1195.80 [M + Na]^+^.

*Diethyl(3-(N-(benzyloxy)-4-(bis(4-(3-((3S,5aS,6R,8aS,9R,10R,12R)-3,6,9-trimethyldecahydro-12H-3,12-epoxy[1,2]dioxepino[4,3-i]isochromen-10-yl)propanamido)butyl)amino)-4-oxobutanamido)propyl)phosphonate* (**59**): White foam (18 mg, 50%), R_f_ (CHCl_3_/MeOH 95:5): 0.3; ^1^H NMR (CDCl_3_) δ 7.44–7.32 (m, 5H), 6.32 (s, 1H), 6.21 (s, 1H), 5.28 (s, 2H), 4.91 (s, 2H), 4.13–3.98 (m, 6H), 3.69 (s, 2H), 3.36–3.19 (m, 8H), 2.80 (s, 4H), 2.74–2.67 (m, 2H), 2.60 (s, 2H), 2.47–2.40 (m, 2H), 2.35–2.24 (m, 4H), 2.04–1.97 (m, 2H), 1.95–1.85 (m, 8H), 1.83–1.77 (m, 4H), 1.75–1.70 (m, 2H), 1.67–1.60 (m, 4H), 1.58–1.51 (m, 7H), 1.49–1.42 (m, 4H), 1.37 (d, *J* = 3.4 Hz, 6H), 1.29 (t, *J* = 7.0 Hz, 6H), 1.25–1.21 (m, 3H), 0.96–0.91 (m, 6H), 0.89–0.83 (m, 6H); ^13^C NMR (CDCl_3_) δ 173.1, 171.4, 134.5, 129.2, 128.9, 128.7, 103.4, 88.7, 81.2, 61.6, 52.5, 47.5, 45.6, 44.5, 38.8, 38.6, 37.4, 36.5, 34.5, 34.4, 30.2, 27.6, 27.4, 27.0, 26.7, 26.1, 25.0, 24.8, 24.8, 24.6, 23.4, 22.5, 20.2, 16.5, 13.2; ESI-MS (30eV): m/z 1225.82 [M + K]^+^, 1210.29 [M + Na]^+^.

General procedure for the hydrogenolysis of **56**–**59**. A solution of **56** or **57** or **58** or **59** (0.01 mmol) in methanol (3 mL) was subjected to hydrogenolysis over 10% Pd/C (5 mg) at ambient temperature and pressure for 4 h–6.5 h. Thus, the reaction mixture was filtered through Celite and the filter cake was washed several times with methanol. After evaporation of the solvent to dryness under reduced pressure, the residues were subjected to FCC, affording the corresponding pure deprotected molecules **24**, **25**, **22**, and **23**, respectively**.**

*Diethyl(3-(N-hydroxy-4-oxo-4-((4-(3-((3S,5aS,6R,8aS,9R,10R,12R)-3,6,9-trimethyldecahydro-12H-3,12-epoxy[1,2]dioxepino[4,3-i]isochromen-10-yl)propanamido)butyl)(3-(3-((3S,5aS,6R,8aS,9R,10R,12R)-3,6,9-trimethyldecahydro-12H-3,12-epoxy[1,2]dioxepino[4,3-i]isochromen-10-yl)propanamido)propyl)amino) butanamido)propyl)phosphonate* (**22**): Yellow oil (6.5 mg, 60%), R_f_ (CHCl_3_/MeOH 97:3): 0.35; ^1^H NMR (CDCl_3_) δ 9.94 (br s, 1H), 8.18 (br s, 1H) 5.33 (s, 2H), 4.23–4.07 (m, 6H), 3.79 (s, 2H), 3.49–3.33 (m, 7H), 3.24–3.19 (s, 3H), 2.89 (br s, 2H), 2.79 (br s, 2H), 2.45–2.37 (m, 3H), 2.36–2.27 (m, 4H), 2.21 (s, 2H), 2.03–1.96 (m, 5H), 1.95–1.90 (m, 4H), 1.88–1.83 (m, 5H), 1.78–1.72 (m, 8H), 1.55 (s, 5H), 1.49 (t, *J* = 7.4 Hz, 3H), 1.40 (t, *J* = 6.9 Hz, 8H), 1.34 (s, 4H), 1.58–1.13 (m, 2H), 1.06–1.02 (m, 3H), 0.99–0.94 (m, 9H); ^13^C NMR (CDCl_3_) δ 213.4, 209.3, 173.7, 160.5, 107.3, 97.4, 82.7, 68.3, 62.2, 54.6, 47.9, 46.3, 45.6, 41.8, 40.8, 39.0, 35.9, 34.9, 34.1, 30.2, 29.9, 27.6, 25.5, 24.1, 24.0, 22.5, 20.8, 20.6, 19.1, 16.8, 12.9, 12.4, 8.9; HRMS (ESI/Q-TOF): m/z 1083.6273 [M + H]^+^; for the compound C_54_H_91_N_4_O_16_P requires 1083.6240.

*Diethyl(3-(4-(bis(4-(3-((3S,5aS,6R,8aS,9R,10R,12R)-3,6,9-trimethyldecahydro-12H-3,12-epoxy[1,2]dioxepino[4,3-i]isochromen-10-yl)propanamido)butyl)amino)-N-hydroxy-4-oxobutanamido)propyl)phosphonate* (**23**): Yellow oil (6.6 mg, 60%), R_f_ (CHCl_3_/MeOH 97:3): 0.35; ^1^H NMR (CDCl_3_) δ 9.97 (br s, 1H), 8.09 (s, 1H) 5.24 (s, 2H), 4.12–4.04 (m, 6H), 3.70 (br s, 2H), 3.31–3.22 (m, 7H), 3.15–3.08 (m, 2H), 2.78–2.69 (m, 4H), 2.35–2.29 (m, 3H), 2.24–2.18 (m, 4H), 2.12 (s, 2H), 2.08–1.99 (m, 4H), 1.94–1.88 (m, 7H), 1.78–1.74 (m, 4H), 1.67–1.64 (m, 4H), 1.62–1.57 (m, 5H), 1.54–1.48 (m, 5H), 1.46 (s, 7H), 1.31 (t, *J* = 6.9 Hz, 6H), 1.26–1.14 (m, 6H), 1.05 (d, *J* = 5.8 Hz, 2H), 0.96 (d, *J* = 6.7 Hz, 3H), 0.90–0.85 (m, 9H); ^13^C NMR (CDCl_3_) δ 213.3, 209.1, 173.6, 161.2, 107.0, 97.1, 82.4, 67.9, 61.8, 57.4, 54.3, 47.5, 45.3, 41.4, 40.4, 35.6, 34.5, 33.7, 29.9, 29.6, 27.3, 25.8, 25.1, 23.7, 22.2, 20.5, 20.3, 18.8, 16.4, 12.6, 12.1; HRMS (ESI/Q-TOF): m/z 1119.6185 [M + Na]^+^; for the compound C_55_H_93_N_4_O_16_P requires 1119.6216.

*4-(N-(3-ammoniopropyl)-4-((3-(diethoxyphosphoryl)propyl)(hydroxy)amino)-4-oxobutanamido)butan-1-aminium 2,2,2-bis(trifluoroacetate)* (**24**): Yellow oil (61 mg, 91%); HRMS (ESI/Q-TOF): m/z 439.2687 [M + H]^+^; for the compound C_18_H_39_N_4_O_6_P requires 439.2680.

*4,4′-((4-((3-(diethoxyphosphoryl)propyl)(hydroxy)amino)-4-oxobutanoyl)azanediyl)bis(butan-1-aminium) 2,2,2-bis(trifluoroacetate)* (25): Yellow oil (65 mg, 95%); HRMS (ESI/Q-TOF): m/z 453.2848 [M + H]^+^; for the compound C_19_H_41_N_4_O_6_P requires 453.2836.

#### 3.2.4. Synthesis of FSM-Amino and/or Amido Derivatives **26**–**33**

General procedure for the synthesis of Fosmidomycin conjugates with amines. To a solution of **42** (1 mmol) in CH_2_Cl_2_ (1.4 mL), the corresponding amines (1 mmol), HBTU (1.1 mmol), and DIPEA (1.5 mmol) were added. The reaction mixture was stirred at ambient temperature to complete the reaction (monitored by TLC) and then diluted with CH_2_Cl_2_, washed with 5% aqueous NaHCO_3_, pre-cooled 5% aqueous citric acid, water, and brine. The organic layer was dried over Na_2_SO_4_, filtered, and evaporated to dryness under reduced pressure. The residues thus obtained were subjected to FCC, affording the corresponding pure conjugates **60**–**67**.

*Tert-butyl 2-(4-((benzyloxy)(3-(diethoxyphosphoryl)propyl)amino)-4-oxobutanoyl) hydrazine-1-carboxylate* (**60**): Reaction time: 5 h; Yellow oil (320 mg, 62%); R_f_ (CHCl_3_/MeOH 97:3): 0.28; ^1^H NMR (CDCl_3_) *δ* 7.76 (br s, 1H), 7.37 (s, 5H), 4.85 (s, 2H), 4.12–4.03 (m, 4H), 3.74–3.68 (m, 2H), 2.83–2.78 (m, 2H), 2.55–2.50 (m, 2H), 1.96–1.88 (m, 2H), 1.69 (br s, 2H), 1.48 (s, 1H), 1.46 (s, 8H), 1.30 (t, *J* = 7.1 Hz, 6H); ^13^C NMR (CDCl_3_) *δ* 155.3, 129.3, 129.0, 128.8, 81.5, 76.6, 61.7, 61.6, 28.6, 28.2, 28.1, 27.7, 23.4, 22.5, 20.2, 16.4; ESI-MS (30eV): *m*/*z* 554.32 [M + K]^+^, 538.45 [M + Na]^+^, 516.4 [M + H]^+^, 384.6 [M−C_5_H_11_N_2_O_2_]^+^.

*Diethyl(3-(4-((2-aminophenyl)amino)-N-(benzyloxy)-4-oxobutanamido) propyl) phosphonate* (**61**): Reaction time: 4 h; Yellow oil (388 mg, 79%); R_f_ (CHCl_3_/MeOH 98:2): 0.15; ^1^H NMR (CDCl_3_) *δ* 7.77 (br s, 1H), 7.38 (s, 5H), 7.21 (dd, *J* = 8.2, 1.3 Hz, 1H), 7.02 (td, *J* = 7.7, 1.4 Hz, 1H), 6.76–6.72 (m, 2H), 4.86 (s, 2H), 4.11–4.01 (m, 4H), 3.72 (t, *J* = 6.2 Hz, 2H), 2.89 (t, *J* = 6.0 Hz, 2H), 2.65 (t, *J* = 6.6 Hz, 2H), 1.97–1.89 (m, 2H), 1.74–1.67 (m, 2H), 1.28 (t, *J* = 7.1 Hz, 6H); ^13^C NMR (CDCl_3_) *δ* 171.2, 141.2, 129.2, 129.1, 128.8, 127.1, 125.8, 123.7, 118.8, 117.2, 76.5, 61.7, 61.6, 31.3, 28.5, 23.4, 22.3, 19.6, 16.5, 16.4; ESI-MS (30eV): *m*/*z* 983.25 [2M + H]^+^, 530.35 [M + K]^+^, 514.29 [M + Na]^+^, 492.37 [M + H]^+^, 384.3 [M−C_6_H_7_N_2_]^+^.

*Diethyl(3-(N-(benzyloxy)-4-(dibenzylamino)-4-oxobutanamido)propyl)phosphonate* (**62**): Reaction time: 5 h; Pale yellow (441 mg, 76%); R*f* (AcOEt): 0.16; ^1^H NMR (CDCl_3_) *δ* 7.43–7.40 (m, 2H), 7.39–7.34 (m, 5H), 7.33–7.27 (m, 4H), 7.22–7.19 (m, 4H), 4.94 (s, 2H), 4.61 (s, 2H), 4.51 (s, 2H), 4.12–4.02 (m, 4H), 3.74 (t, *J* = 6.7 Hz, 2H), 2.91–2.86 (m, 2H), 2.76 (t, *J* = 6.4 Hz, 2H), 1.99–1.90 (m, 2H), 1.79–1.70 (m, 2H), 1.28 (t, *J* = 7.1 Hz, 6H); ^13^C NMR (CDCl_3_) *δ* 172.4, 137.3, 136.5, 129.2, 128.9, 128.9, 128.7, 128.6, 128.1, 127.6, 127.3, 126.6, 76.5, 61.5, 49.9, 48.2, 27.8, 27.6, 23.5, 22.5, 20.3, 16.5, 16.4; ESI-MS (30eV): *m*/*z* 1183.64 [2M + Na]^+^, 619.25 [M + K]^+^, 603.22 [M + Na]^+^, 581.38 [M + H]^+^, 280.52 [M−C_14_H_23_NO_4_P]^+^.

*Tert-butyl(4-(4-(4-((benzyloxy)(3-(diethoxyphosphoryl)propyl)amino)-4-oxobutanamido)benzyl)phenyl) carbamate* (**63**): Reaction time: 3 h; Yellow oil (573 mg, 84%); R*f* (CHCl_3_/MeOH 98:2): 0.25; ^1^H NMR (CDCl_3_) *δ* 8.04 (s, 1H), 7.39 (t, *J* = 6.9 Hz, 2H), 7.36 (s, 4H), 7.25 (s, 2H), 7.24 (s, 1H), 7.07 (t, *J* = 8.7 Hz, 4H), 6.42 (s, 1H), 4.85 (s, 2H), 4.12–3.97 (m, 4H), 3.86 (s, 2H), 3.72 (s, 2H), 2.83 (s, 2H), 2.64–2.59 (m, 2H), 1.96–1.88 (m, 2H), 1.75–1.67 (m, 2H), 1.49 (s, 9H), 1.27 (t, *J* = 7.1 Hz, 6H); ESI-MS (30eV): *m*/*z* 1401.75 [2M + K]^+^, 1385.75 [2M + Na]^+^, 1363.84 [2M + 2H]^+^, 720.36 [M + K]^+^, 704.49 [M + Na]^+^, 682.39 [M + H]^+^.

*Diethyl (3-(4-(benzylamino)-N-(benzyloxy)-4-oxobutanamido)propyl) phosphonate* (**64**): Reaction time: 6 h; Yellow oil (363 mg 74%); R*f* (CHCl_3_/MeOH 95:5): 0.29; ^1^H NMR (CDCl_3_) *δ* 7.41–7.35 (m, 5H), 7.34–7.29 (m, 2H), 7.28–7.23 (m, 3H), 6.27 (s, 1H), 4.85 (s, 2H), 4.43 (d, *J* = 5.7 Hz, 2H), 4.11–4.01 (m, 4H), 3.70 (t, *J* = 6.6 Hz, 2H), 2.81 (t, *J* = 6.0 Hz, 2H), 2.52 (t, *J* = 6.5 Hz, 2H), 1.96–1.87 (m, 2H), 1.75–1.66 (m, 2H), 1.29 (t, *J* = 7.1 Hz, 6H); ^13^C NMR (CDCl_3_) *δ* 173.8, 172.0, 138.4, 134.3, 129.2, 129.0, 128.7, 128.6, 127.7, 127.4, 76.5, 61.6, 45.8, 43.6, 30.8, 28.1, 23.5, 22.5, 20.3, 16.5; ESI-MS (30eV): *m*/*z* 529.34 [M + K]^+^, 513.41 [M + Na]^+^, 491.49 [M + H]^+^, 384.36 [M−C_7_H_8_N]^+^.

*Diethyl (3-(N-(benzyloxy)-4-oxo-4-(4-tritylpiperazin-1-yl)butanamido)propyl) phosphonate* (**66**): Reaction time: 3 h; Yellow oil (60 mg, 70%); R_f_ (CHCl_3_/MeOH 99:1): 0.18; ^1^H NMR (CDCl_3_) *δ* 7.47 (br s, 5H), 7.39–7.32 (m, 5H), 7.30–7.24 (m, 7H), 7.19–7.14 (m, 3H), 4.88 (s, 2H), 4.10–4.02 (m, 4H), 3.68 (t, *J* = 6.5 Hz, 2H), 3.62 (s, 2H), 2.73 (s, 2H), 2.54 (t, *J* = 6.2 Hz, 2H), 2.17 (s, 4H), 1.96–1.87 (m, 2H), 1.75–1.65 (m, 4H), 1.28 (t, *J* = 7.1 Hz, 6H); ^13^C NMR (CDCl_3_) *δ* 170.2, 134.4, 129.2, 128.8, 128.6, 127.7, 126.3, 70.4, 61.6, 48.2, 47.7, 45.8, 42.2, 38.6, 30.9, 27.4, 23.5, 22.5, 20.3, 16.4; ESI-MS (30eV): *m/z* 1445.58 [2M + Na]^+^, 734.41 [M + Na]^+^, 243.41 [Trt]^+^.

*Diethyl (3-(N-(benzyloxy)-4-(4-methylpiperazin-1-yl)-4-oxobutanamido)propyl) phosphonate* (**67**): Reaction time: 2 h; Yellow oil (338 mg 70%); R_f_ (CHCl_3_/MeOH 9:1): 0.16; ^1^H NMR (CDCl_3_) *δ* 7.41–7.32 (m, 5H), 4.91 (s, 2H), 4.11–4.01 (m, 4H), 3.70 (t, *J* = 6.8 Hz, 2H), 3.67–3.62 (m, 2H), 3.56–3.52 (m, 2H), 2.79 (s, 2H), 2.62 (t, *J* = 6.5 Hz, 2H), 2.44 (br s, 2H), 2.40 (br s, 2H), 2.32 (s, 3H), 1.96–1.88 (m, 2H), 1.76–1.67 (m, 2H), 1.28 (t, *J* = 7.1 Hz, 6H); ^13^C NMR (CDCl_3_) *δ* 137.1, 128.8, 128.4, 128.0, 76.3, 61.5, 59.2, 59.1, 29.7, 24.0, 23.1, 20.3, 16.5; ESI-MS (30eV): *m*/*z* 522.33 [M + K]^+^, 506.39 [M + Na]^+^, 484.41 [M + H]^+^, 384.35 [M−C_5_H_11_N_2_]^+^.

*Trityl-deprotection of 4-(4-((benzyloxy)(3-(diethoxyphosphoryl) propyl)amino)-4-oxobutanoyl)piperazin- 1-ium2,2,2-trifluoroacetate* (**68**): To an ice-cold solution of **66** (0.18 mmol) in CH_2_Cl_2_ (0.56 mL), trifluroethanol (TFE) (0.36 mmol) and TFA (0.36 mmol) were added. The reaction mixture was stirred at ambient temperature for 1h. Volatile components were evaporated under vacuo, and the oily residue was triturated with Et_2_O and refrigerated overnight. The white precipitate was filtered under vacuum and dried to afford the trifluoroacetate salt **68**. White solid (74 mg, 70%); R_f_ (CHCl_3_/MeOH 9:1): 0.16; ^1^H NMR (MeOD): δ 7.50–7.45 (m, 2H), 7.44–7.38 (m, 3H), 4.98 (s, 2H), 4.13–4.02 (m, 4H), 3.86–3.75 (m, 6H), 3.21 (br s, 2H), 2.82 (br s, 2H), 2.72–2.67 (m, 2H), 1.95–1.87 (m, 2H), 1.84–1.78 (m, 2H), 1.31 (t, J = 7.1 Hz, 6H).

Deprotection of intermediates **60**−**65**, **67**, and **68**. A solution of **60**−**65**, **67**, and **68** (0.1 mmol) in methanol (3 mL) was subjected to hydrogenolysis over 10% Pd/C at ambient temperature and pressure until completion of the reaction. Thus, the reaction mixture was filtered through Celite and the filter cake was washed several times with methanol. After evaporation of the solvent to dryness under reduced pressure, the residues were subjected to FCC, affording the corresponding pure deprotected molecules **26**–**33**.

*Tert-butyl 2-(4-((3-(diethoxyphosphoryl)propyl)(hydroxy)amino)-4-oxobutanoyl) hydrazine-1-carboxylate* (**26**): Reaction time: 5 h; Orange oil (38 mg 90%); R_f_ (CHCl_3_/MeOH 95:): 0.19; ^1^H NMR (CDCl_3_) *δ* 9.58 (s, 1H), 8.52 (s, 1H), 7.05 (s, 1H), 4.13–4.03 (m, 4H), 3.70 (t, *J* = 6.0 Hz, 2H), 2.86 (t, *J* = 6.3 Hz, 2H), 2.58–2.52 (m, 2H), 1.96–1.87 (m, 2H), 1.78 (m, 2H), 1.47 (s, 1H), 1.45 (s, 8H), 1.31 (t, *J* = 7.0 Hz, 6H); ^13^C NMR (CDCl_3_) *δ* 173.3, 155.6, 81.5, 62.1, 47.9, 29.3, 28.2, 27.7, 22.8, 21.8, 19.4, 16.4; HRMS (ESI/Q-TOF): *m*/*z* 426.2000 [M + H]^+^; for the compound C_16_H_32_N_3_O_8_P requires 426.2005.

*Diethyl(3-(4-((2-aminophenyl)amino)-N-hydroxy-4-oxobutanamido) propyl) phosphonate* (**27**): Reaction time: 3 h; Yellow oil (54 mg, 89%); R_f_ (CHCl_3_/MeOH 95:5): 0.11; ^1^H NMR (CDCl_3_) *δ* 9.76 (br s, 1H), 8.20 (s, 1H), 7.20 (d, *J* = 7.9 Hz, 1H), 7.02–6.99 (m, 1H), 6.74–6.71 (m, 1H), 4.04–3.99 (m, 4H), 3.72 (t, *J* = 6.1 Hz, 2H), 2.96–2.92 (m, 2H), 2.70–2.66 (m, 2H), 1.97–1.89 (m, 2H), 1.78 (dt, *J* = 18.5, 6.9 Hz, 2H), 1.26 (t, *J* = 7.1 Hz, 6H); ^13^C NMR (CDCl_3_) *δ* 173.9, 172.0, 141.0, 127.0, 125.7, 123.8, 118.8, 117.1, 62.3, 62.2, 47.9, 31.9, 28.6, 22.8, 21.8, 19.2, 16.3; HRMS (ESI/Q-TOF): *m*/*z* 424.1615 [M + Na]^+^; for the compound C_17_H_28_N_3_O_6_P requires 424.1608.

*Diethyl (3-(4-(dibenzylamino)-N-hydroxy-4-oxobutanamido) propyl)phosphonate* (**28**): Reaction time: 4 h; Yellow oil (39 mg, 80%); R_f_ (CHCl_3_/MeOH 98:2): 0.21; ^1^H NMR (CDCl_3_) *δ* 9.86 (s, 1H), 7.39–7.34 (m, 2H), 7.33–7.27 (m, 4H), 7.21–7.13 (m, 4H), 4.59 (s, 2H), 4.49 (s, 2H), 4.14–4.02 (m, 4H), 3.75 (t, *J* = 6.0 Hz, 2H), 2.91–2.81 (m, 4H), 2.01–1.92 (m, 2H), 1.81 (dt, *J* = 18.2, 7.1 Hz, 2H), 1.28 (t, *J* = 7.0 Hz, 6H); ^13^C NMR (CDCl_3_) *δ* 173.3, 173.2, 136.9, 136.0, 129.0, 128.6, 128.1, 127.7, 127.4, 126.5, 61.9, 50.0, 48.5, 47.6, 47.5, 29.2, 27.3, 22.8, 21.9, 19.3, 16.4; HRMS (ESI/Q-TOF): *m*/*z* 491.2311 [M + H]^+^; for the compound C_25_H_35_N_2_O_6_P requires 491.2305.

*Tert-butyl(4-(4-(4-((3-(diethoxyphosphoryl)propyl)(hydroxy)amino)-4-oxobutanamido)benzyl)phenyl) carbamate* (**29**): Reaction time: 3 h; light yellow oil (53 mg, 90%); R_f_ (CHCl_3_/MeOH 97:3): 0.09; ^1^H NMR (CDCl_3_) *δ* 9.78 (br s, 1H), 8.70 (s, 1H), 7.40 (d, *J* = 8.3 Hz, 2H), 7.24 (d, *J* = 7.8 Hz, 2H), 7.07–7.03 (m, 4H), 6.48 (s, 1H), 4.00 (quint, *J* = 14.4, 7.0 Hz, 4H), 3.85 (s, 2H), 3.74 (t, *J* = 6.0 Hz, 2H), 2.94–2.88 (m, 2H), 2.70–2.62 (m, 2H), 1.99–1.90 (m, 3H), 1.82–1.74 (m, 2H), 1.50 (s, 9H), 1.23 (t, *J* = 7.1 Hz, 6H); ^13^C NMR (CDCl_3_) *δ* 173.9, 171.3, 152.9, 136.9, 136.4, 135.9, 129.3, 129.2, 129.0, 128.2, 125.3, 119.9, 118.8, 62.3, 47.9, 40.6, 32.6, 28.4, 22.7, 21.8, 19.1, 16.3; HRMS (ESI/Q-TOF): *m/z* 592.2787 [M + H]^+^; for the compound C_29_H_42_N_3_O_8_P requires 592.2782.

*Diethyl (3-(4-(benzylamino)-N-hydroxy-4-oxobutanamido)propyl) phosphonate* (**30**): Reaction time: 3 h; Reddish oil (36 mg, 90%); R_f_ (CHCl_3_/MeOH 95:5): 0.09; ^1^H NMR (CDCl_3_) *δ* 9.83 (br s, 1H), 7.32–7.27 (m, 2H), 7.25–7.21 (m, 3H), 6.78 (s, 1H), 4.38 (s, 2H), 4.09–4.00 (m, 4H), 3.71–3.65 (m, 2H), 2.84 (s, 2H), 2.56 (s, 2H), 1.97–1.87 (m, 2H), 1.81–1.71 (m, 2H), 1.29 (t, *J* = 7.0 Hz, 6H); ^13^C NMR (CDCl_3_) *δ* 173.5, 172.9, 138.3, 128.6, 127.6, 127.3, 62.1, 47.8, 47.7, 43.6, 31.4, 27.9, 22.8, 21.9, 19.4, 16.4; HRMS (ESI/Q-TOF): *m*/*z* 423.1657 [M + Na]^+^; for the compound C_18_H_29_N_2_O_6_P requires 423.1655.

*Diethyl(3-(4-(benzhydrylamino)-N-hydroxy-4-oxobutanamido)propyl)phosphonate* (**31**): Reaction time: 3 h; Reddish oil (40 mg, 85%); R_f_ (PhMe/AcOEt 4:6): 0.1; ^1^H NMR (CDCl_3_) *δ* 7.40–7.09 (m, 10), 6.25–6.11 (m, 1H), 4.17–3.95 (m, 4H), 3.76–3.61 (m, 2H), 2.84 (s, 2H), 2.59 (s, 2H), 2.07–1.85 (m, 2H), 1.84–1.65 (m, 2H), 1.34–1.21 (m, 6H); ^13^C NMR (CDCl_3_) *δ* 173.7, 172.2, 141.7, 128.6, 127.4, 127.3, 62.1, 56.0, 50.8, 47.3, 31.5, 28.8, 28.0, 21.5, 19.3, 16.4; HRMS (ESI/Q-TOF): *m*/*z* 499.1979 [M + Na]^+^; for the compound C_24_H_33_N_2_O_6_P requires 499.1968.

*Diethyl (3-(N-hydroxy-4-(4-methylpiperazin-1-yl)-4-oxobutanamido) propyl) phosphonate* (**32**): Reaction time: 2 h; Orange oil (35 mg, 90%); R_f_ (CHCl_3_/MeOH 8:2): 0.29; ^1^H NMR (CDCl_3_) *δ* 4.16–4.01 (m, 7H), 3.70 (t, *J* = 6.9 Hz, 1H), 3.62 (br s, 2H), 3.52 (t, *J* = 5.2 Hz, 2H), 2.79 (t, *J* = 6.8 Hz, 1H), 2.70 (t, *J* = 7.0 Hz, 1H), 2.65 (quint, *J* = 10.8, 4.5 Hz, 1H), 2.45 (br s, 1H), 2.40 (br s, 1H), 2.32 (s, 3H), 1.97–1.90 (m, 2H), 1.80–1.73 (m, 2H), 1.31 (t, *J* = 7.0 Hz, 6H); ^13^C NMR (CDCl_3_) *δ* 173.7, 173.2, 62.2, 51.7, 47.8, 29.0, 28.7, 27.5, 22.5, 21.6, 19.0, 16.4; HRMS (ESI/Q-TOF): *m/z* 416.1848 [M + Na]^+^; for the compound C_16_H_32_N_3_O_6_P requires 416.1921.

*4-(4-((3-(Diethoxyphosphoryl)propyl)(hydroxy)amino)-4-oxobutanoyl)piperazin-1-ium 2,2,2-trifluoroacetate* (**33**): Reaction time: 4 h; Yellow oil (30 mg, 60%); R_f_ (CHCl_3_/MeOH 8:2): 0.22; ^1^H NMR (CDCl_3_) *δ* 9.84 (br s, 1H), 4.13–4.02 (m, 4H), 3.82 (br s, 2H), 3.74–3.66 (m, 2H), 3.24–3.10 (m, 2H), 2.84 (s, 2H), 2.68–2.62 (m, 2H), 2.55 (s, 1H), 1.98–1.88 (m, 2H), 1.78 (dt, *J* = 18.0, 6.9 Hz, 2H), 1.34–1.29 (m, 6H); ^13^C NMR (CDCl_3_) *δ* 173.2, 171.3, 62.1, 53.7, 48.5, 43.4, 29.7, 27.4, 22.7, 21.7, 19.4, 16.4; HRMS (ESI/Q-TOF): *m*/*z* 380.1948 [M + H]^+^; for the compound C_15_H_30_N_3_O_6_P requires 380.1945.

Diethyl (3-((2-aminophenyl)amino)propyl)phosphonate (**34**): To a solution of bromide **37** (90 mg, 0.35 mmol) and *ortho*-benzenediamine (35 mg, 0.35 mmol) in dimethylformamide (DMF) (0.46 mL), 1,8-diazabicyclo[5.4.0]undec-7-ene (DBU) (106 mg, 0.7 mmol) was added and the reaction mixture was subjected to ultrasonic radiation at 40 °C for 1 h. Upon completion of the reaction, the mixture was diluted CH_2_Cl_2_, and washed three times with water and once with brine. The organic layer was dried over Na_2_SO_4_ and evaporated to dryness under pressure. The residue was purified by FCC, affording compound **34**; orange oil (33 mg, 33%); R_f_ (CHCl_3_/MeOH 97:3): 0.3; ^1^H NMR (CDCl_3_): *δ* 6.79 (dt, *J* = 7.5, 1.6 Hz, 1H), 6.70 (dd, *J* = 7.7, 1.9 Hz, 1H), 6.68–6.60 (m, 2H), 4.15–4.04 (m, 4H), 3.20 (t, *J* = 6.6 Hz, 2H), 2.01–1.92 (m, 2H), 1.921–1.84 (m, 2H), 1.31 (t, *J* = 7.0 Hz, 6H); ^13^C NMR (CDCl_3_) *δ* 137.4, 134.3, 120.5, 118.6, 116.4, 111.6, 61.6, 44.4, 44.3, 23.9, 23.0, 22.5, 22.5, 16.5; HRMS (ESI/Q-TOF): *m*/*z* 287.1525 [M + H]^+^; for the compound C_13_H_23_N_2_O_3_P requires 287.1519.

(3-((2-Aminophenyl)amino)propyl)phosphonic acid (**35**): To an ice-cold solution of **34** (30 mg, 0.1 mmol) in CH_2_Cl_2_ (0.33 mL), bromotrimethylsilane (TMSBr) (0.16 mL, 1.2 mmol) was added dropwise and the reaction mixture was stirred at room temperature for 8 h. Then, it was concentrated under vacuum and treated with a solution of NaOH (0.41 mg, 0.1 mmol) in MeOH/H_2_O 9:1 (1 mL) at room temperature for 40 min The monosodium salt **35** was received after evaporation of the solvents to dryness under reduced pressure as an orange oil (19 mg, 75%); ^1^H NMR (H_2_D): *δ* 7.18–7.09 (m, 3H), 7.08–7.04 (m, 1H), 3.32 (t, *J* = 7.4 Hz, 2H), 1.92–1.84 (m, 2H), 1.82–1.75 (m, 2H); ^13^C NMR (H_2_D) *δ* 131.8, 126.7, 125.4, 122.9, 119.8, 47.7, 24.3, 23.4, 20.5; ESI-MS (30eV): *m*/*z* 499.34 [2M + K]^+^, 483.42 [2M + Na]^+^, 461.45 [2M + H]^+^, 269.48 [M + K]^+^, 253.5 [M + Na]^+^, 231.53 [M + H]^+^.

### 3.3. Biological Evaluation

#### 3.3.1. Biological Assays

Cytotoxicity evaluation was performed upon human primary fibroblasts (cell line AB943) [35]. Assays were realized in 96-well plates in DMEM + Glutamax without phenol red medium (Gibco) containing 25 mM HEPES, pH 7.3, 10% fetal calf serum under a 5% CO_2_ atmosphere, at 37 °C. After trypsin treatment, AB943 cells were seeded at 2000 cells per well in 100 µL. After 24 h of incubation, drugs diluted in culture medium were added (100 µL per well). Drug stock solutions were prepared in dimethyl sulfoxide (DMSO). The final DMSO concentration in the cultures remained below 1%. Control cultures were constituted of cultures treated with DMSO instead of drug. The cytotoxicity assay was based on the conversion of a redo-sensitive dye (resazurin) to a fluorescent product by viable cells. After 72 h of incubation, resazurin solution was added in each well at a final concentration of 45 µM. Fluorescence was measured at 530 nm excitation and 590 nm emission wavelengths after 4 h of incubation. The percentage of inhibition of cell growth was calculated by comparing the fluorescence of cells maintained in the presence of drug to that in the absence of drug. The concentration causing 50% growth inhibition (IC_50_) was obtained from the drug concentration–response curve and the results were expressed as the mean values ± standard deviations determined from several independent experiments.

#### 3.3.2. In Vitro Growth Inhibition of *P. falciparum*

The chloroquine-resistant FcB1/Colombia strain of *Plasmodium falciparum* was maintained in vitro on human erythrocytes in RPMI 1640 medium supplemented by 8% (*v*/*v*) heat-inactivated human serum, at 37 °C, under an atmosphere of 3% CO_2_, 6% O_2_, and 91% N_2_ [45]. In vitro drug susceptibility assays were measured by [^3^H]-hypoxanthine incorporation. Drug stock solutions were prepared in DMSO. Compounds were serially diluted two-fold with 100 μL of culture medium in 96-well plates. Asynchronous parasite cultures (100 μL, 1% parasitaemia and 1% final hematocrite) were then added to each well and incubated for 24 h at 37 °C prior to the addition of 0.5μCi of [^3^H]-hypoxanthine (GE Healthcare, France, 1 to 5Ci·mmol/mL) per well [42]. After a further incubation of 24 h, plates were frozen and thawed. Cell lysates were then collected onto fiberglass filters and counted in a liquid scintillation spectrometer. The growth inhibition for each drug concentration was determined by comparison of the radioactivity incorporated in the treated culture with that in the control culture maintained on the same plate. The IC_50_ value was obtained from the drug concentration–response curve and the results were expressed as the mean values ± standard deviations determined from several independent experiments. Chloroquine and artemisinin were used as antimalarial drug controls.

## Figures and Tables

**Table 1:** Evaluation of antiplasmodial activities of synthesized compounds with CQ-resistant *P. falciparum* FcB1/Colombia strain and selectivity indexes (SI).

Compound	MW	ClogP ^1^	IC_50_ (μM)	SI ^2^
FSM	205.08	−2.22	15.0 ± 1.7	>6.6
**17**	253.23	−0.43	>100	-
FR900098	219.11	−2.24	7.0 ± 0.0	>14.2
**18**	504.45	−1.22	>100	-
**19**	540.98	1.42	2.7 ± 0.5	23.9
**20**	628.97	2.24	4.2 ± 0.9	23.7
**21**	533.60	3.22	28.0 ± 11.6	-
**22**	1083.31	4.97	0.36 ± 0.09	94.1
**23**	1097.33	5.33	0.65 ± 0.26	34.0
**24**	667.56	−4.62	>100	-
**25**	680.58	−4.26	46.2 ± 8.0	-
**26**	425.42	−0.35	86.0 ± 1.4	-
**27**	401.40	−0.37	81.0 ± 2.8	-
**28**	490.54	1.92	38.7 ± 10.0	-
**29**	591.64	2.80	22.7 ± 1.9	1.7
**30**	400.41	0.25	>100	-
**31**	476.51	1.94	44.2 ± 9.5	-
**32**	393.42	−1.25	>100	-
**33**	493.42	−3.09	>100	-
**34**	286.31	1.56	8.2 ± 2.7	3.7
**35**	230.20	−0.24	>100	-
ART	282.34	2.90	55.0 ± 13.6 (nM)	>4545
CQ	318.89	5.70	72.0 ± 7.4 (nM)	347

^1^ XLogGP3 values calculated with the web tool SwissADME [44]. ^2^ SI = IC_50_ (AB943)/IC_50_ (*P.f.* FcB1)

## Supplementary Materials

The following are available online, Figure S1: ^1^H-NMR spectrum of compound 42, Figure S2: ^13^C-NMR spectrum of compound 42, Figure S3: ^31^P-NMR spectrum of compound 42, Figure S4: ^1^H-NMR spectrum of compound 43, Figure S5: ^13^C-NMR spectrum of compound 43, Figure S6: ^1^H-NMR spectrum of compound 18, Figure S7: ^13^C-NMR spectrum of compound 18, Figure S8: ^1^H-NMR spectrum of compound 47, Figure S9: ^13^C-NMR spectrum of compound 47, Figure S10: ^1^H-NMR spectrum of compound 19, Figure S11: ^13^C-NMR spectrum of compound 19, Figure S12: ^1^H-NMR spectrum of compound 20, Figure S13: ^13^C-NMR spectrum of compound 20, Figure S14: ^1^H-NMR spectrum of compound 21, Figure S15: ^13^C-NMR spectrum of compound 21, Figure S16: ^1^H-NMR spectrum of compound 54, Figure S17: ^13^C-NMR spectrum of compound 54, Figure S18: ^1^H-NMR spectrum of compound 55, Figure S19: ^13^C-NMR spectrum of compound 55, Figure S20: ^1^H-NMR spectrum of compound 56, Figure S21: ^13^C-NMR spectrum of compound 56, Figure S22: ^1^H-NMR spectrum of compound 57, Figure S23: ^13^C-NMR spectrum of compound 57, Figure S24: ^1^H-NMR spectrum of compound 22, Figure S25: ^13^C-NMR spectrum of compound 22, Figure S26: ^1^H-NMR spectrum of compound 23, Figure S27: ^13^C-NMR spectrum of compound 23, Figure S28: ^1^H-NMR spectrum of compound 60, Figure S29: ^13^C-NMR spectrum of compound 60, Figure S30: ^1^H-NMR spectrum of compound 61, Figure S31: ^13^C-NMR spectrum of compound 61, Figure S32: ^1^H-NMR spectrum of compound 62, Figure S33: ^13^C-NMR spectrum of compound 62, Figure S34: ^1^H-NMR spectrum of compound 63, Figure S35: ^1^H-NMR spectrum of compound 64, Figure S36: ^13^C-NMR spectrum of compound 64, Figure S37: ^1^H-NMR spectrum of compound 66, Figure S38: ^13^C-NMR spectrum of compound 66, Figure S39: ^1^H-NMR spectrum of compound 67, Figure S40: ^13^C-NMR spectrum of compound 67, Figure S41: ^1^H-NMR spectrum of compound 68, Figure S42: ^1^H-NMR spectrum of compound 26, Figure S43: ^13^C-NMR spectrum of compound 26, Figure S44: ^1^H-NMR spectrum of compound 27, Figure S45: ^13^C-NMR spectrum of compound 27, Figure S46: ^1^H-NMR spectrum of compound 28, Figure S47: ^13^C-NMR spectrum of compound 28, Figure S48: ^1^H-NMR spectrum of compound 29, Figure S49: ^13^C-NMR spectrum of compound 29, Figure S50: ^1^H-NMR spectrum of compound 30, Figure S51: ^13^C-NMR spectrum of compound 30, Figure S52: ^1^H-NMR spectrum of compound 31, Figure S53: ^13^C-NMR spectrum of compound 31, Figure S54: ^13^C-NMR spectrum of compound 32, Figure S55: ^13^C-NMR spectrum of compound 32, Figure S56: ^1^H-NMR spectrum of compound 33, Figure S57: ^1^H-NMR spectrum of compound 33, Figure S58: ^1^H-NMR spectrum of compound 34, Figure S59: ^13^C-NMR spectrum of compound 34, Figure S60: ^1^H-NMR spectrum of compound 35, Figure S61: ^13^C-NMR spectrum of compound 35.
