# Supplementary material for: Synthesis and Antiplasmodial Activity of Novel Fosmidomycin Derivatives and Conjugates with Artemisinin and Aminochloroquinoline
Source: Molecules. 2020 Oct 21;25(20):4858. doi: 10.3390/molecules25204858 (PMC7587979; doi:10.3390/molecules25204858)
Supplement: Supplementary file 1 [file molecules-25-04858-s001.pdf]

SUPPORTING INFORMATION

Synthesis and antiplasmodial activity of novel Fosmidomycin derivatives and conjugates with Artemisinin and Aminochloroquinoline

Despina Palla [1], Antonia I. Antoniou [1], Michel Baltas [2,3], Christophe Menendez [2,3], Philippe Grelier[3], Elisabeth Mouray [4] and Constantinos M. Athanassopoulos [1,*]

[1] Synthetic Organic Chemistry Laboratory, Department of Chemistry,
University of Patras, Patras GR-26504, Greece;

despina.pal.5@gmail.com (D.P.); tonadoniou@upatras.gr (A.I.A.); kath@chemistry.upatras.gr (C.M.A.)

[2] LSPCMIB, UMR-CNRS 5068, Université Paul Sabatier-Toulouse III, Toulouse CEDEX 9 31062, France; baltas@chimie.ups-tlse.fr (M.B.); menendez@chimie.ups-tlse.fr (C.M.)

[3] CNRS, LCC (Laboratire de Chimie, de Coordination), Université de Tolouse, UPS, INPT, 205 route de Narbonne, BP 44099, F-31077, Toulouse CEDEX 4, France;

michel.baltas@lcc-toulouse.fr (M.B); menendez@chimie.ups-tlse.fr (C.M.)

[4] MCAM,UMR 7245, Muséum National d'Histoire Naturelle, CNRS, CP52, 63 rue Buffon Paris 75005, France; grellier@mnhn.fr (P.G.); mouray@mnhn.fr (E.M.)

* Correspondence: kath@chemistry.upatras.gr; Tel.: +30-2610-997909

Copies of NMR spectra

Table of contents

Figure S1. ^{1}H-NMR spectrum of compound 42. 5
Figure S2. ^{13}C-NMR spectrum of compound 42. 5
Figure S3. ^{31}P-NMR spectrum of compound 42. 6
Figure S4. ^{1}H-NMR spectrum of compound 43. 6
Figure S5. ^{13}C-NMR spectrum of compound 43. 7
Figure S6. ^{1}H-NMR spectrum of compound 18. 7
Figure S7. ^{13}C-NMR spectrum of compound 18. 8
Figure S8. ^{1}H-NMR spectrum of compound 47. 8
Figure S9. ^{13}C-NMR spectrum of compound 47. 9
Figure S10. ^{1}H-NMR spectrum of compound 19. 9
Figure S11. ^{13}C-NMR spectrum of compound 19. 10
Figure S12. ^{1}H-NMR spectrum of compound 20. 10
Figure S13. ^{13}C-NMR spectrum of compound 20. 11
Figure S14. ^{1}H-NMR spectrum of compound 21. 11
Figure S15. ^{13}C-NMR spectrum of compound 21. 12
Figure S16. ^{1}H-NMR spectrum of compound 54. 12
Figure S17. ^{13}C-NMR spectrum of compound 54. 13
Figure S18. ^{1}H-NMR spectrum of compound 55. 13
Figure S19. ^{13}C-NMR spectrum of compound 55. 14
Figure S20. ^{1}H-NMR spectrum of compound 56. 14
Figure S21. ^{13}C-NMR spectrum of compound 56. 15
Figure S22. ^{1}H-NMR spectrum of compound 57. 15
Figure S23. ^{13}C-NMR spectrum of compound 57. 16
Figure S24. ^{1}H-NMR spectrum of compound 22. 16
Figure S25. ^{13}C-NMR spectrum of compound 22. 17
Figure S26. ^{1}H-NMR spectrum of compound 23. 17
Figure S27. ^{13}C-NMR spectrum of compound 23. 18

Figure S28. ^{1}H-NMR spectrum of compound 60. 18
Figure S29. ^{13}C-NMR spectrum of compound 60. 19
Figure S30. ^{1}H-NMR spectrum of compound 61. 19
Figure S31. ^{13}C-NMR spectrum of compound 61. 20
Figure S32. ^{1}H-NMR spectrum of compound 62. 20
Figure S33. ^{13}C-NMR spectrum of compound 62. 21
Figure S34. ^{1}H-NMR spectrum of compound 63. 21
Figure S35. ^{1}H-NMR spectrum of compound 64. 22
Figure S36. ^{13}C-NMR spectrum of compound 64. 22
Figure S37. ^{1}H-NMR spectrum of compound 66. 23
Figure S38. ^{13}C-NMR spectrum of compound 66. 23
Figure S39. ^{1}H-NMR spectrum of compound 67. 24
Figure S40. ^{13}C-NMR spectrum of compound 67. 24
Figure S41. ^{1}H-NMR spectrum of compound 68. 25
Figure S42. ^{1}H-NMR spectrum of compound 26. 26
Figure S43. ^{13}C-NMR spectrum of compound 26. 26
Figure S44. ^{1}H-NMR spectrum of compound 27. 27
Figure S45. ^{13}C-NMR spectrum of compound 27. 27
Figure S46. ^{1}H-NMR spectrum of compound 28. 28
Figure S47. ^{13}C-NMR spectrum of compound 28. 28
Figure S48. ^{1}H-NMR spectrum of compound 29. 29
Figure S49. ^{13}C-NMR spectrum of compound 29. 29
Figure S50. ^{1}H-NMR spectrum of compound 30. 30
Figure S51. ^{13}C-NMR spectrum of compound 30. 30
Figure S52. ^{1}H-NMR spectrum of compound 31. 31
Figure S53. ^{13}C-NMR spectrum of compound 31. 31
Figure S54. ^{13}C-NMR spectrum of compound 32. 32
Figure S55. ^{13}C-NMR spectrum of compound 32. 32
Figure S56. ^{1}H-NMR spectrum of compound 33. 33
Figure S57. ^{1}H-NMR spectrum of compound 33. 33
Figure S58. ^{1}H-NMR spectrum of compound 34. 34

Figure S59. ^{13}C-NMR spectrum of compound 34. 34
Figure S60. ^{1}H-NMR spectrum of compound 35. 35
Figure S61. ^{13}C-NMR spectrum of compound 35. 35

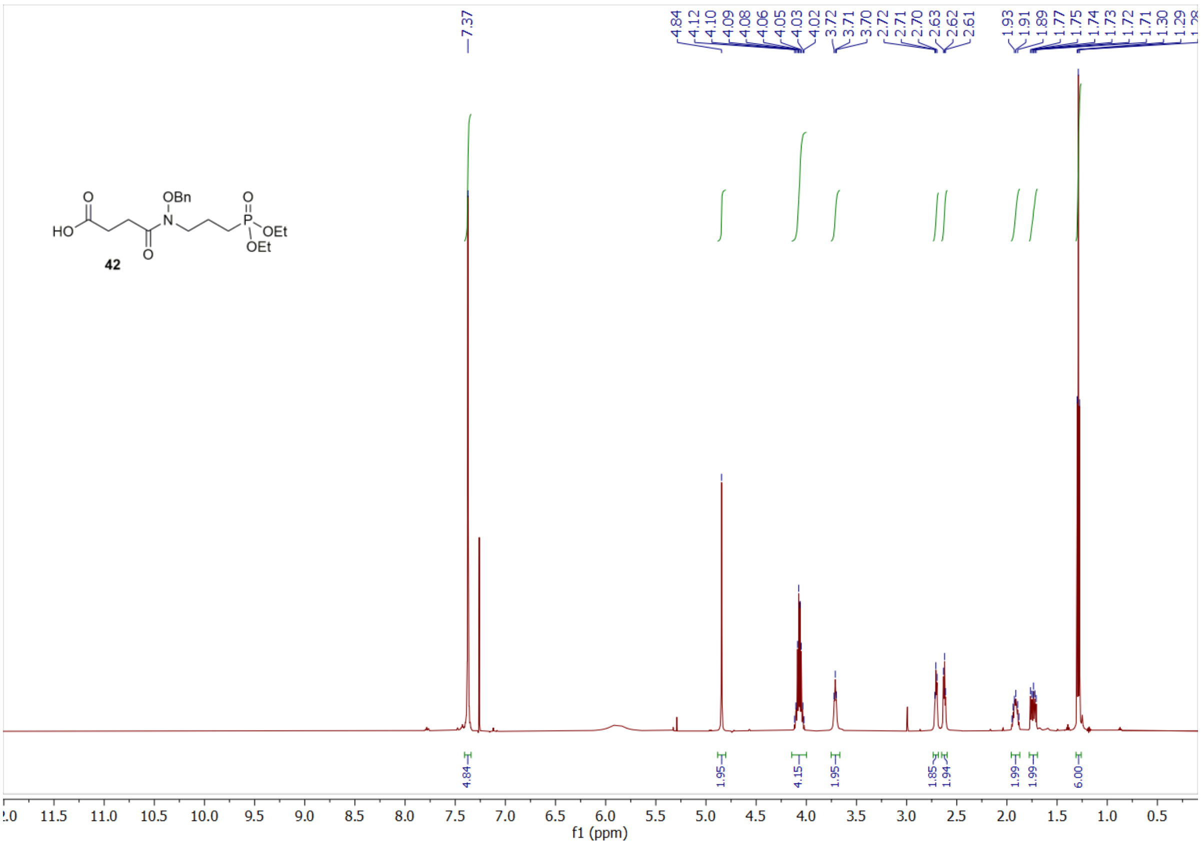

Figure S1. ¹H-NMR spectrum of compound 42.

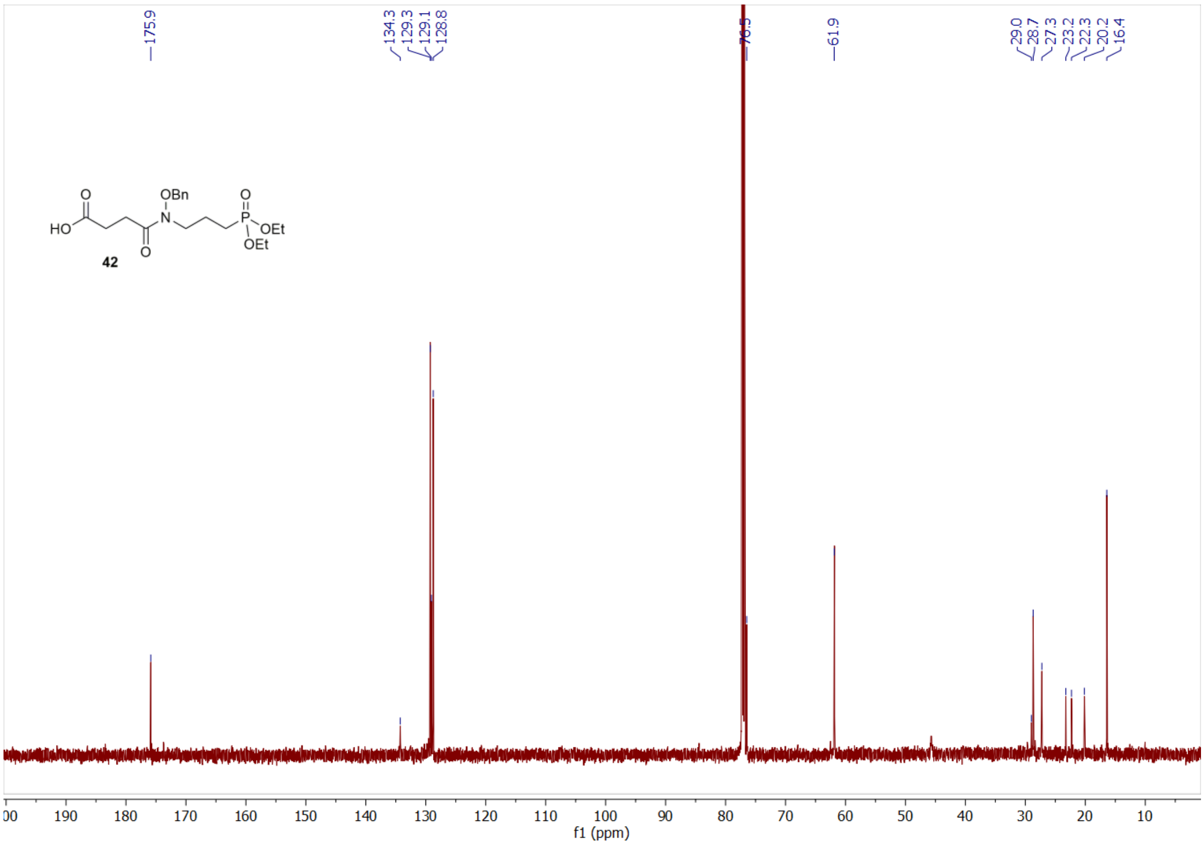

Figure S2. ¹³C-NMR spectrum of compound 42.

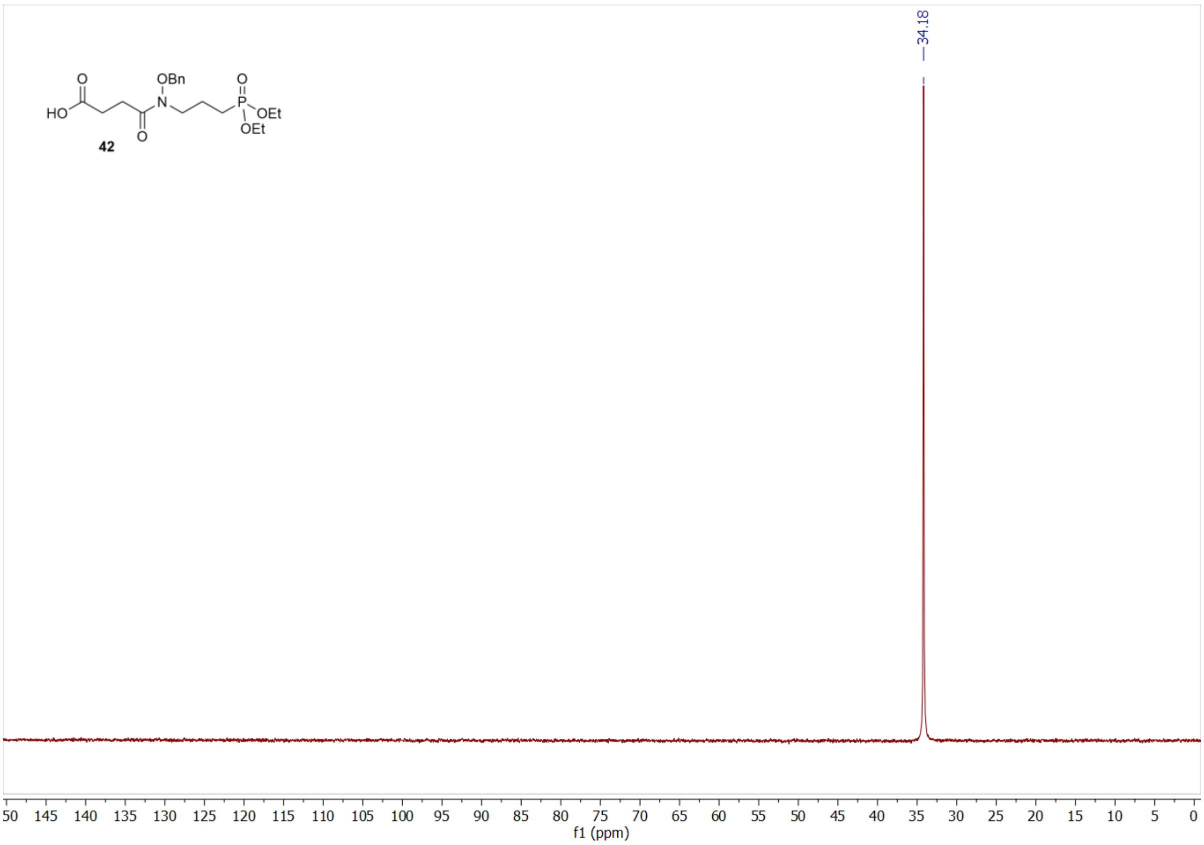

Figure S3. **^{31}P-NMR spectrum of compound 42.**

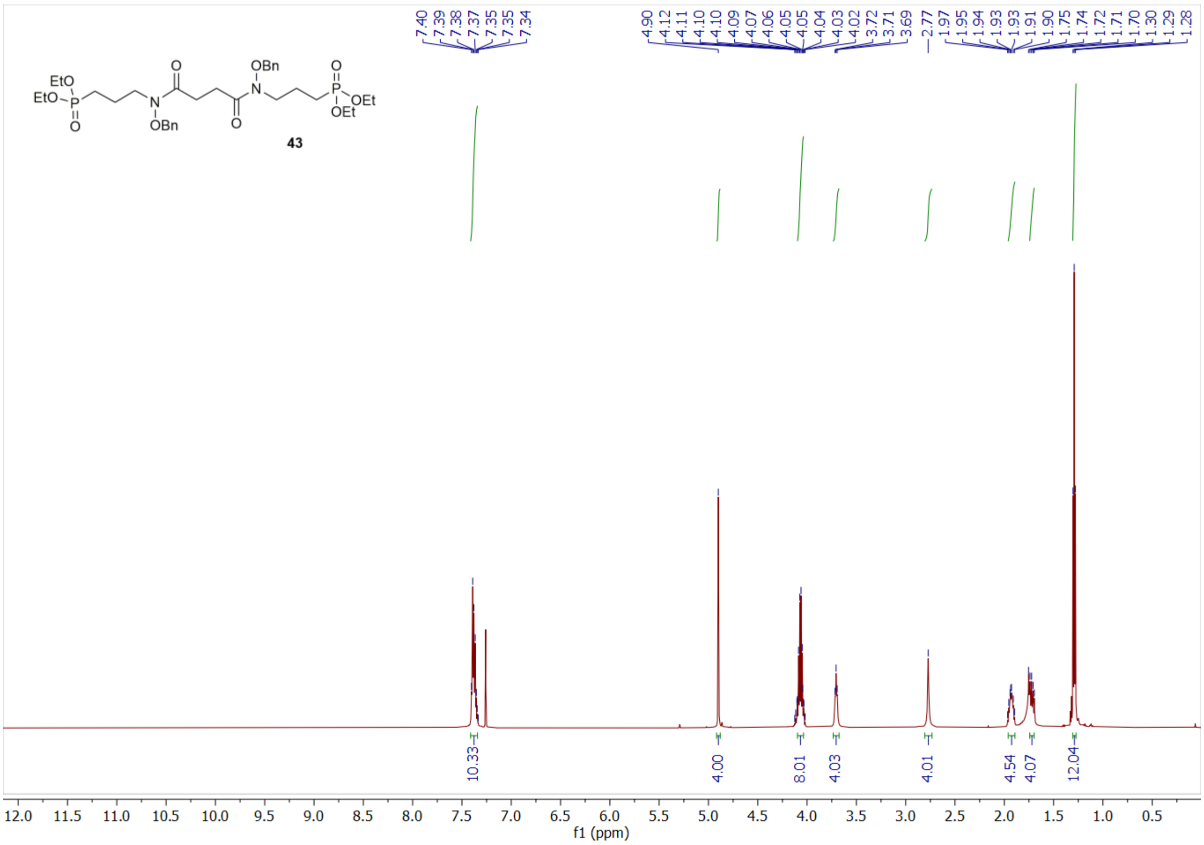

Figure S4. **^{1}H-NMR spectrum of compound 43.**

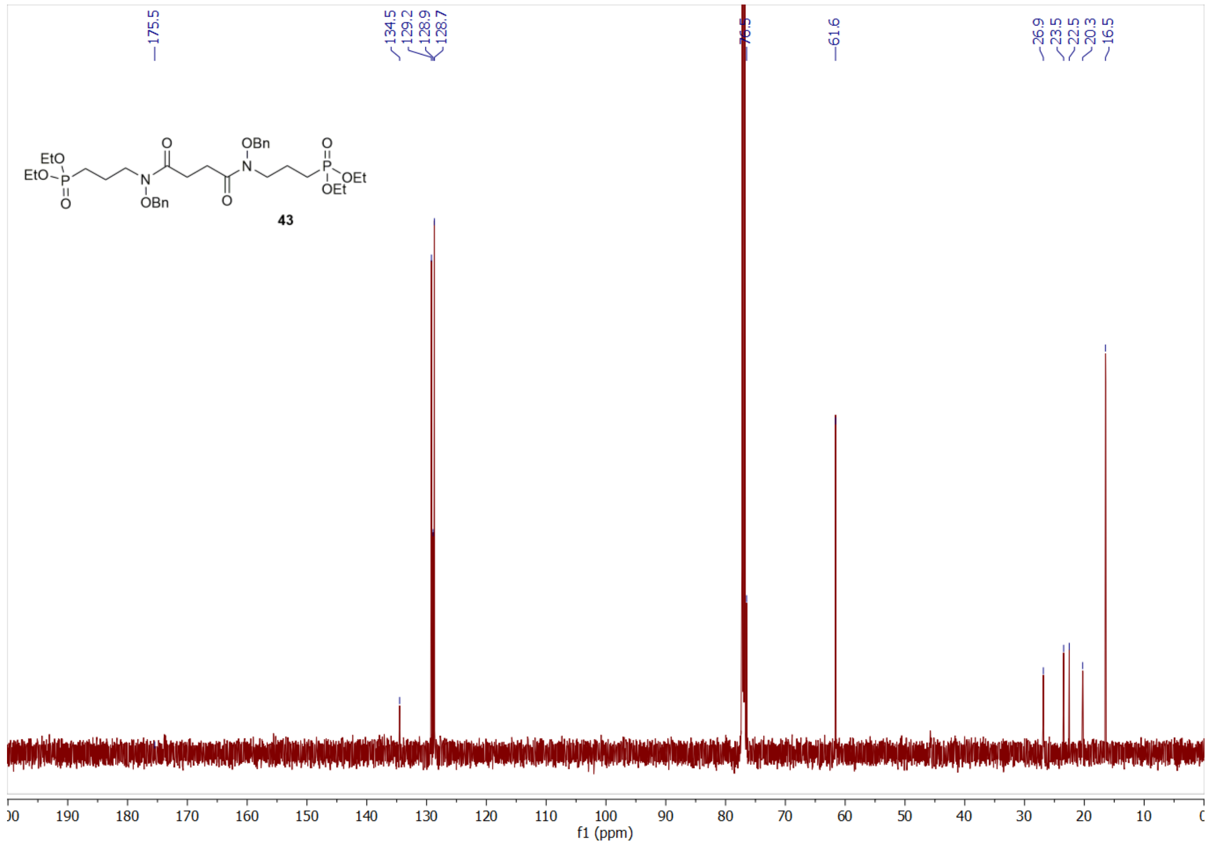

Figure S5. ^{13}**C-NMR spectrum of compound 43.**

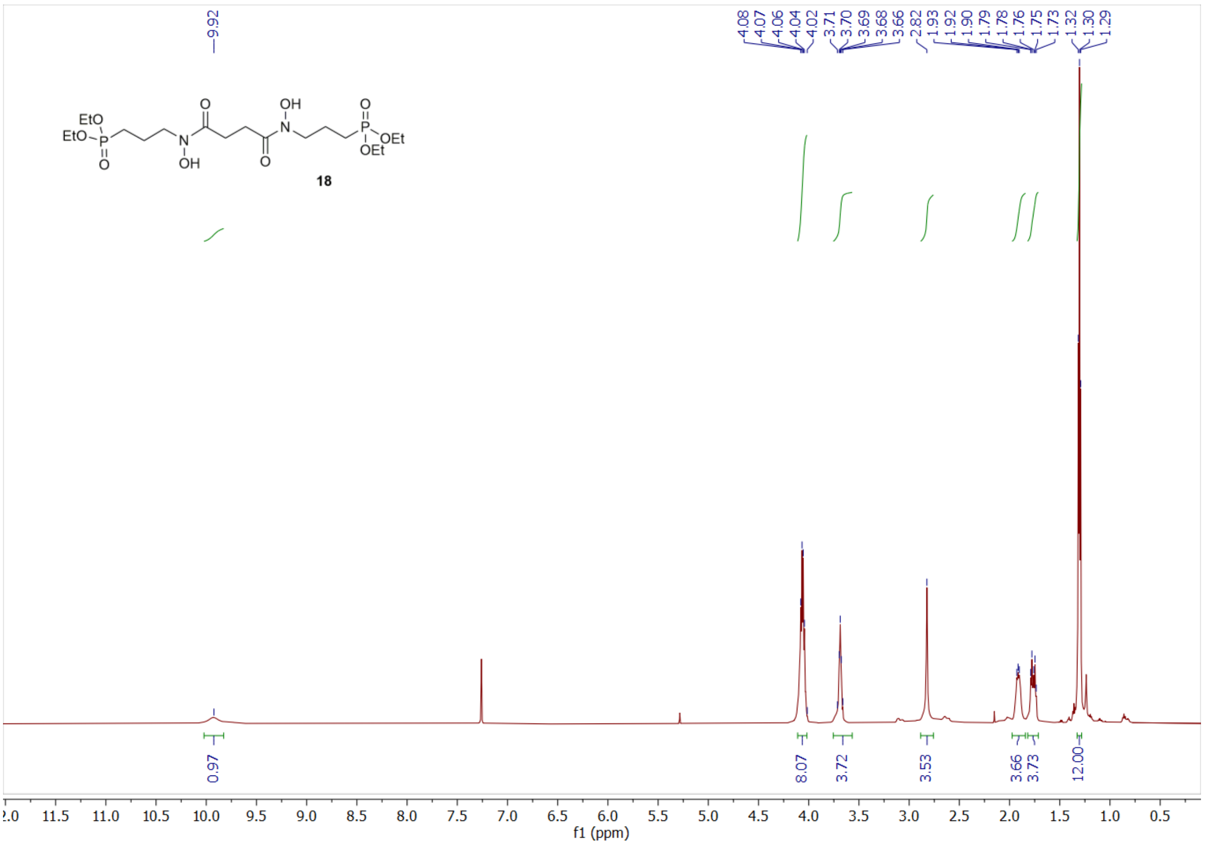

Figure S6. ^{1}**H-NMR spectrum of compound 18.**

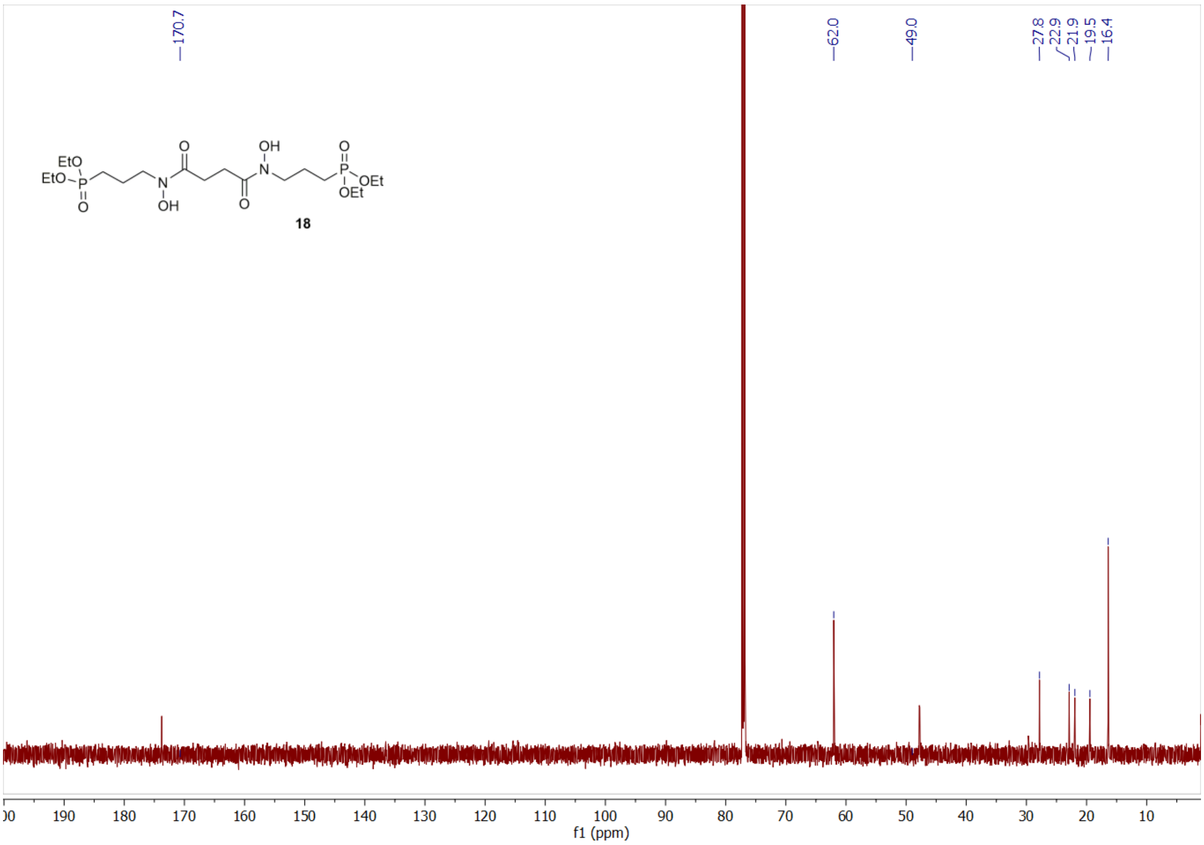

Figure S7. ^{13}C-NMR spectrum of compound 18.

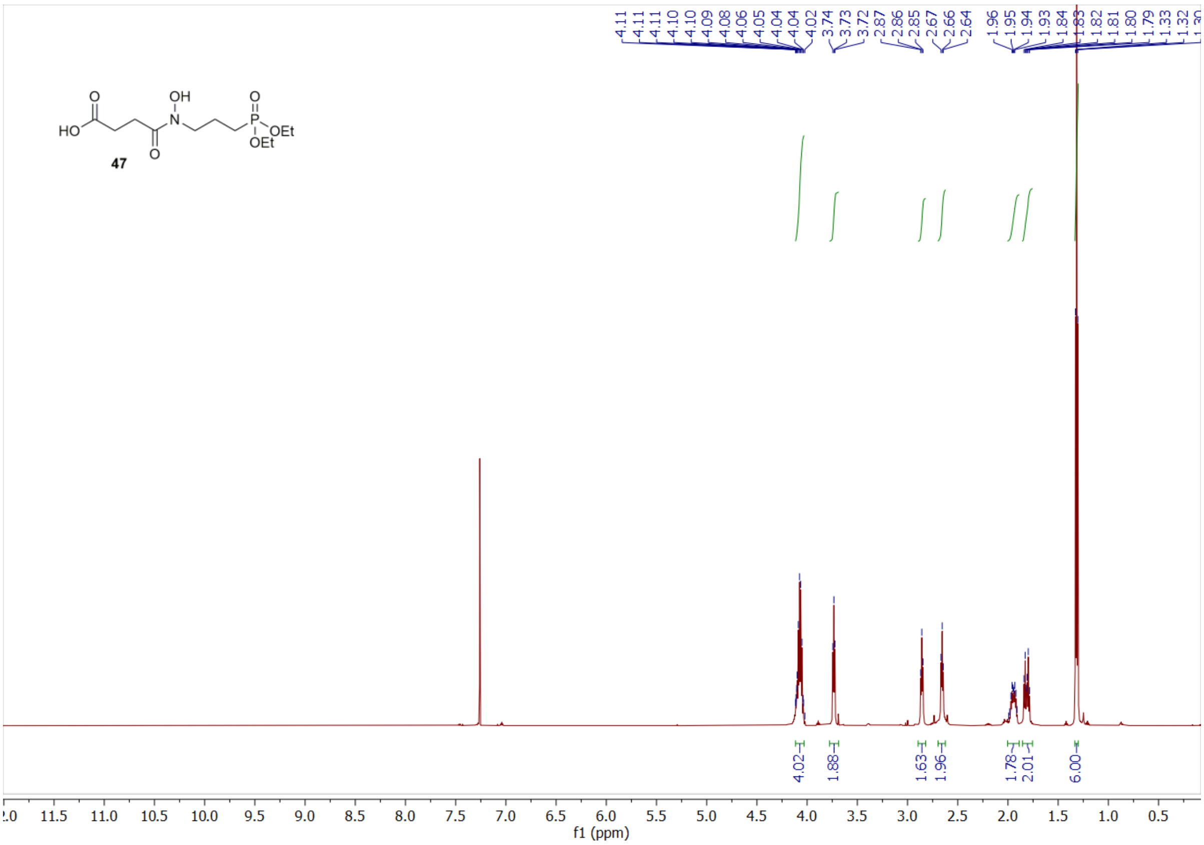

Figure S8. ^{1}H-NMR spectrum of compound 47.

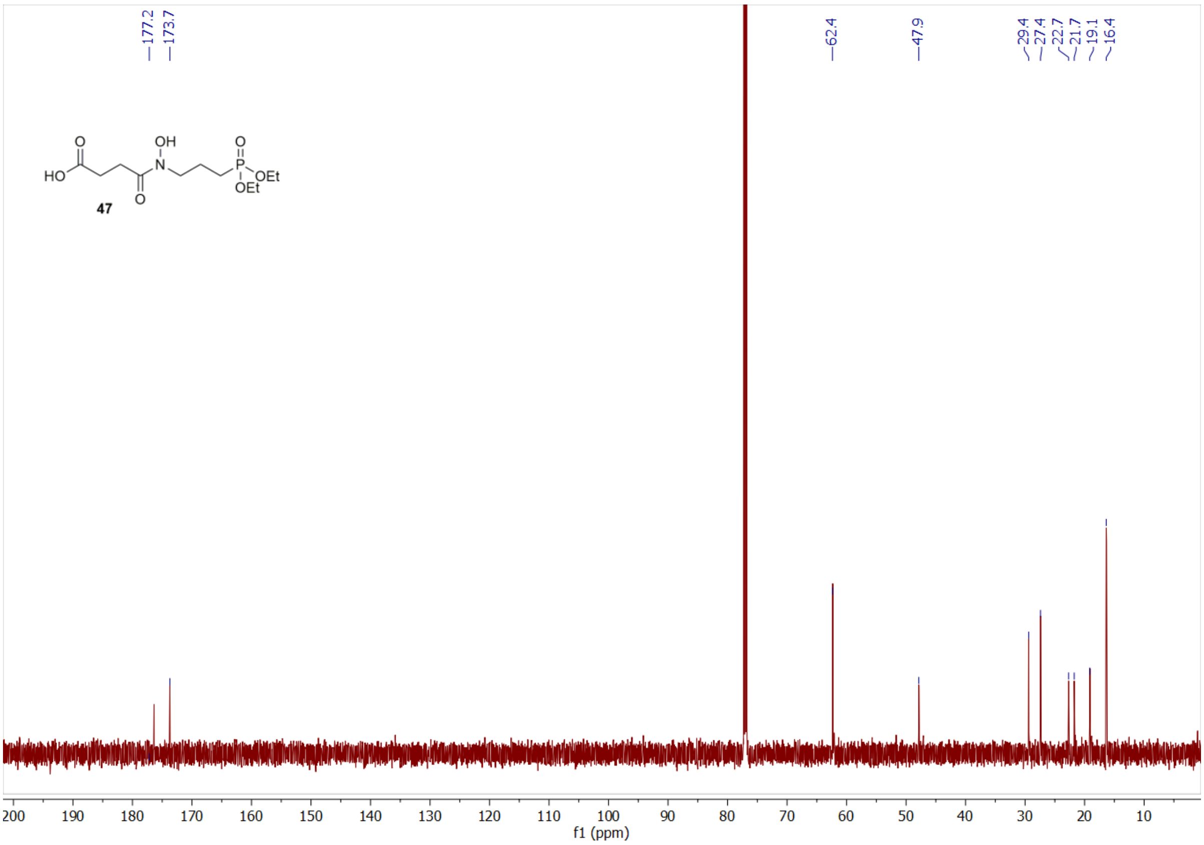

Figure S9. ^{13}C-NMR spectrum of compound 47.

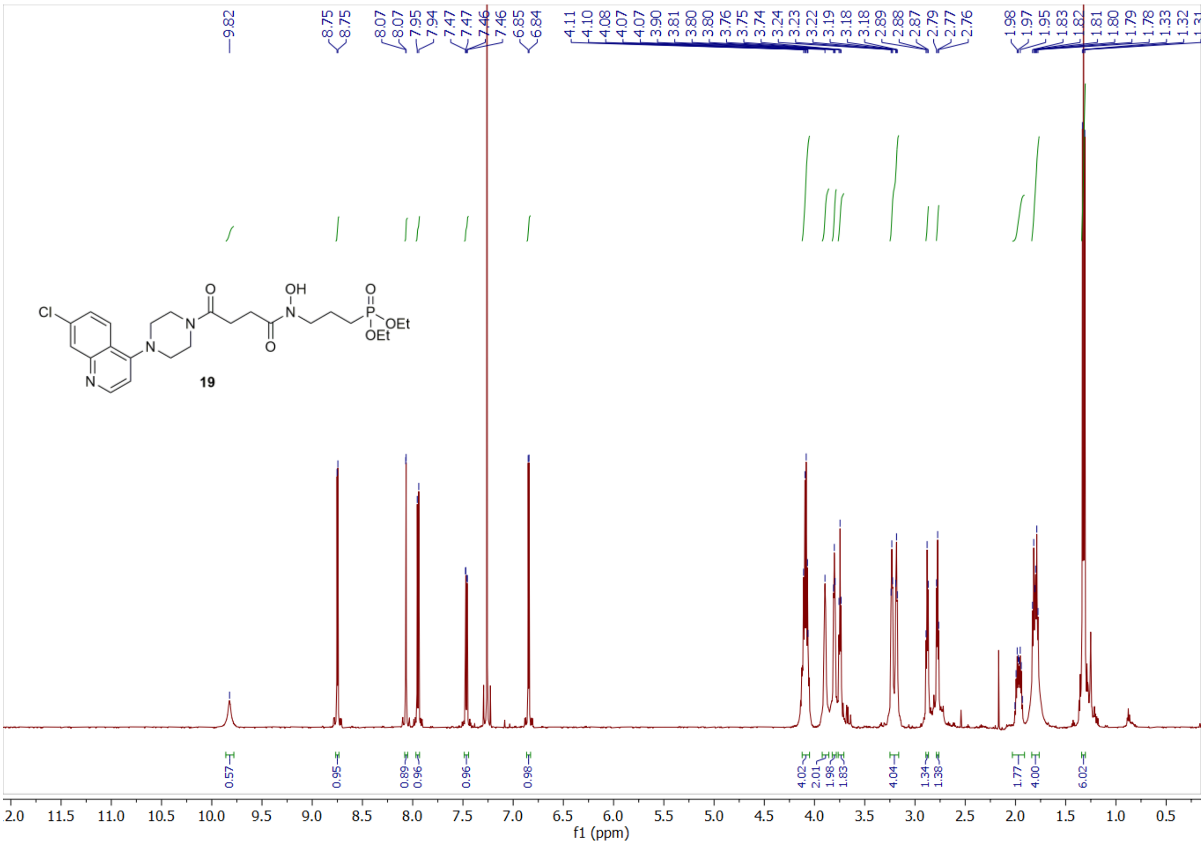

Figure S10. ^{1}H-NMR spectrum of compound 19.

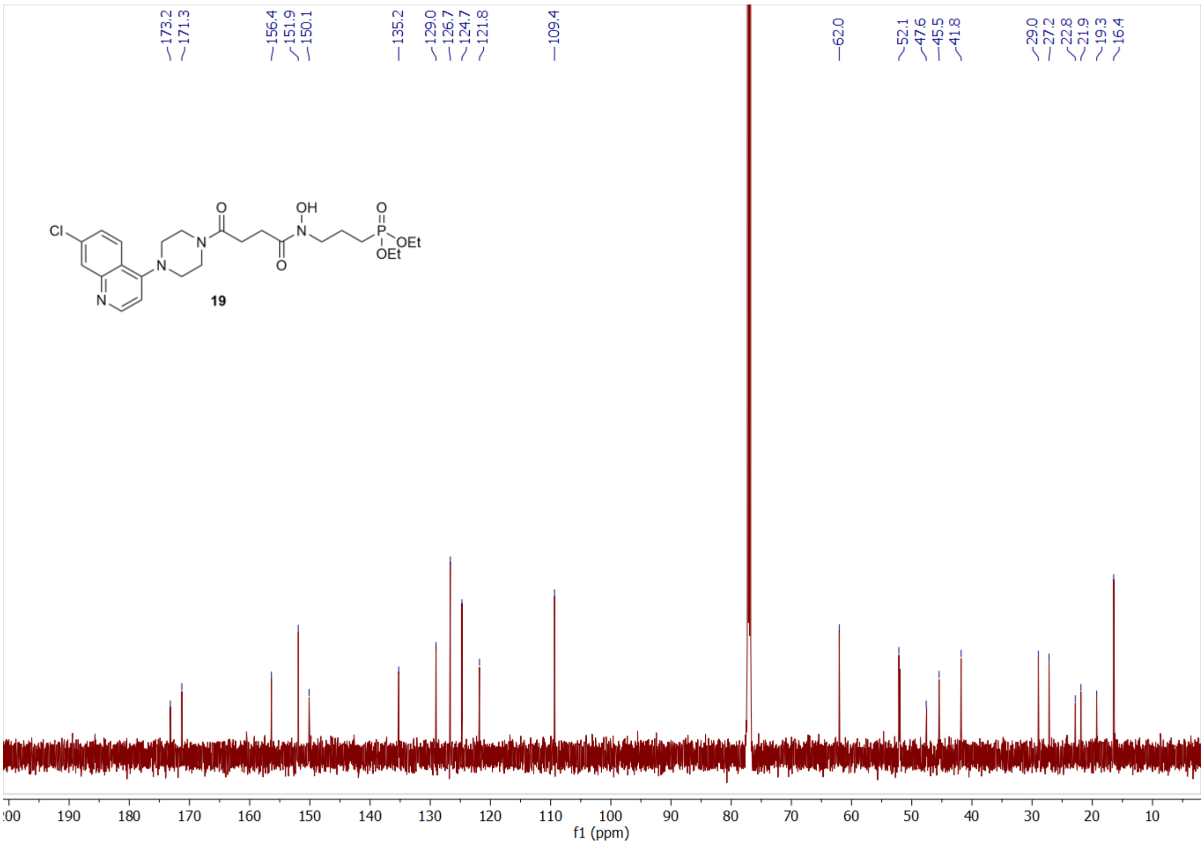

Figure S11. ^{13}**C-NMR spectrum of compound 19.**

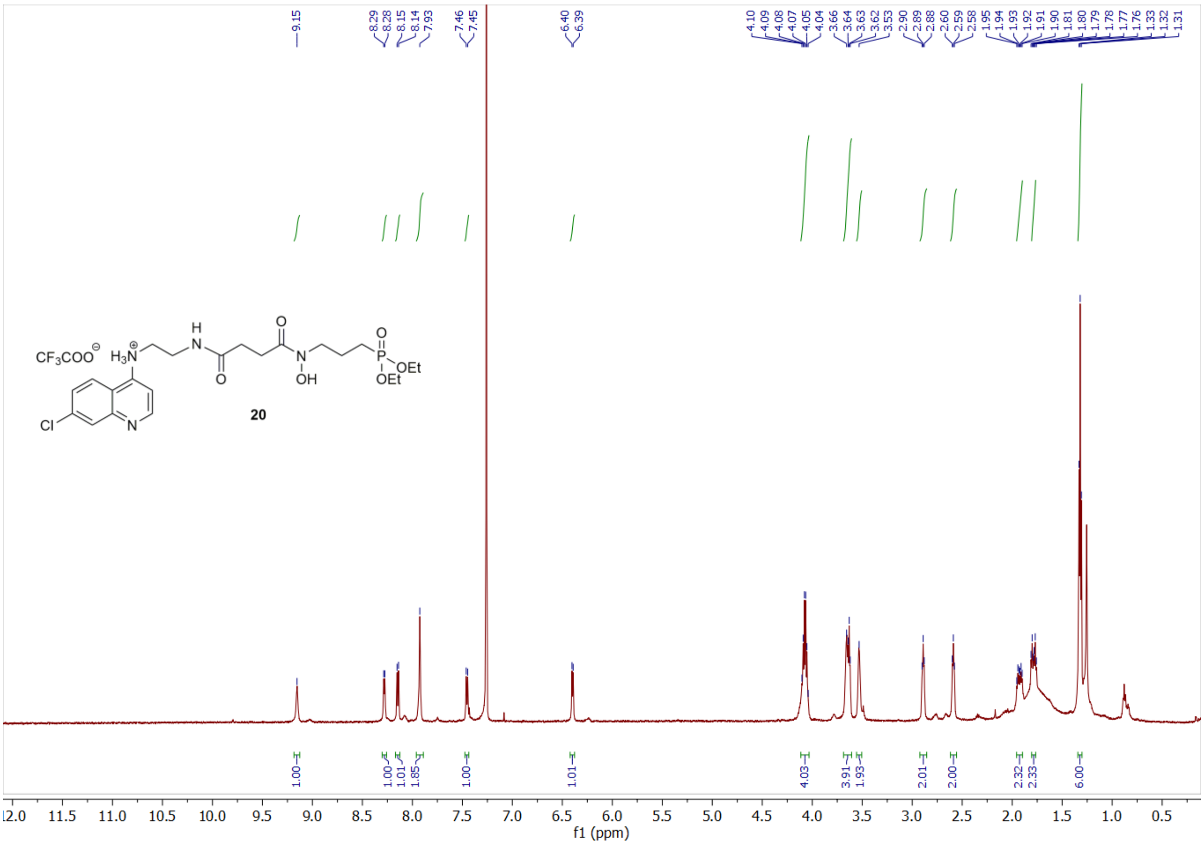

Figure S12. ^{1}**H-NMR spectrum of compound 20.**

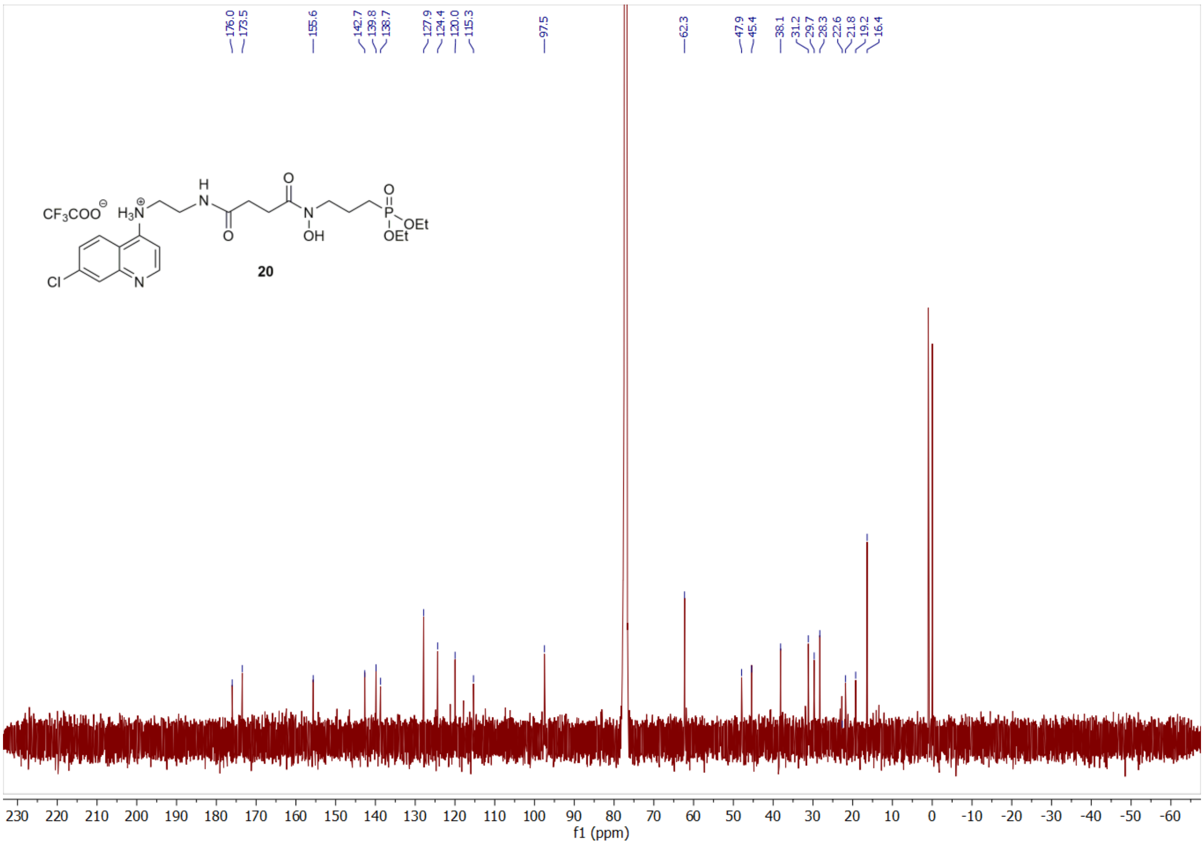

Figure S13. **^{13}C-NMR spectrum of compound 20.**

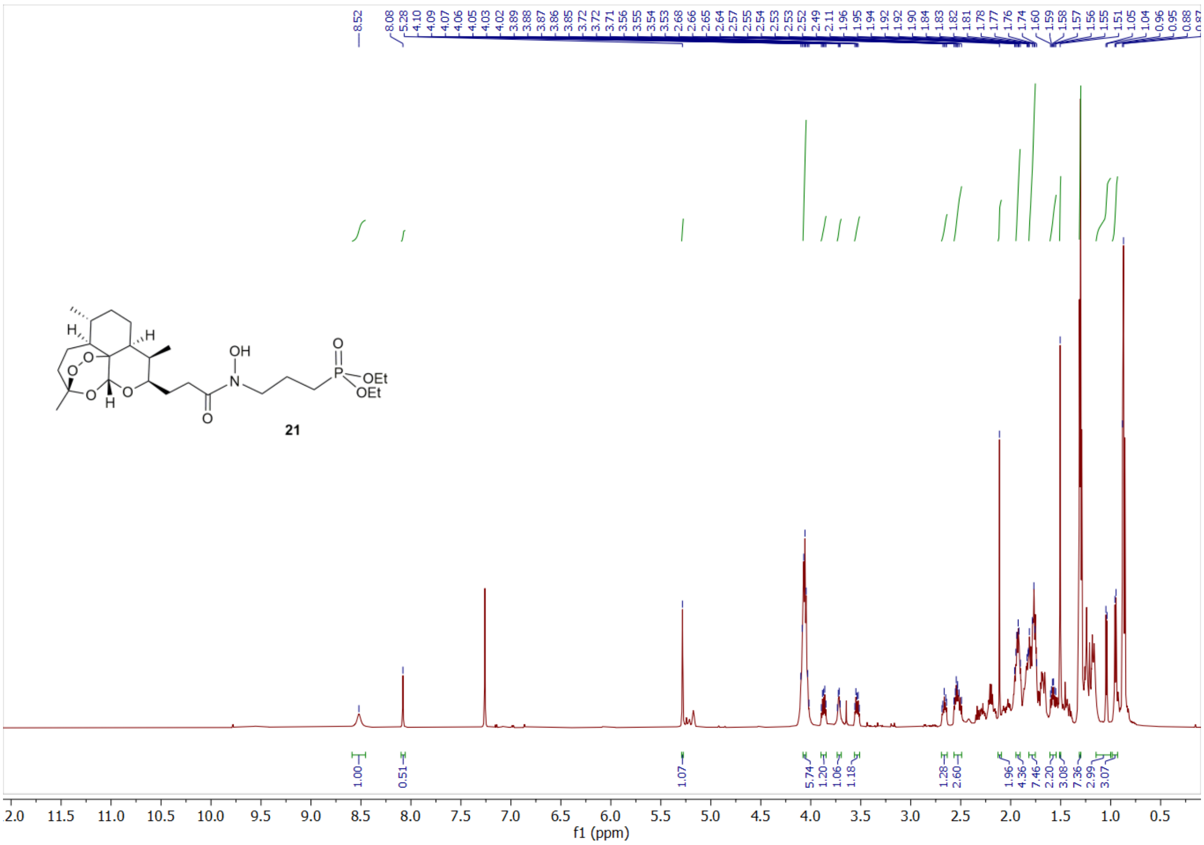

Figure S14. **^{1}H-NMR spectrum of compound 21.**

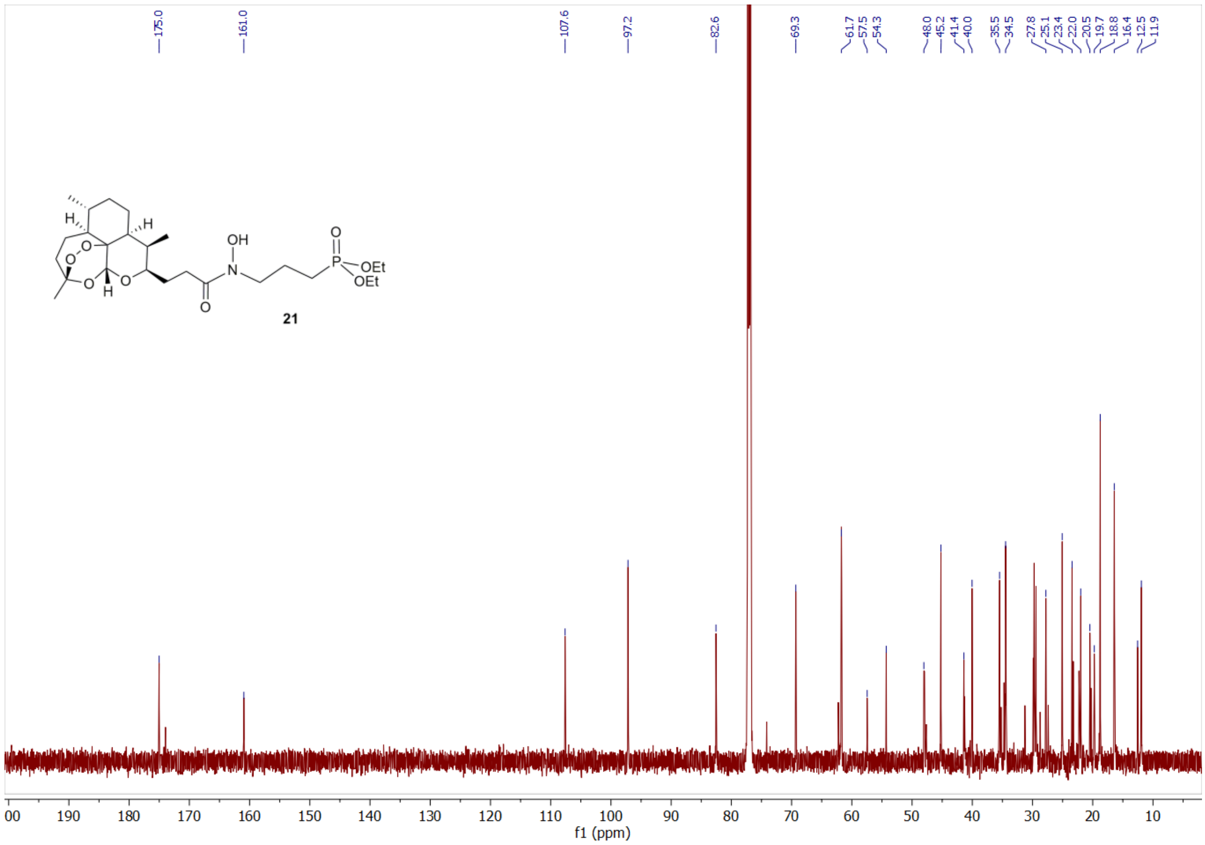

Figure S15. ^{13}C-NMR spectrum of compound 21.

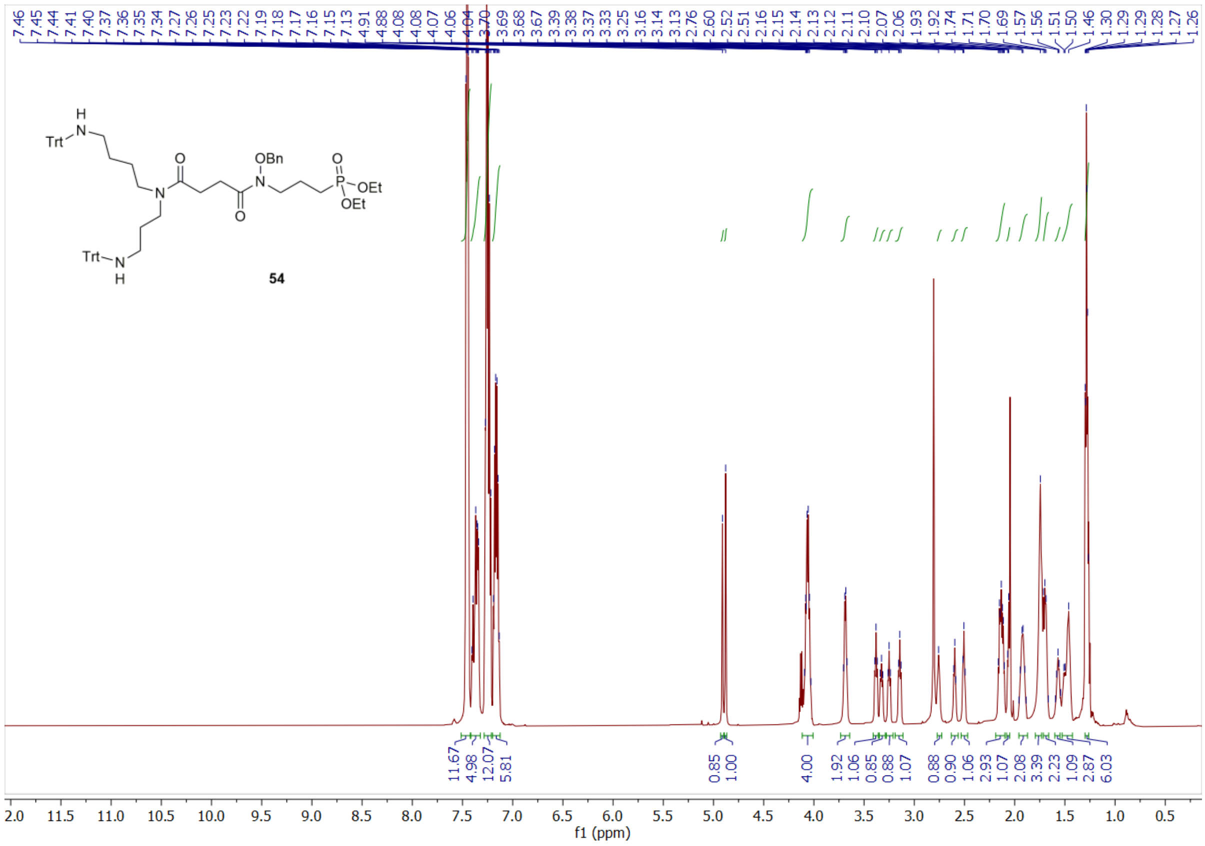

Figure S16. ^{1}H-NMR spectrum of compound 54.

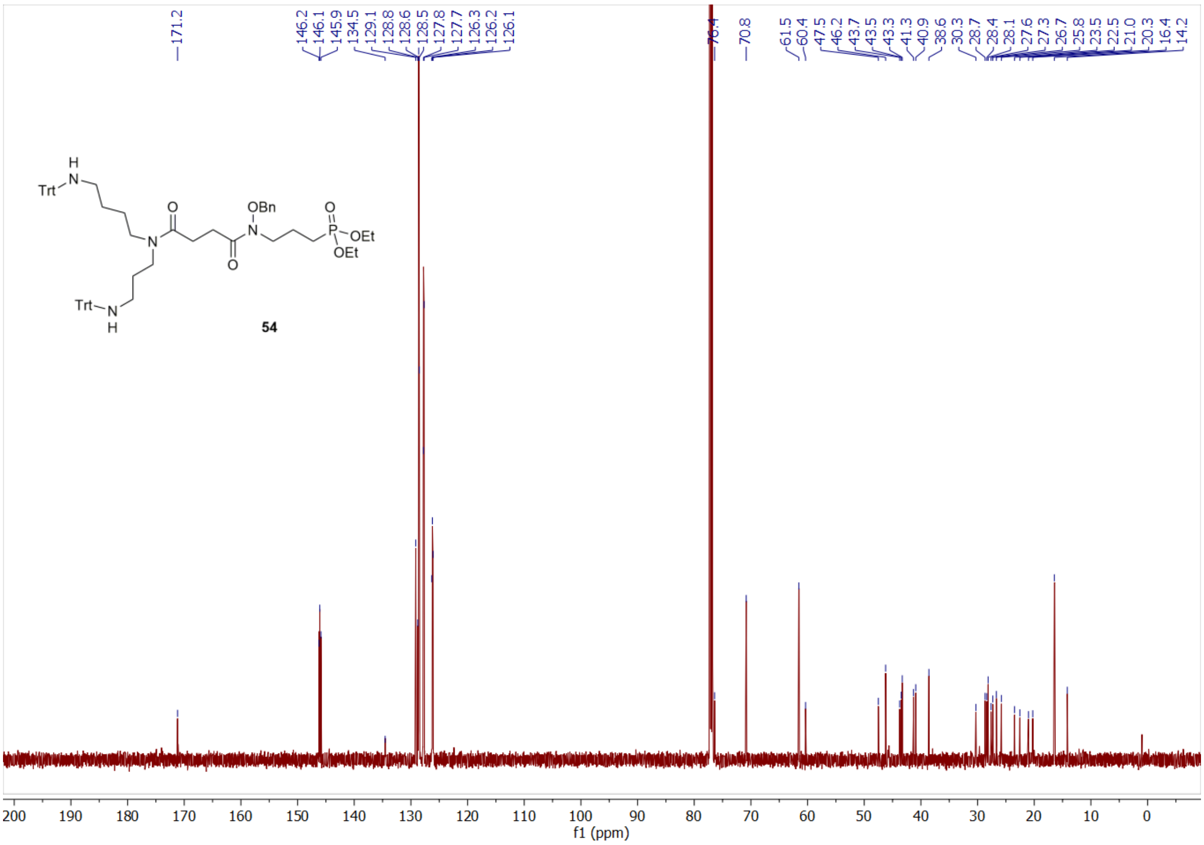

Figure S17. ^{13}**C-NMR spectrum of compound 54.**

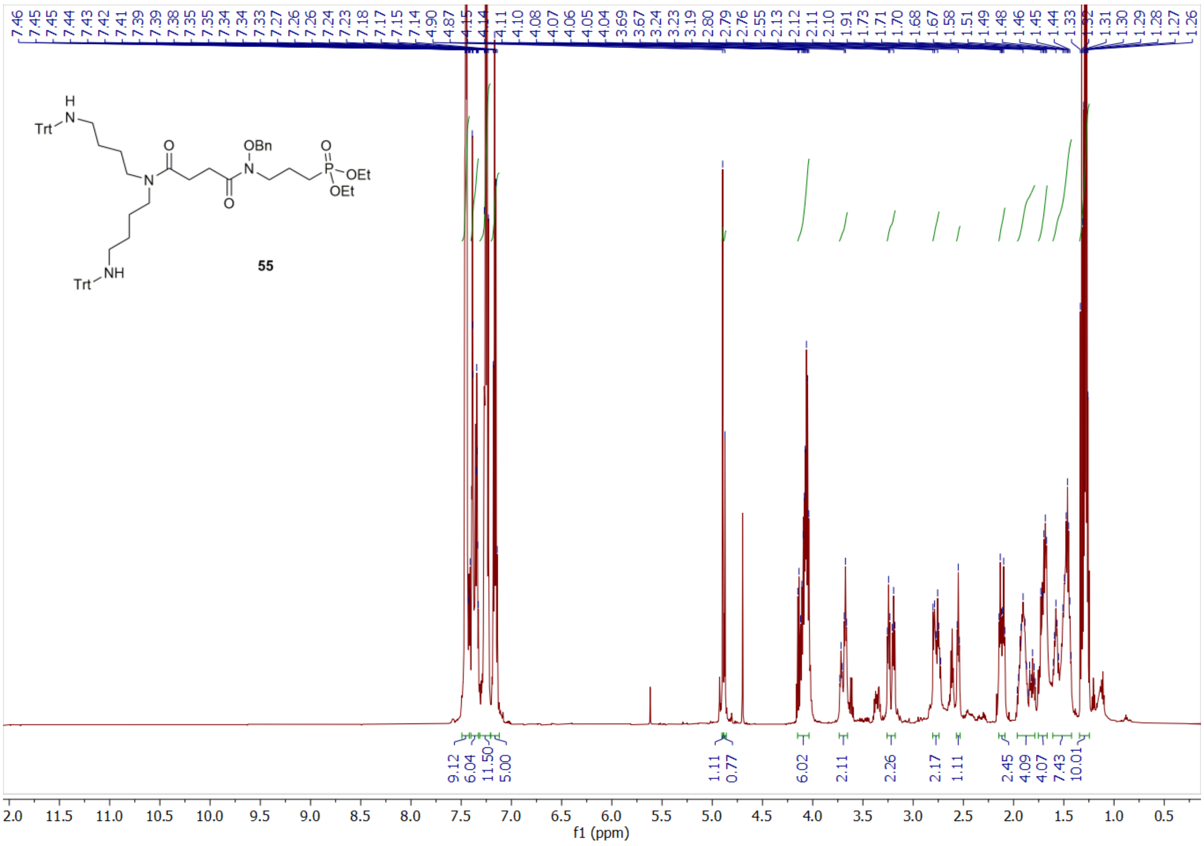

Figure S18. ^{1}**H-NMR spectrum of compound 55.**

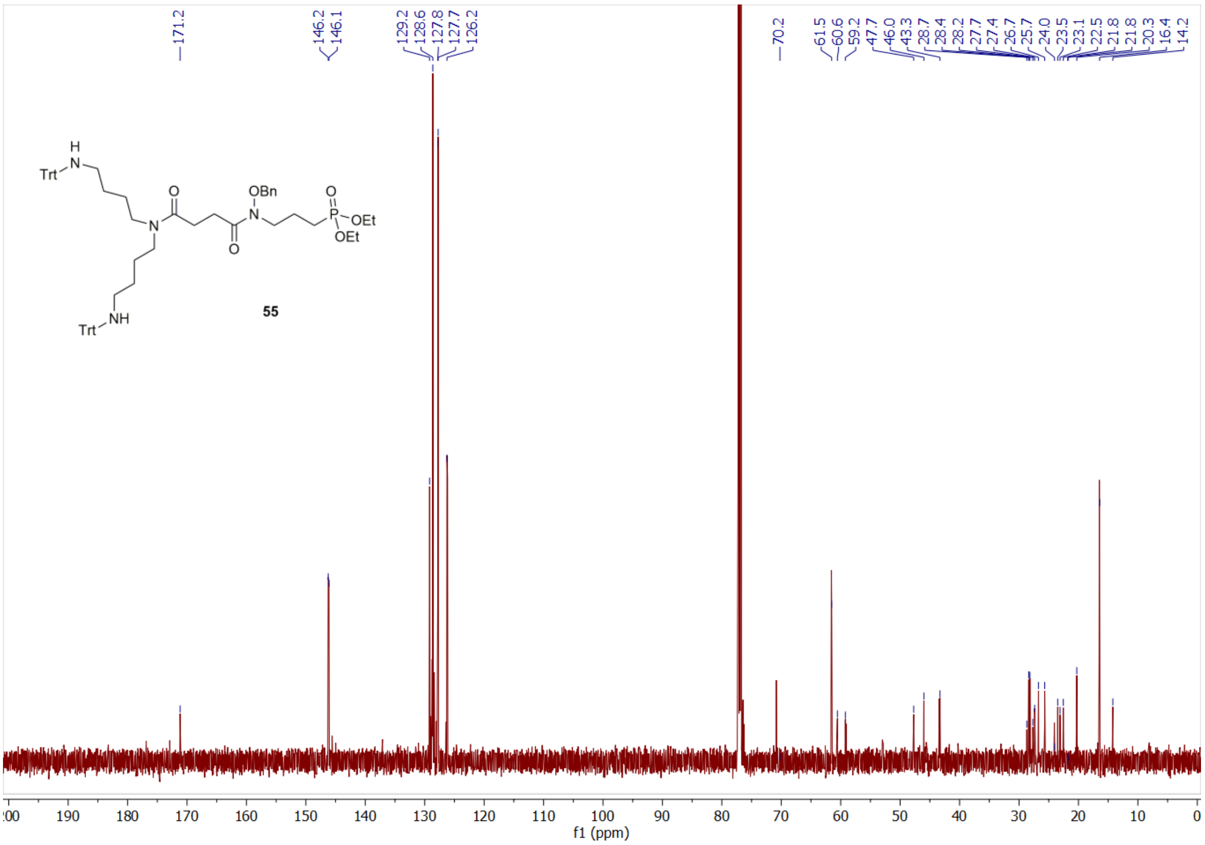

Figure S19. ^{13}C-NMR spectrum of compound 55.

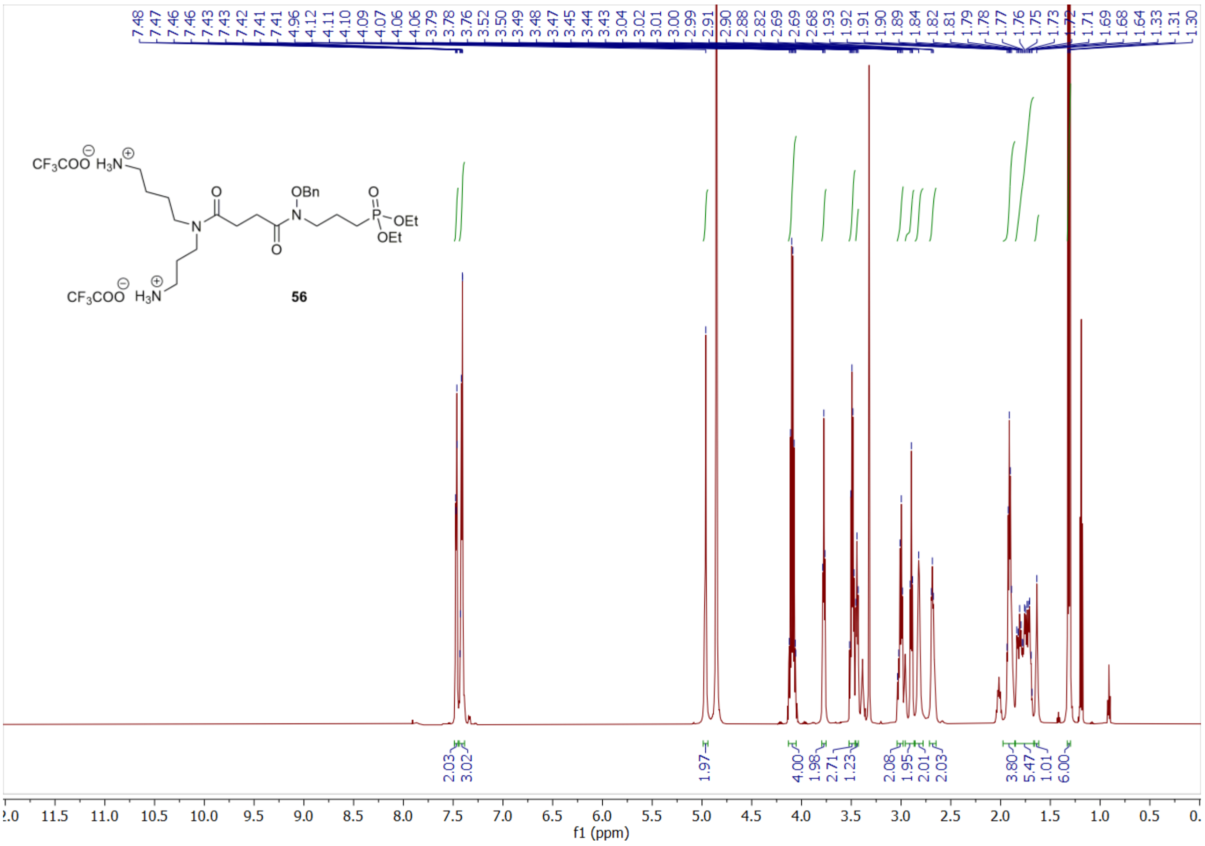

Figure S20. ^{1}H-NMR spectrum of compound 56.

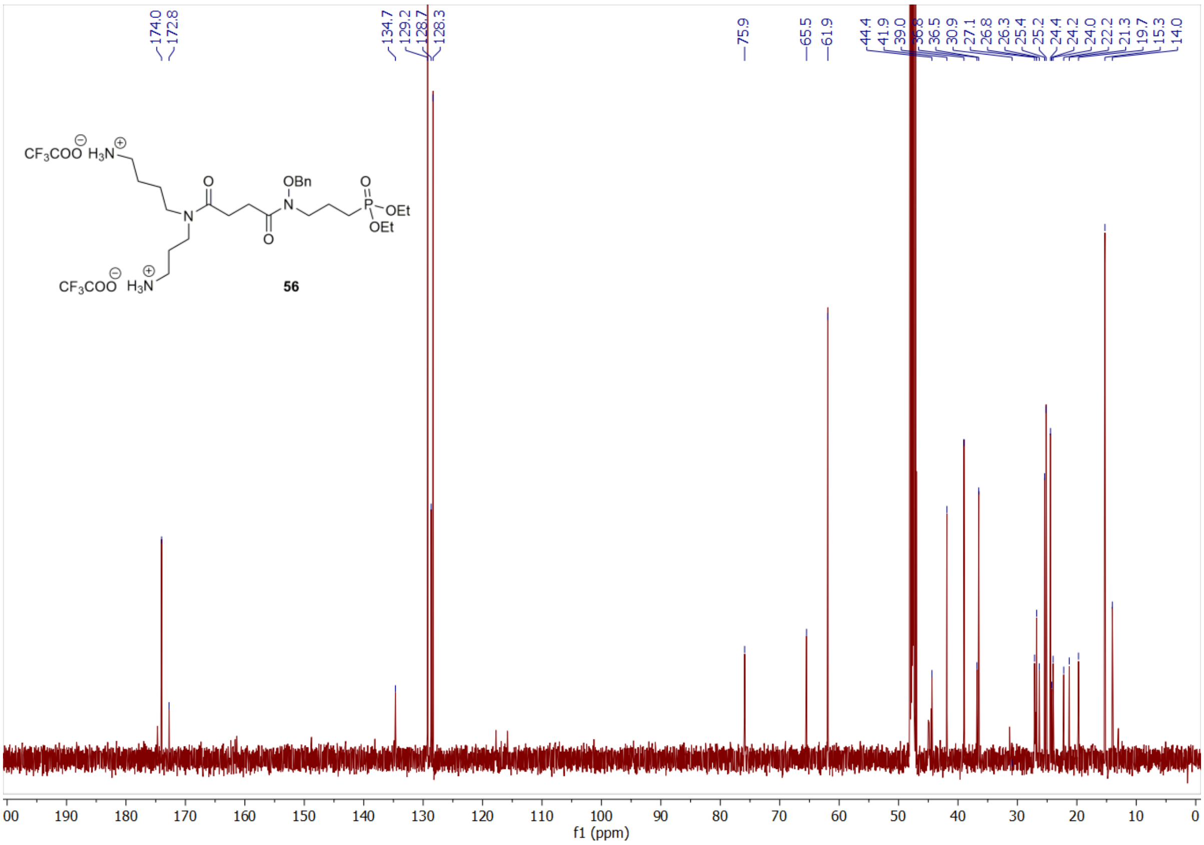

Figure S21. **^{13}C-NMR spectrum of compound 56.**

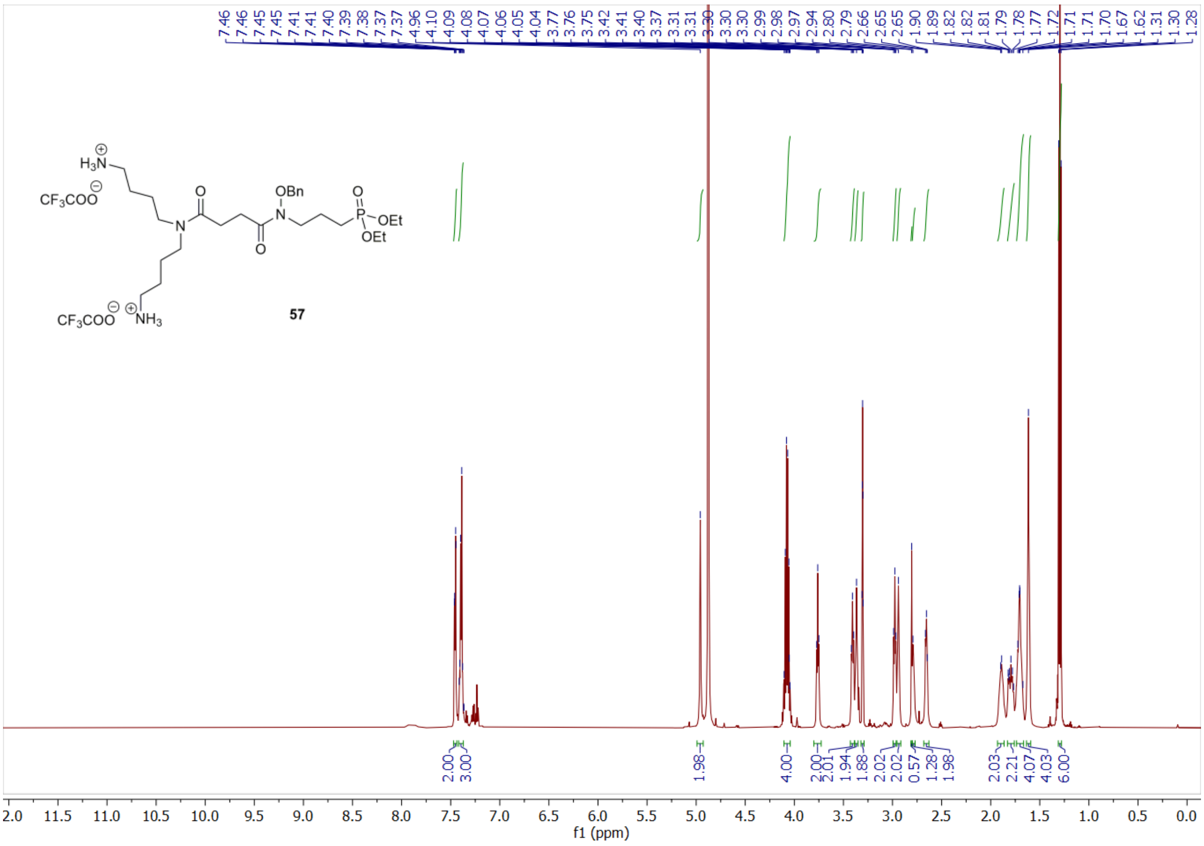

Figure S22. **^{1}H-NMR spectrum of compound 57.**

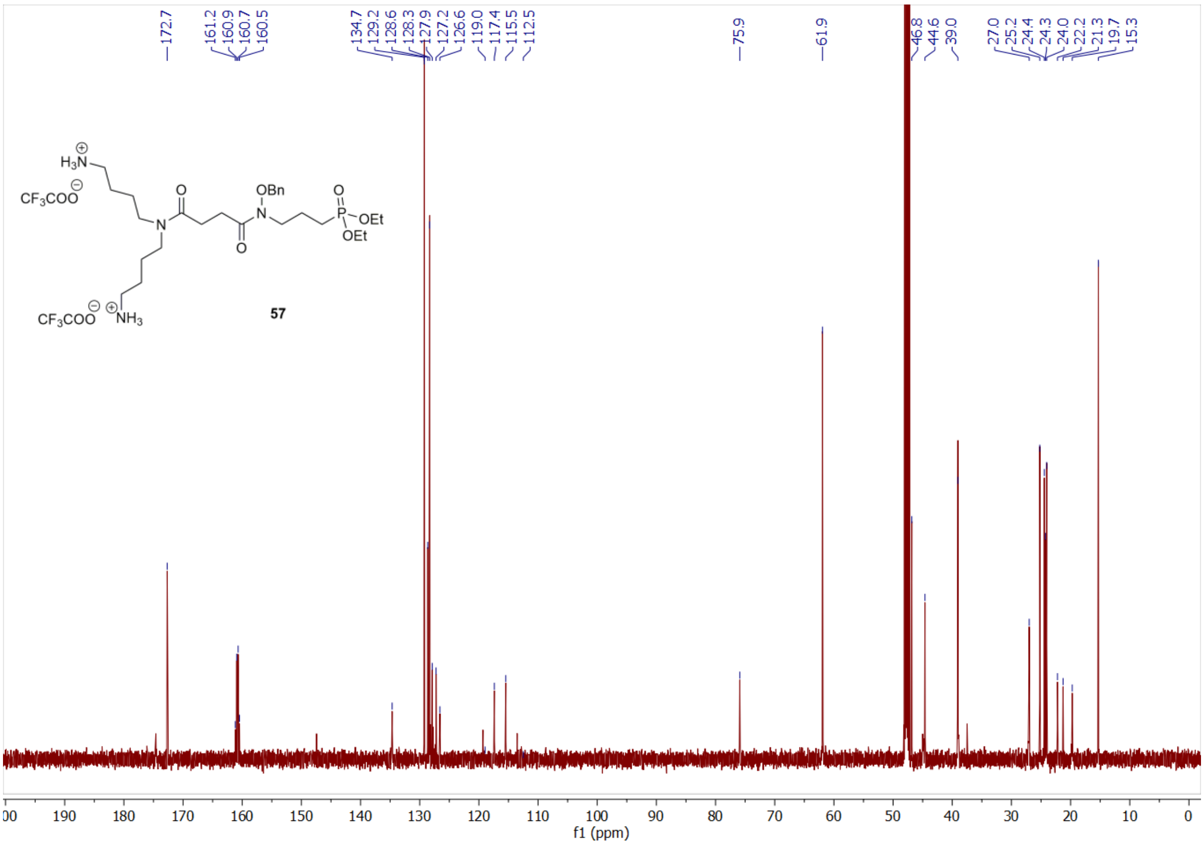

Figure S23. ^{13}**C-NMR spectrum of compound 57.**

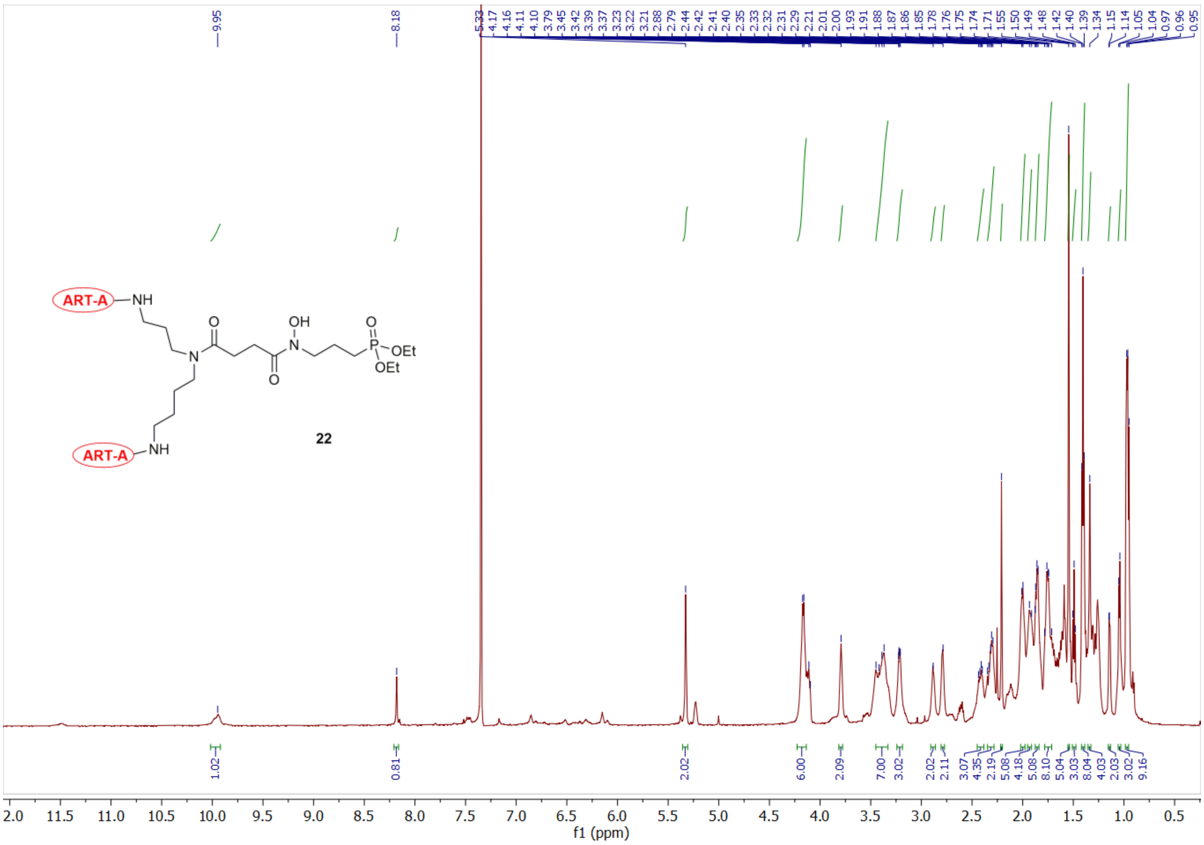

Figure S24. ^{1}**H-NMR spectrum of compound 22.**

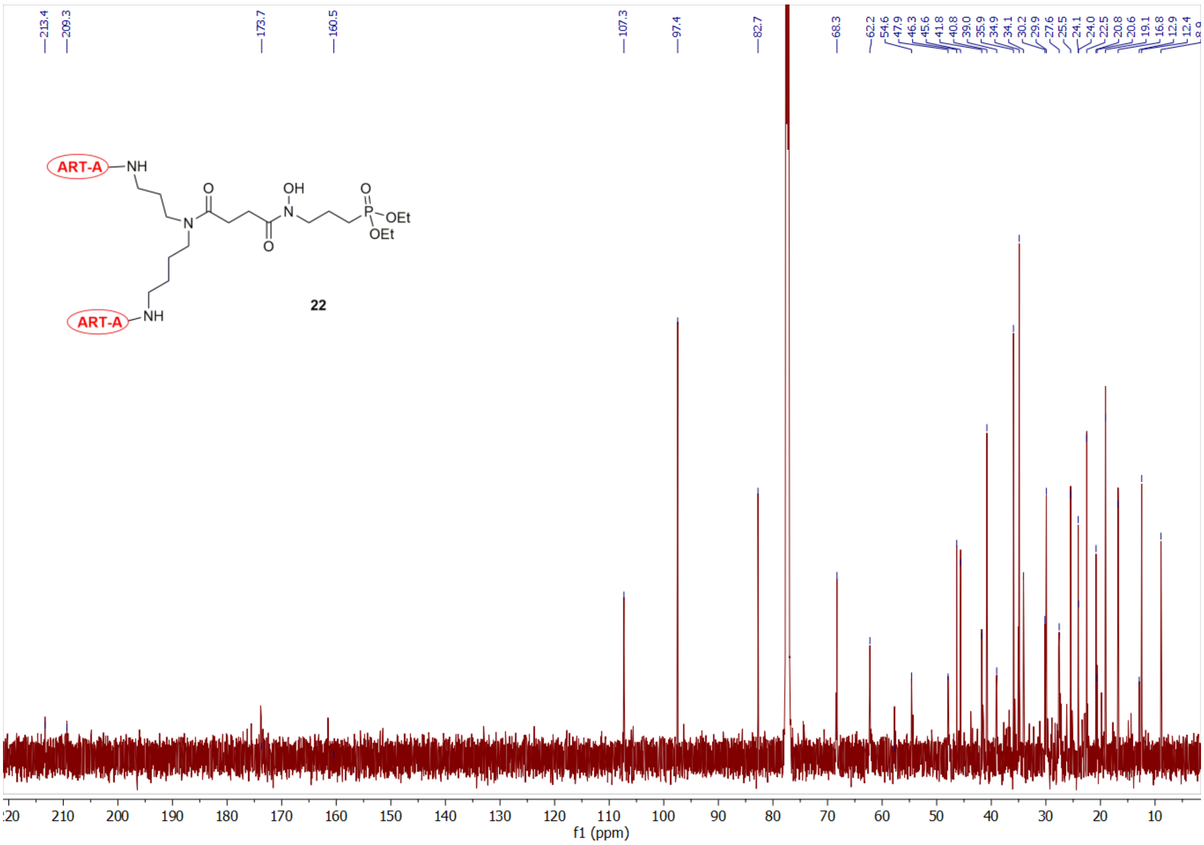

Figure S25. **^{13}C-NMR spectrum of compound 22.**

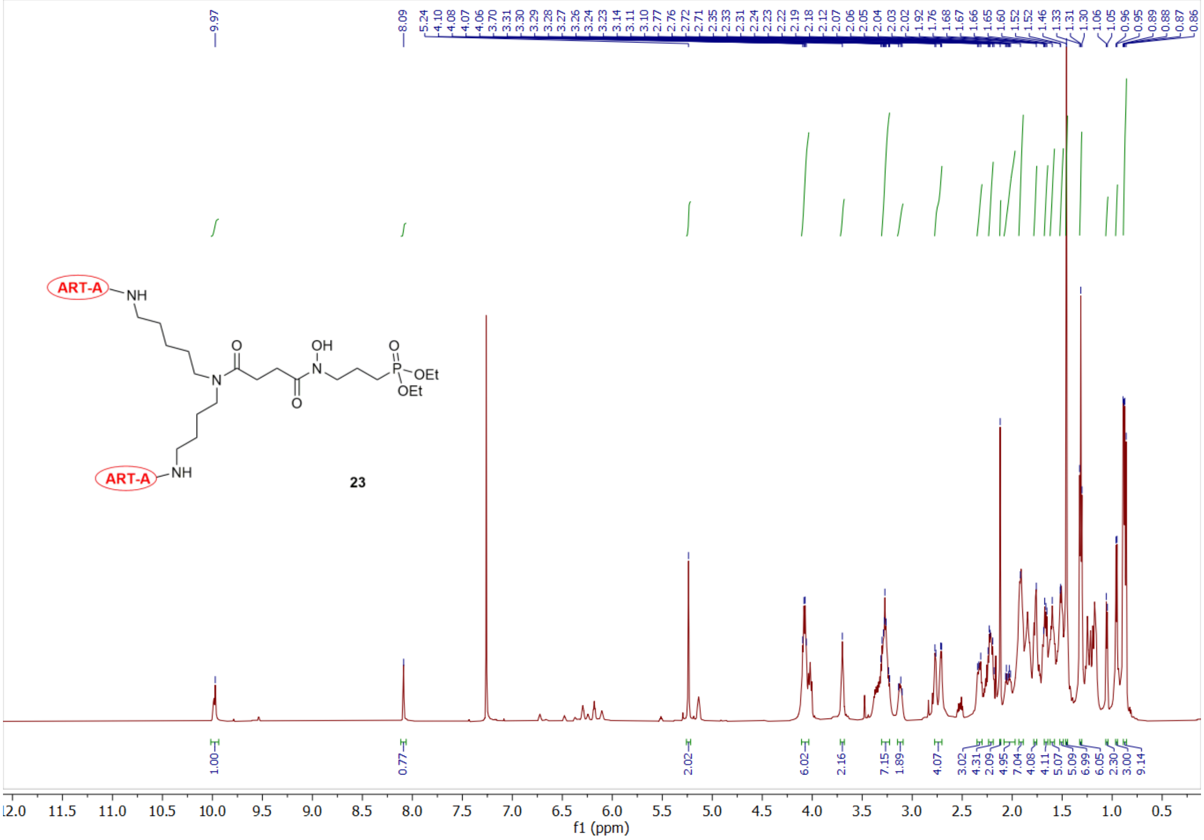

Figure S26. **^{1}H-NMR spectrum of compound 23.**

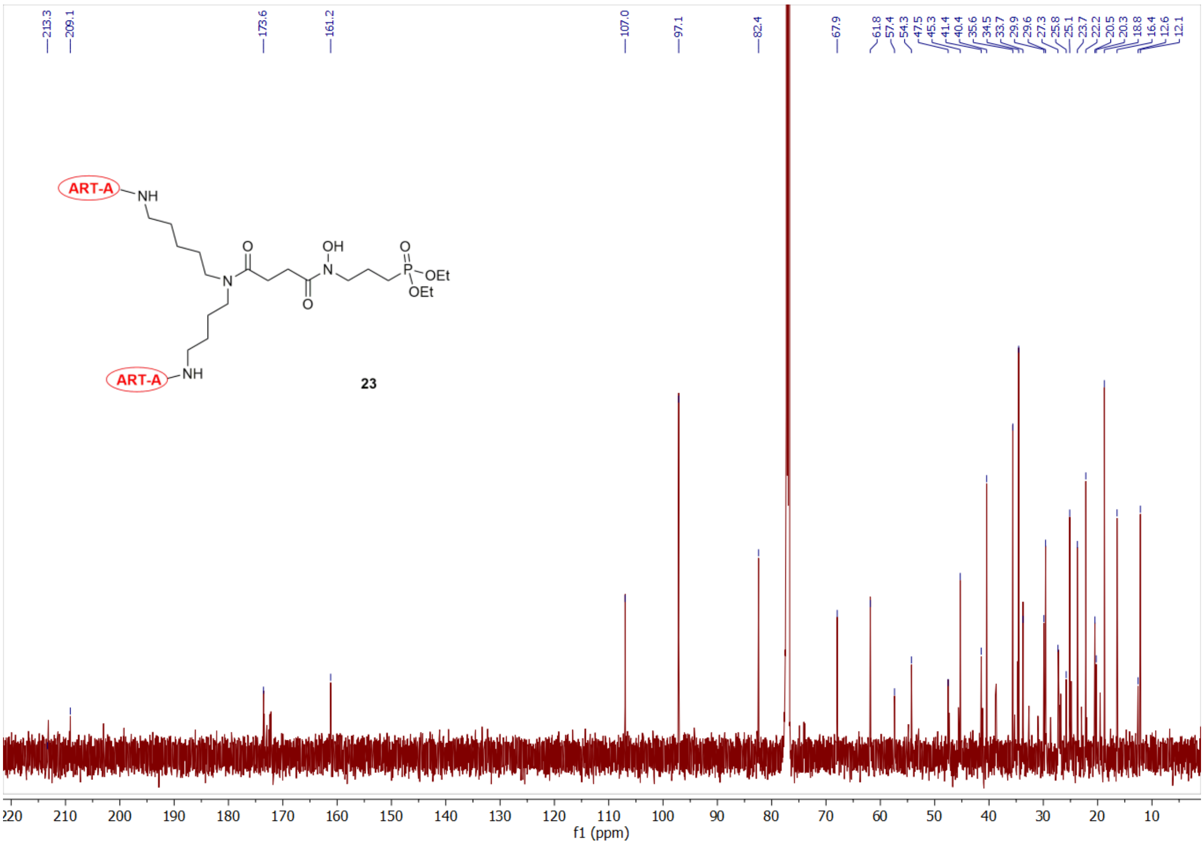

Figure S27. ^{13}C-NMR spectrum of compound 23.

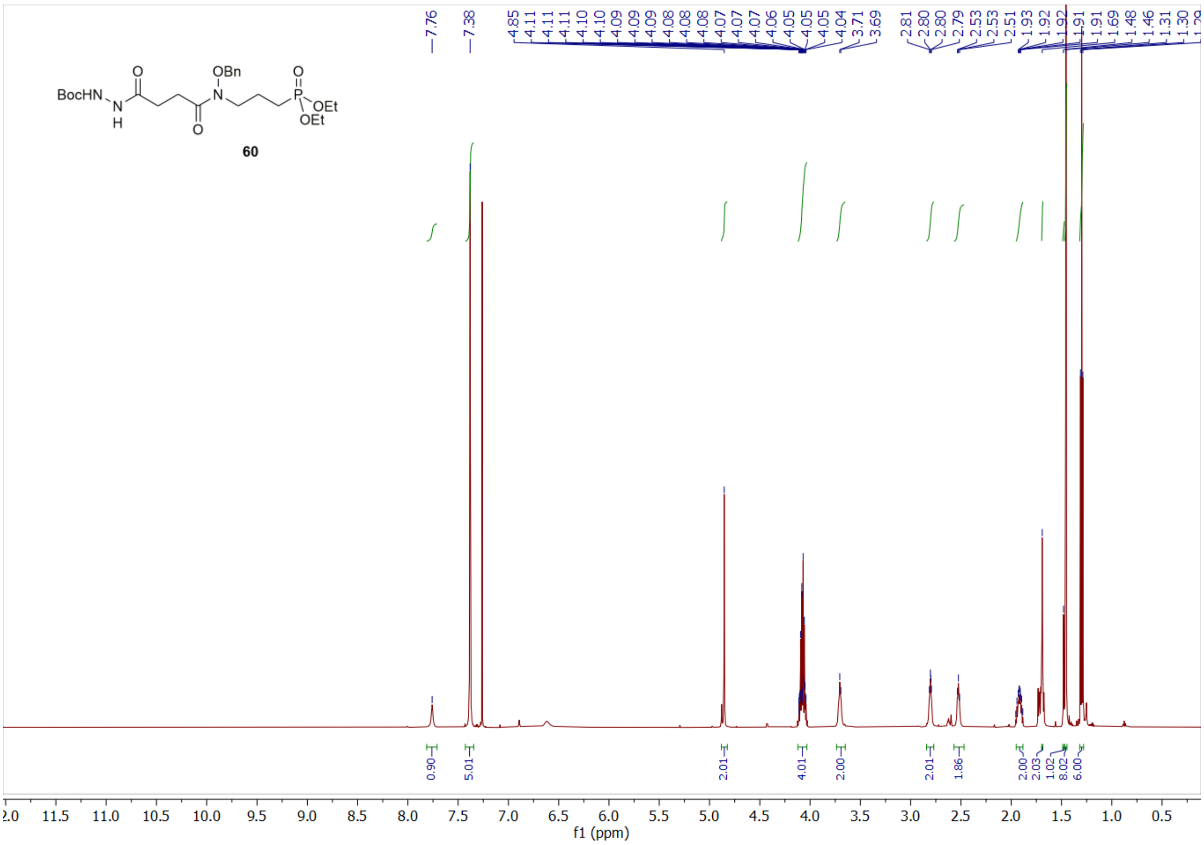

Figure S28. ^{1}H-NMR spectrum of compound 60.

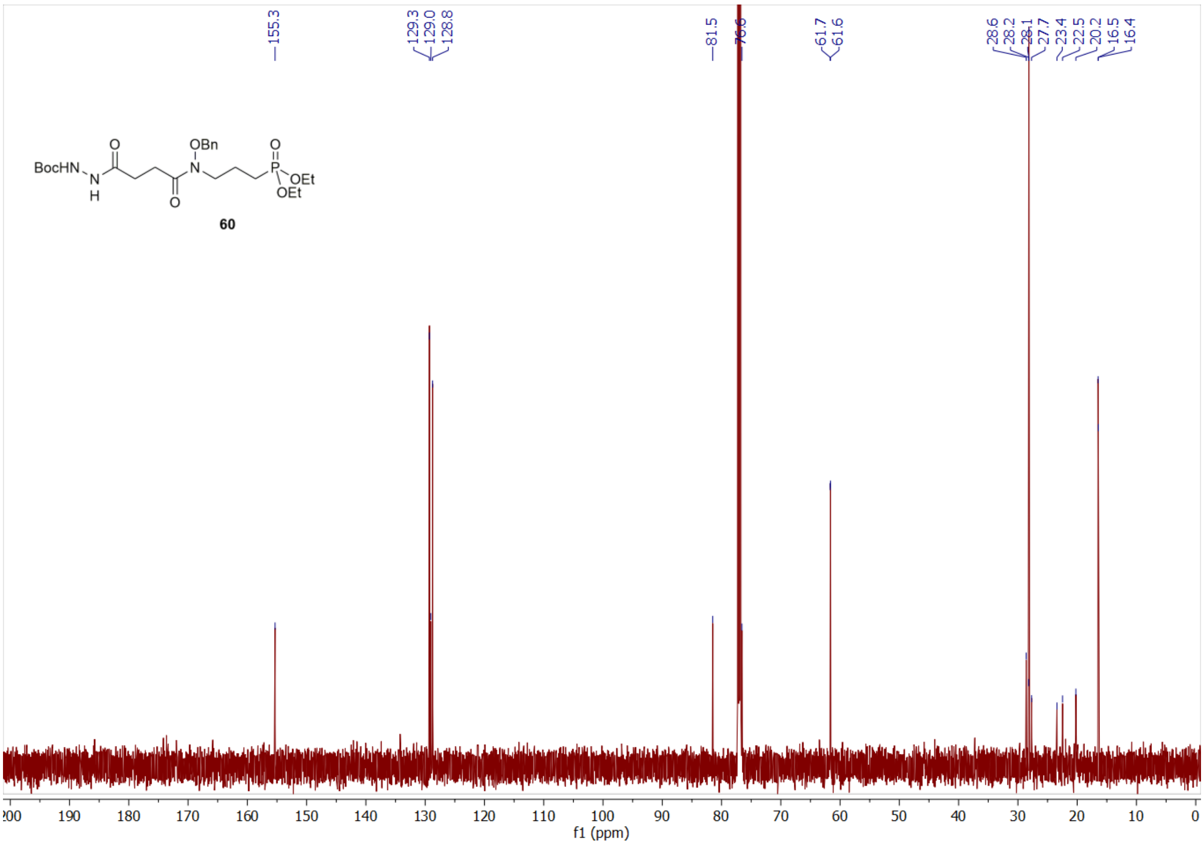

Figure S29. ^{13}**C-NMR spectrum of compound 60.**

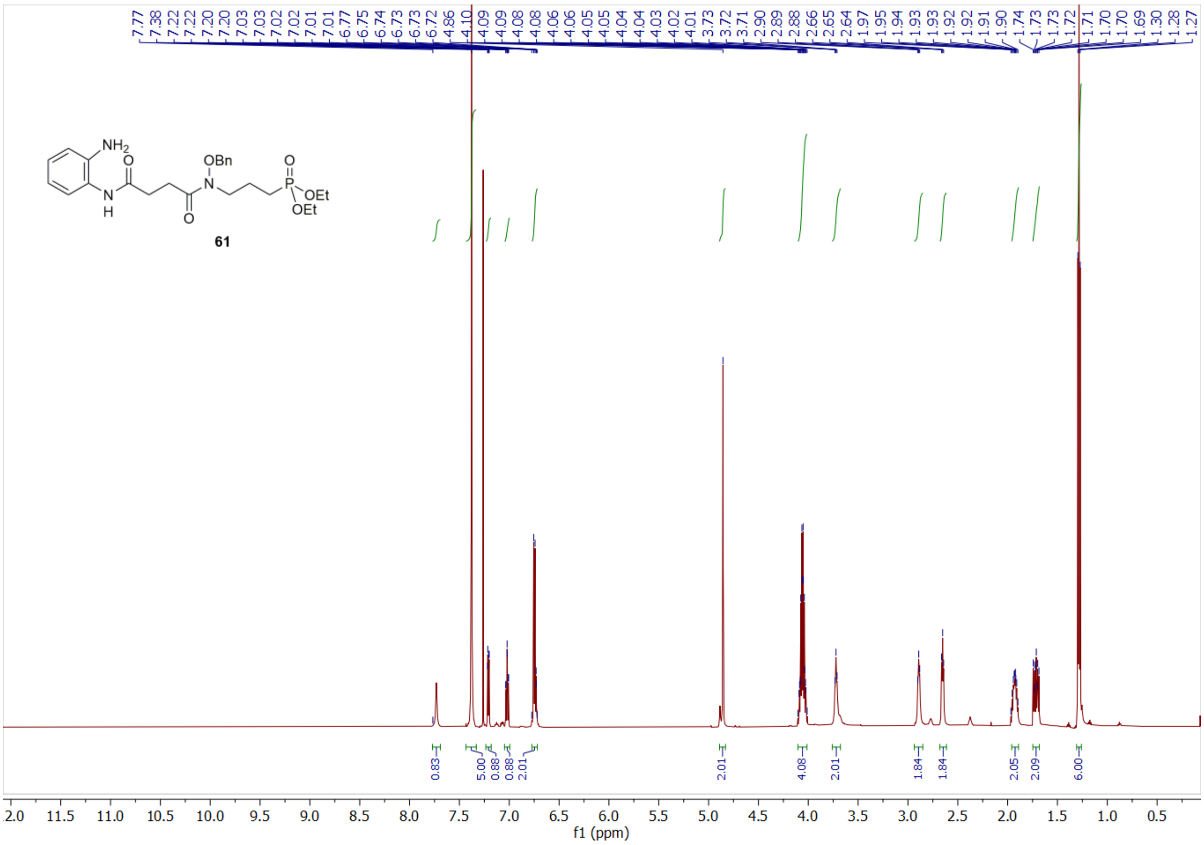

Figure S30. ^{1}**H-NMR spectrum of compound 61.**

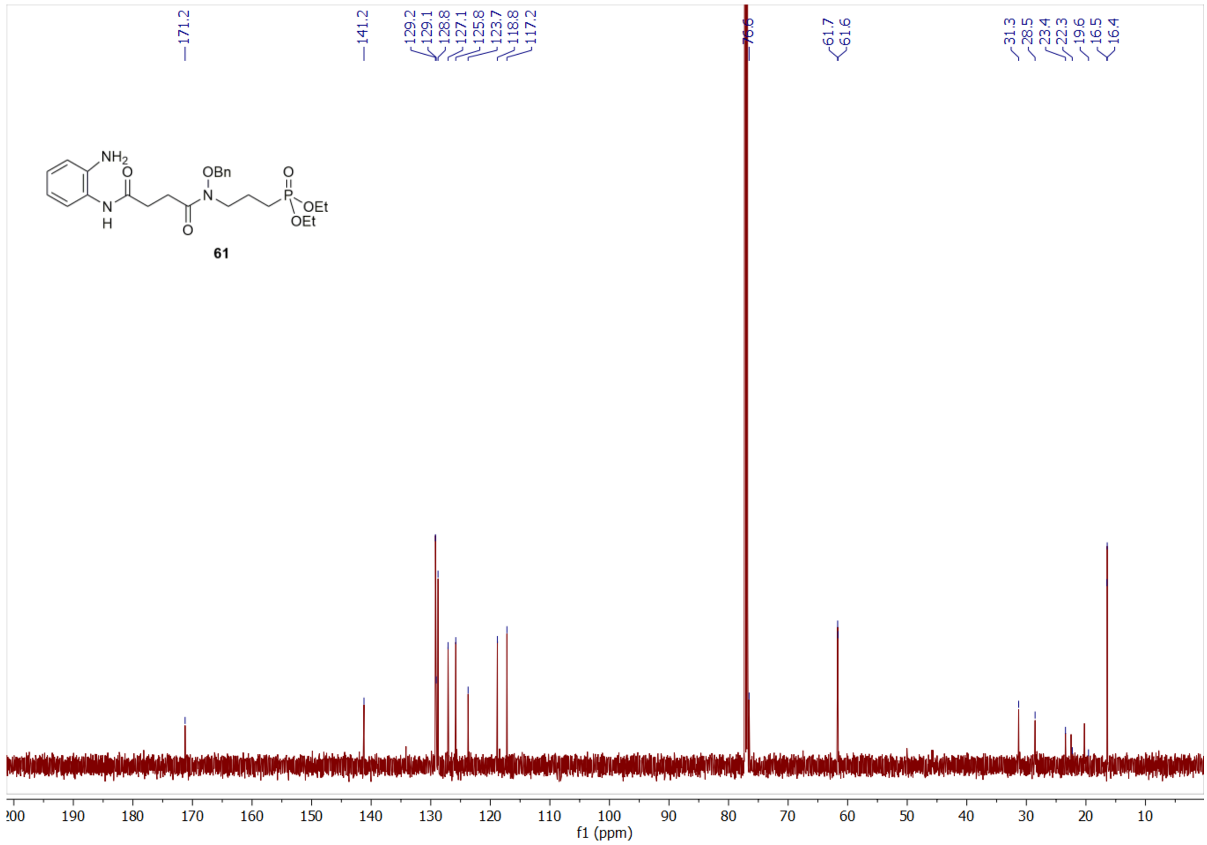

Figure S31. ^{13}C-NMR spectrum of compound 61.

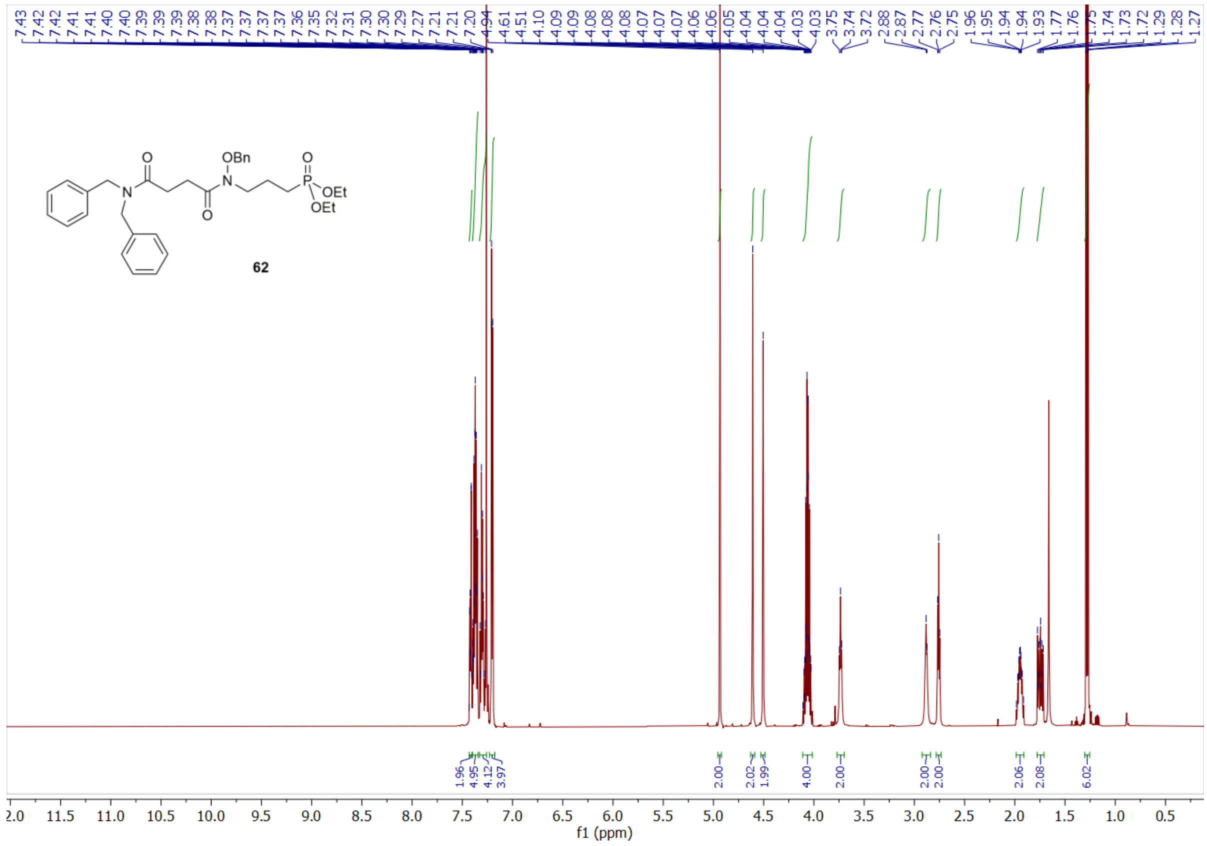

Figure S32. ^{1}H-NMR spectrum of compound 62.

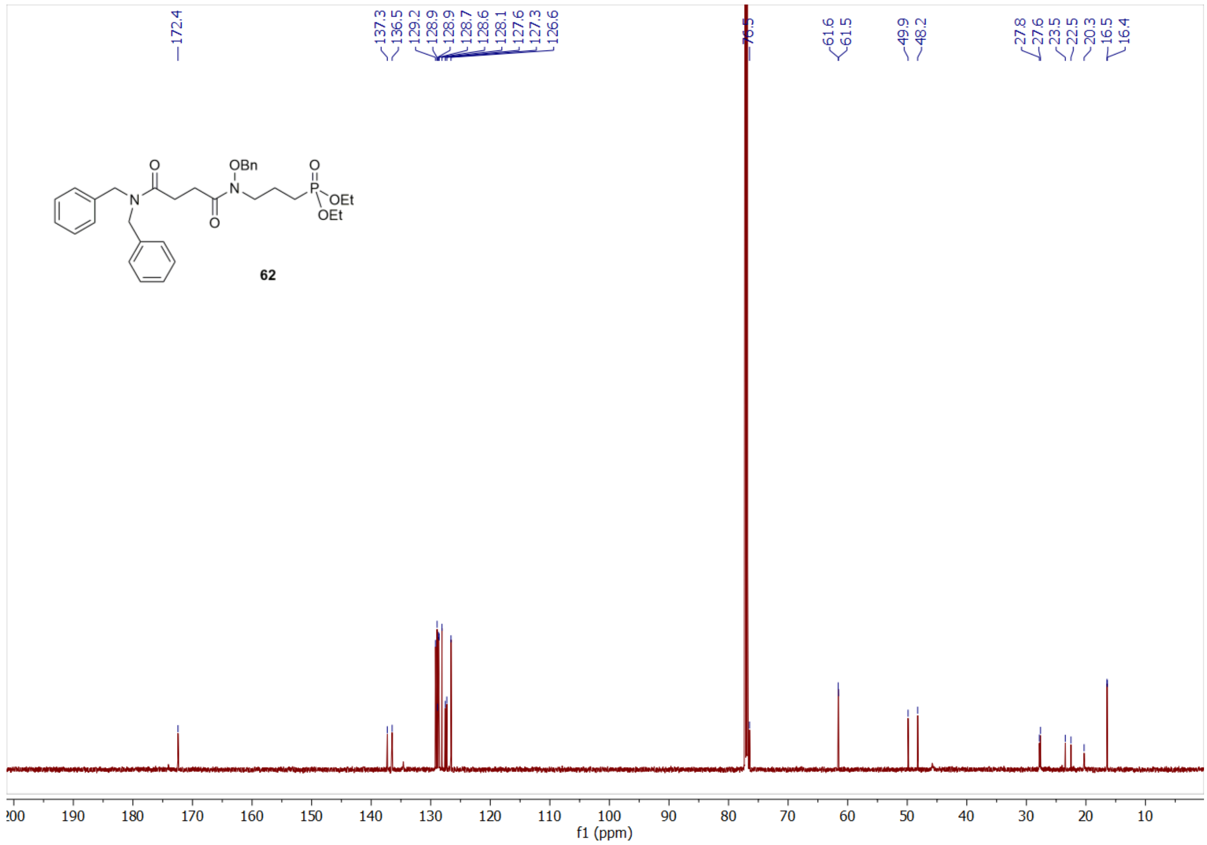

Figure S33. ^{13}C-NMR spectrum of compound 62.

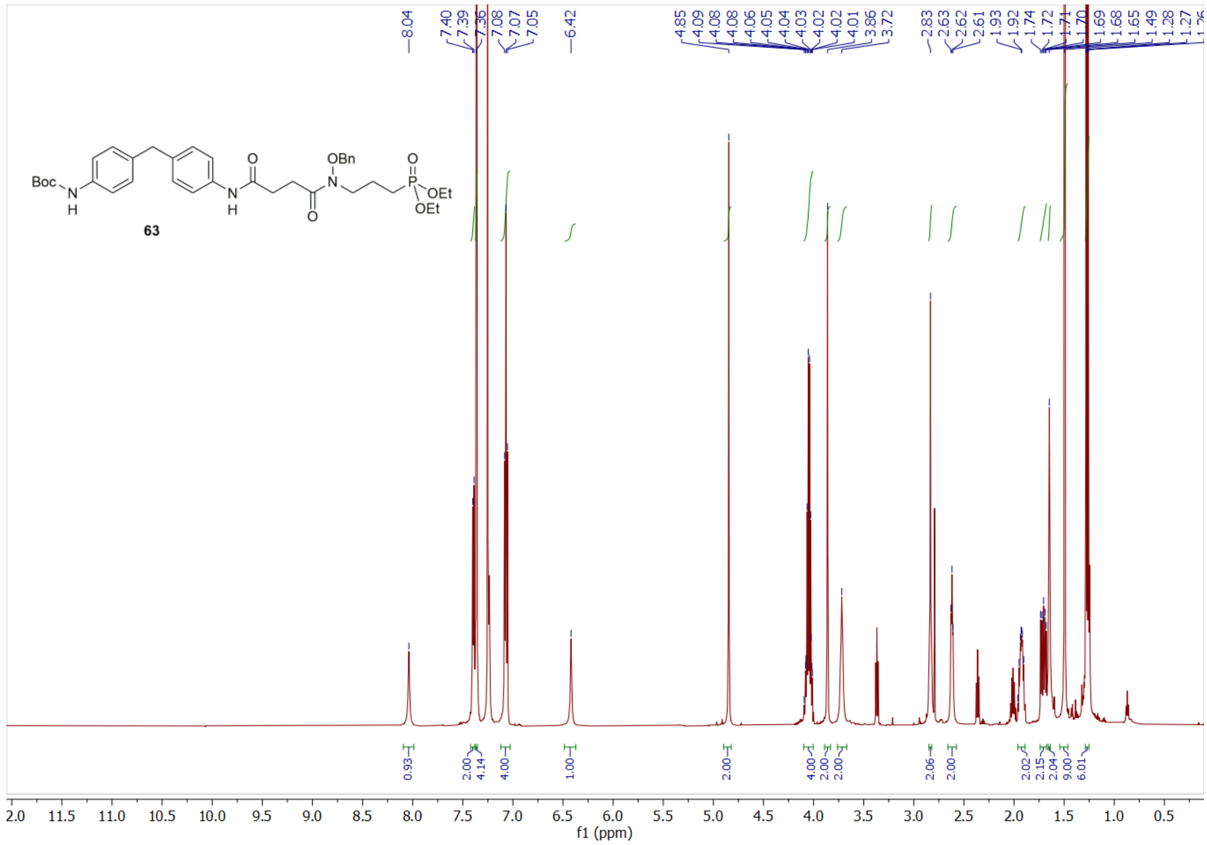

Figure S34. ^{1}H-NMR spectrum of compound 63.

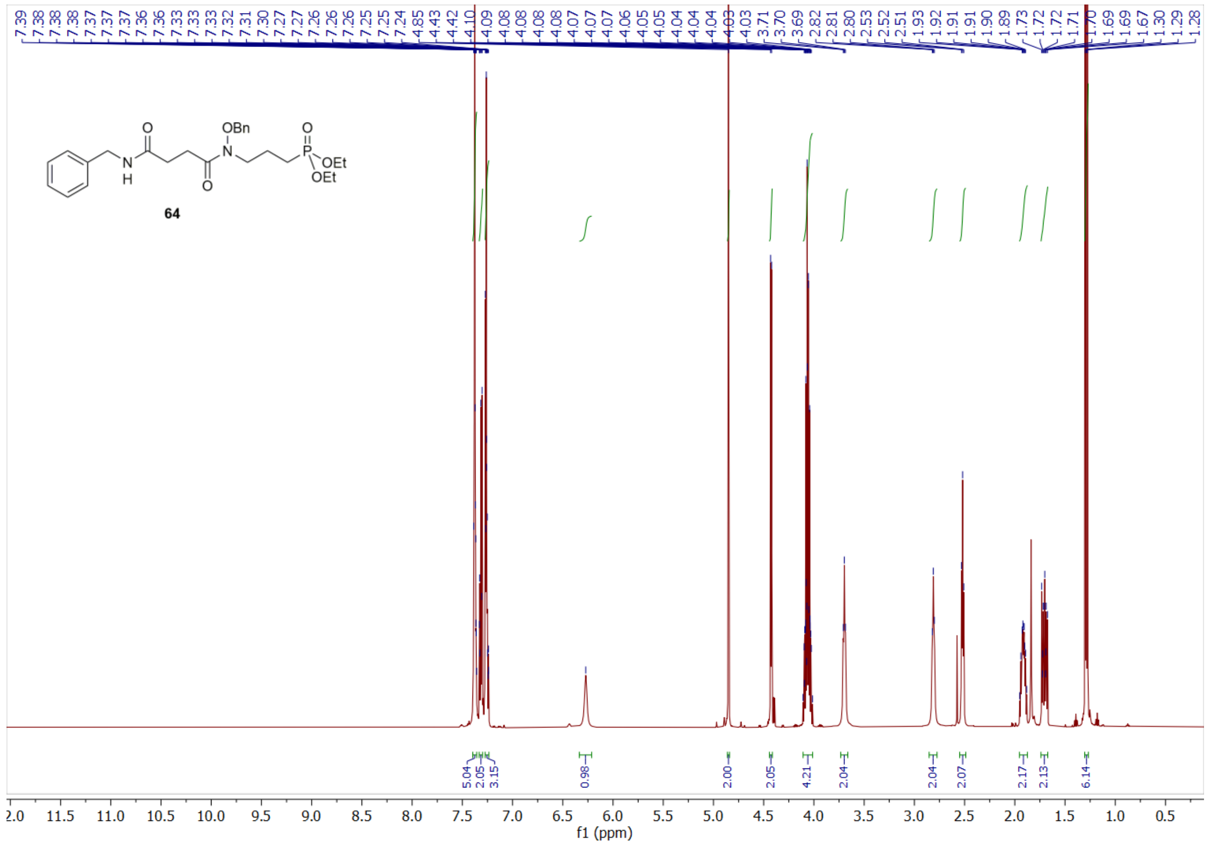

Figure S35. ^{1}**H-NMR spectrum of compound 64.**

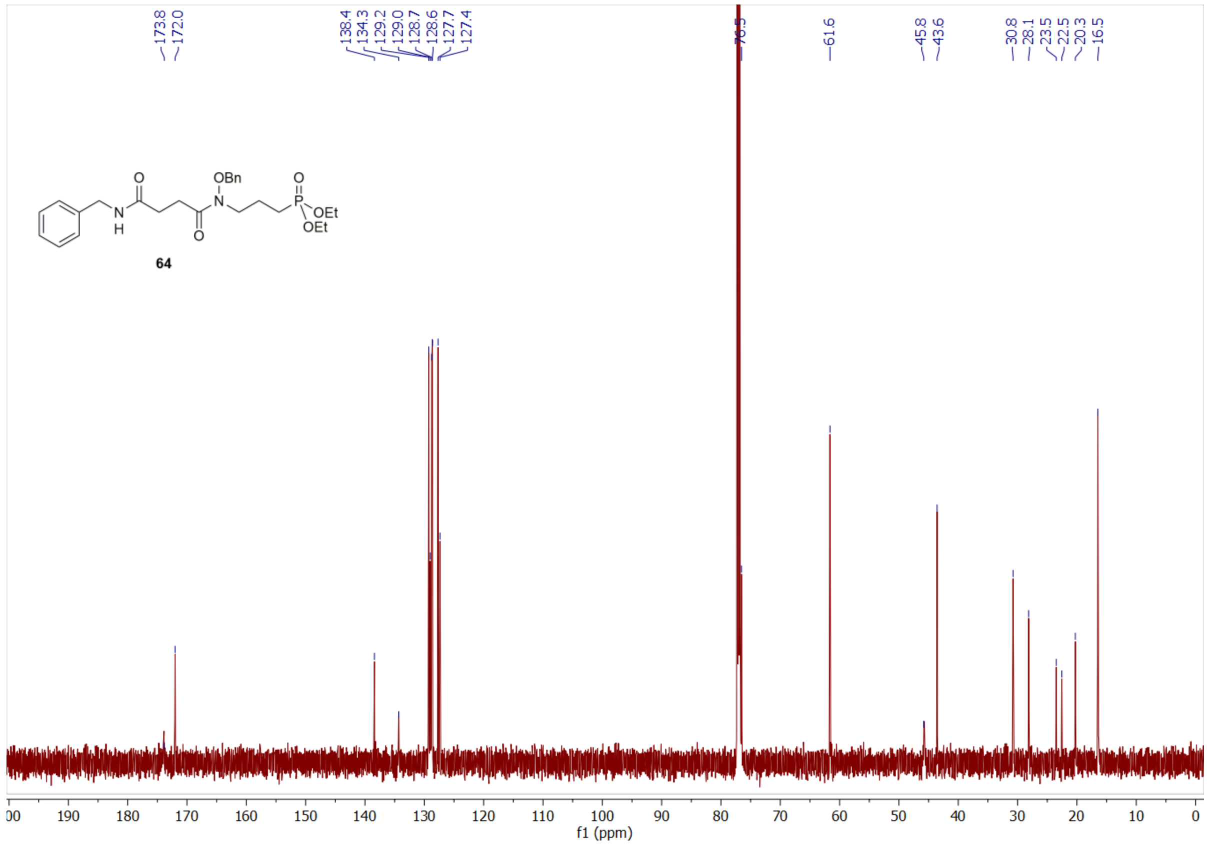

Figure S36. ^{13}**C-NMR spectrum of compound 64.**

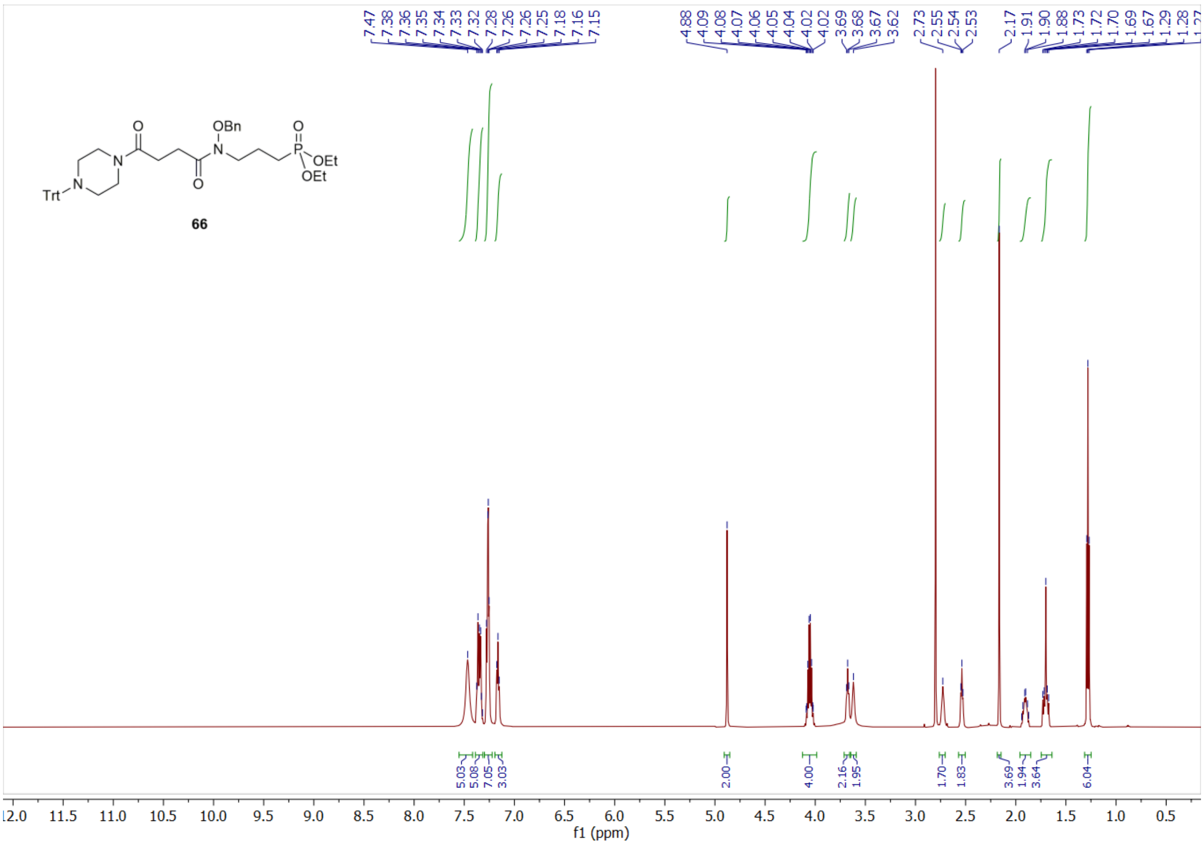

Figure S37. ^{1}H-NMR spectrum of compound 66.

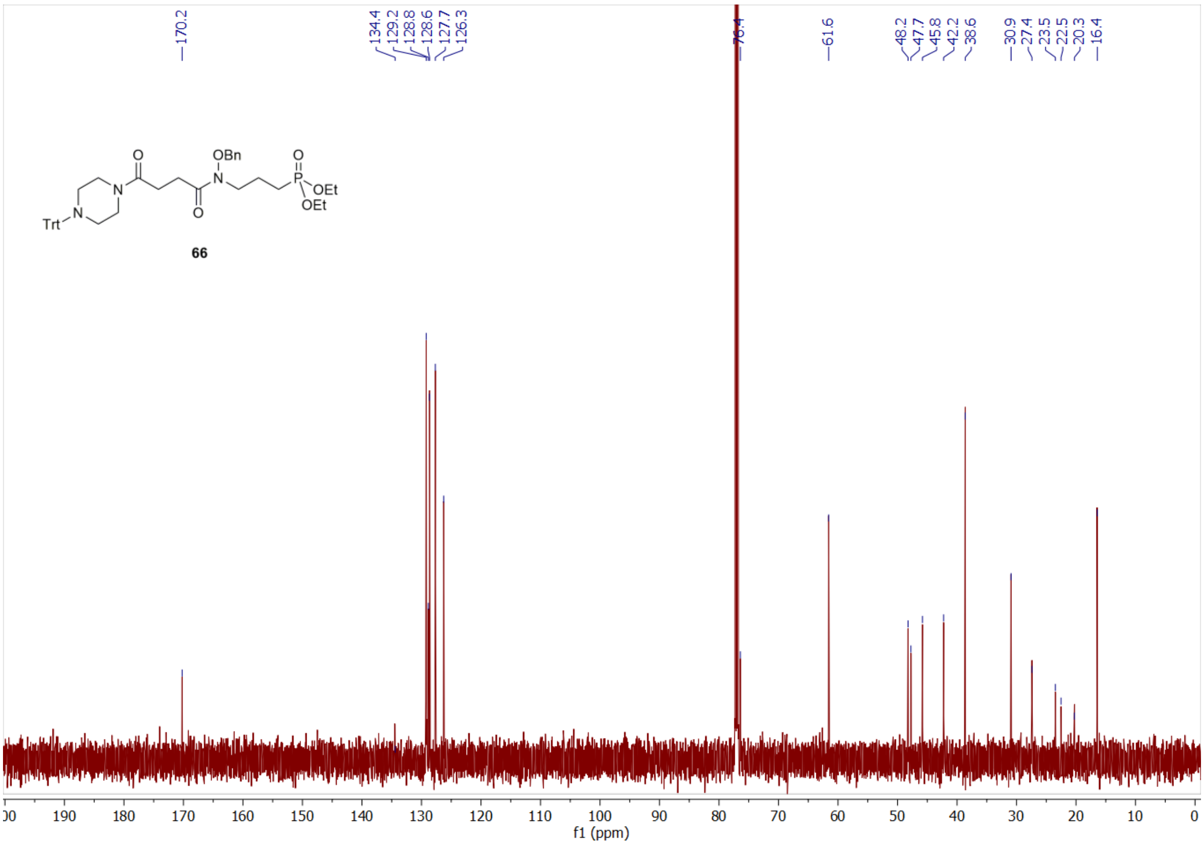

Figure S38. ^{13}C-NMR spectrum of compound 66.

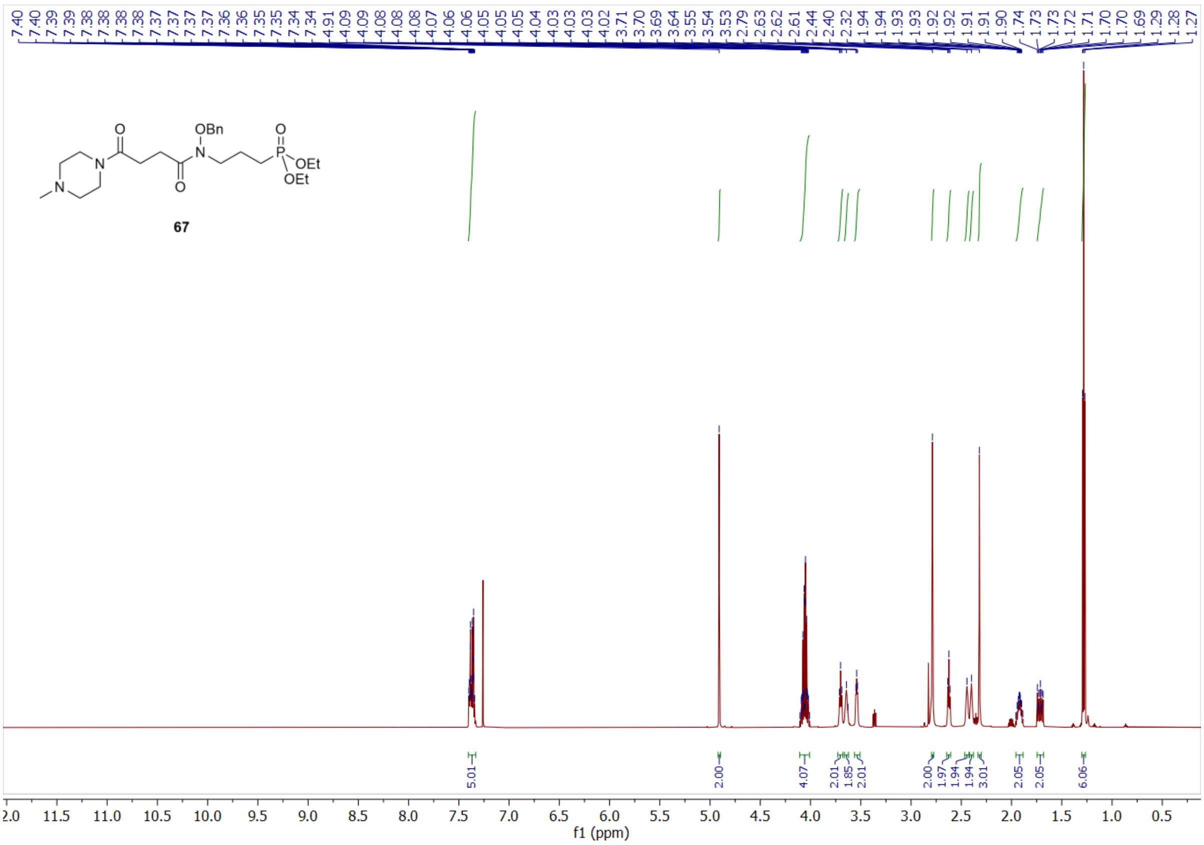

Figure S39. ^{1}**H-NMR spectrum of compound 67.**

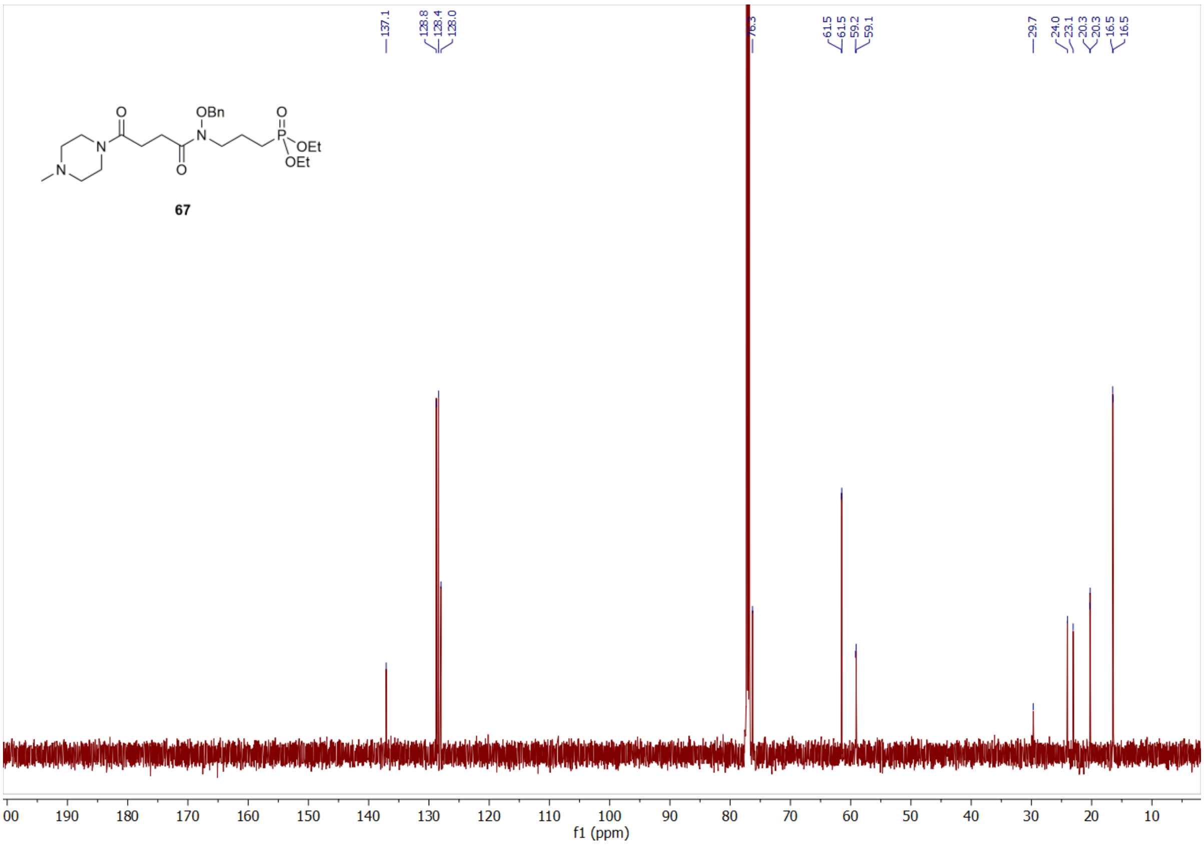

Figure S40. ^{13}**C-NMR spectrum of compound 67.**

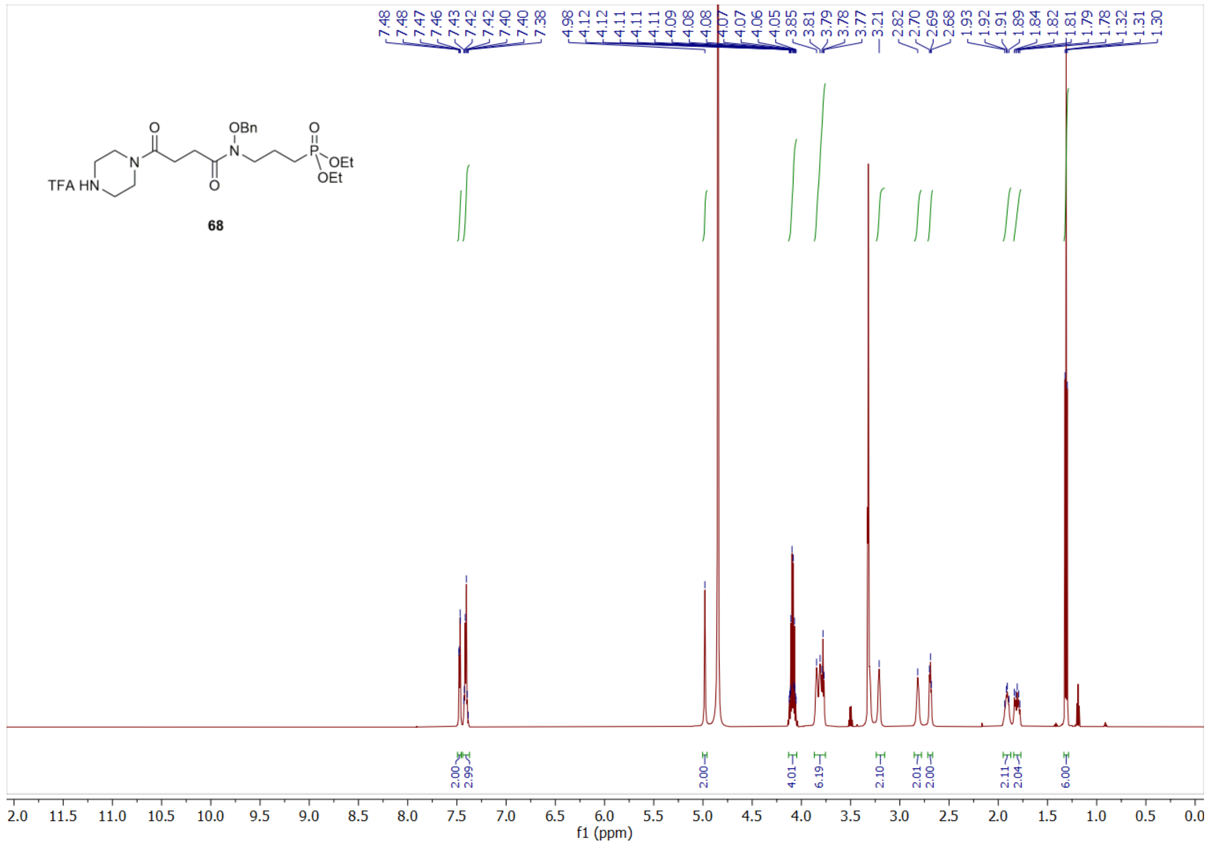

Figure S41. ^{1}H-NMR spectrum of compound 68.

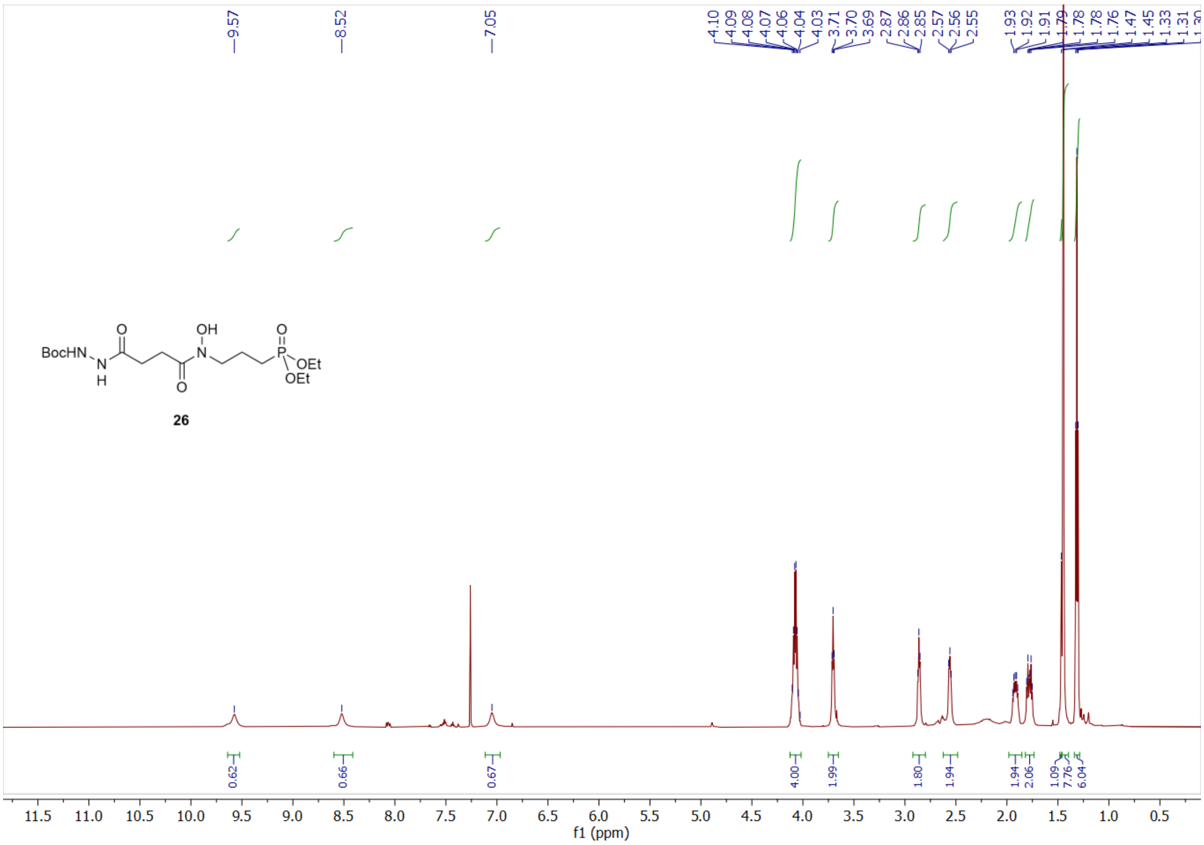

Figure S42. ¹H-NMR spectrum of compound 26.

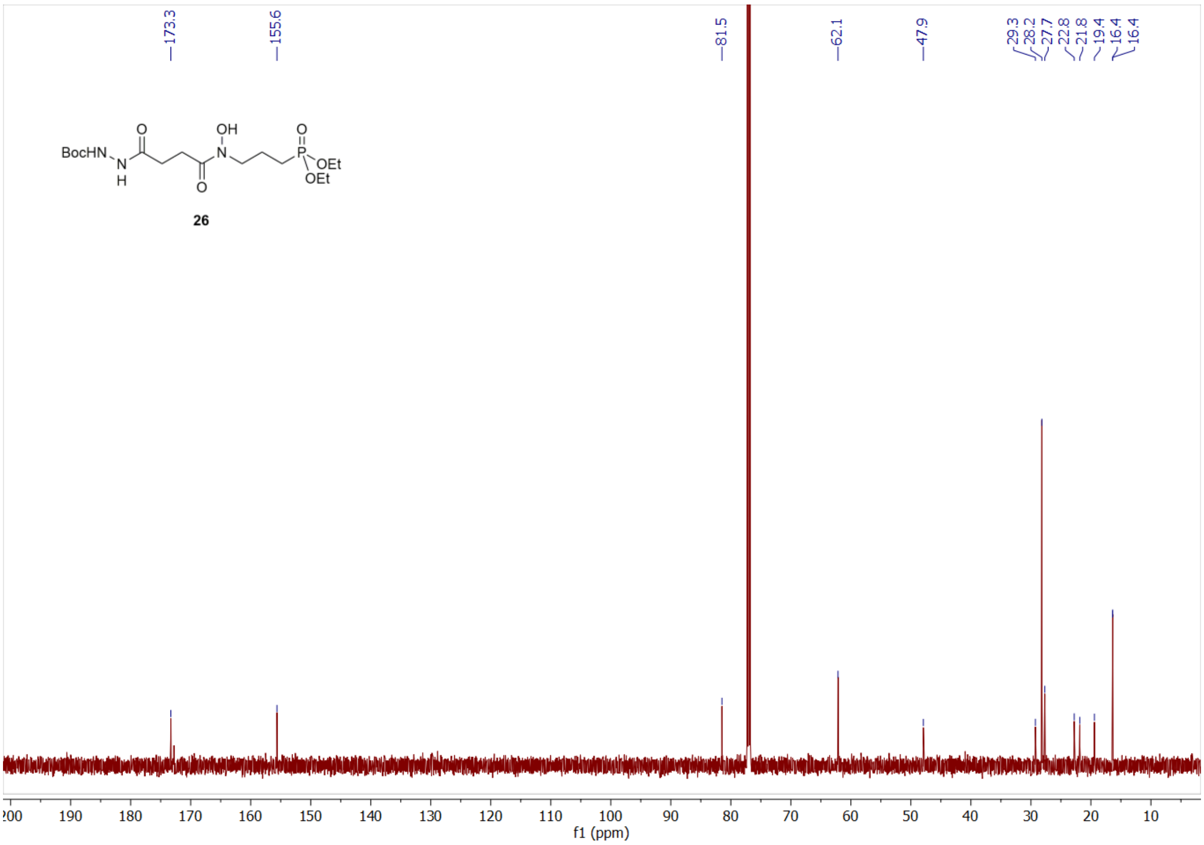

Figure S43. ¹³C-NMR spectrum of compound 26.

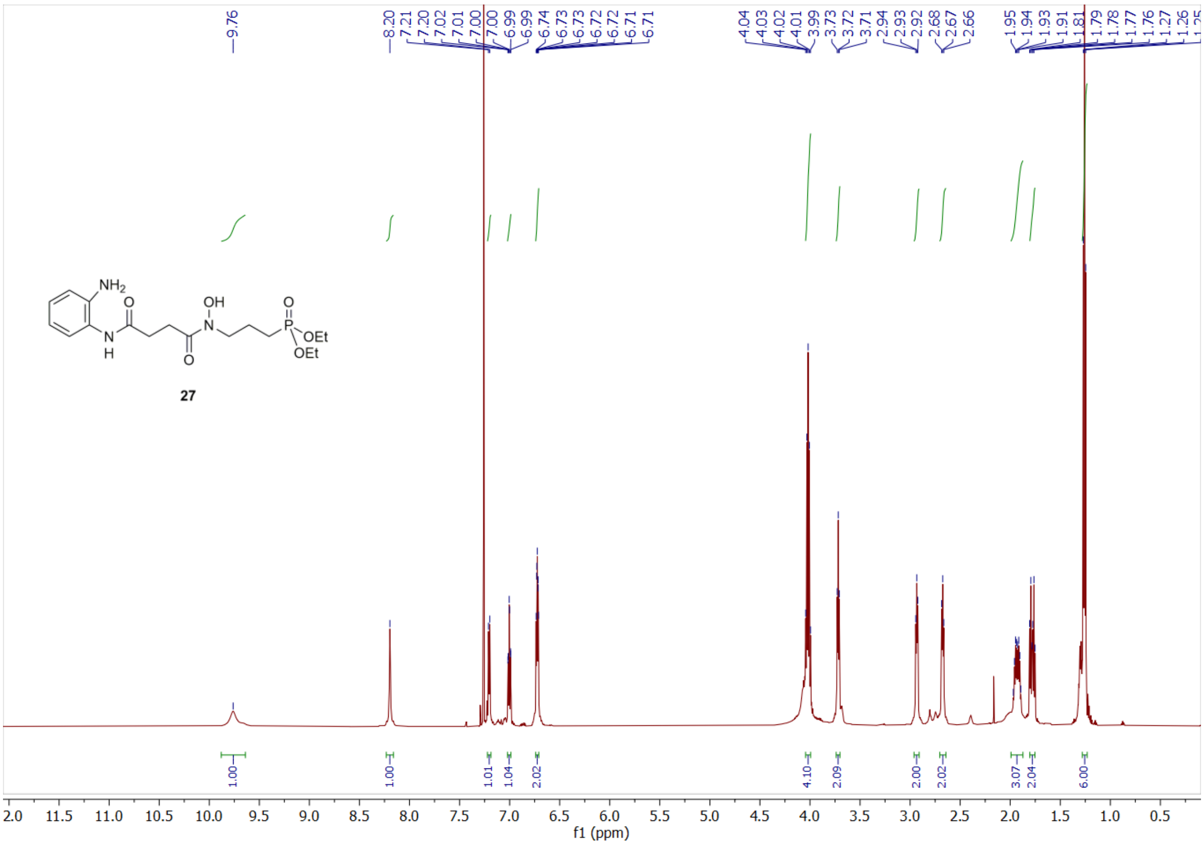

Figure S44. ^{1}H-NMR spectrum of compound 27.

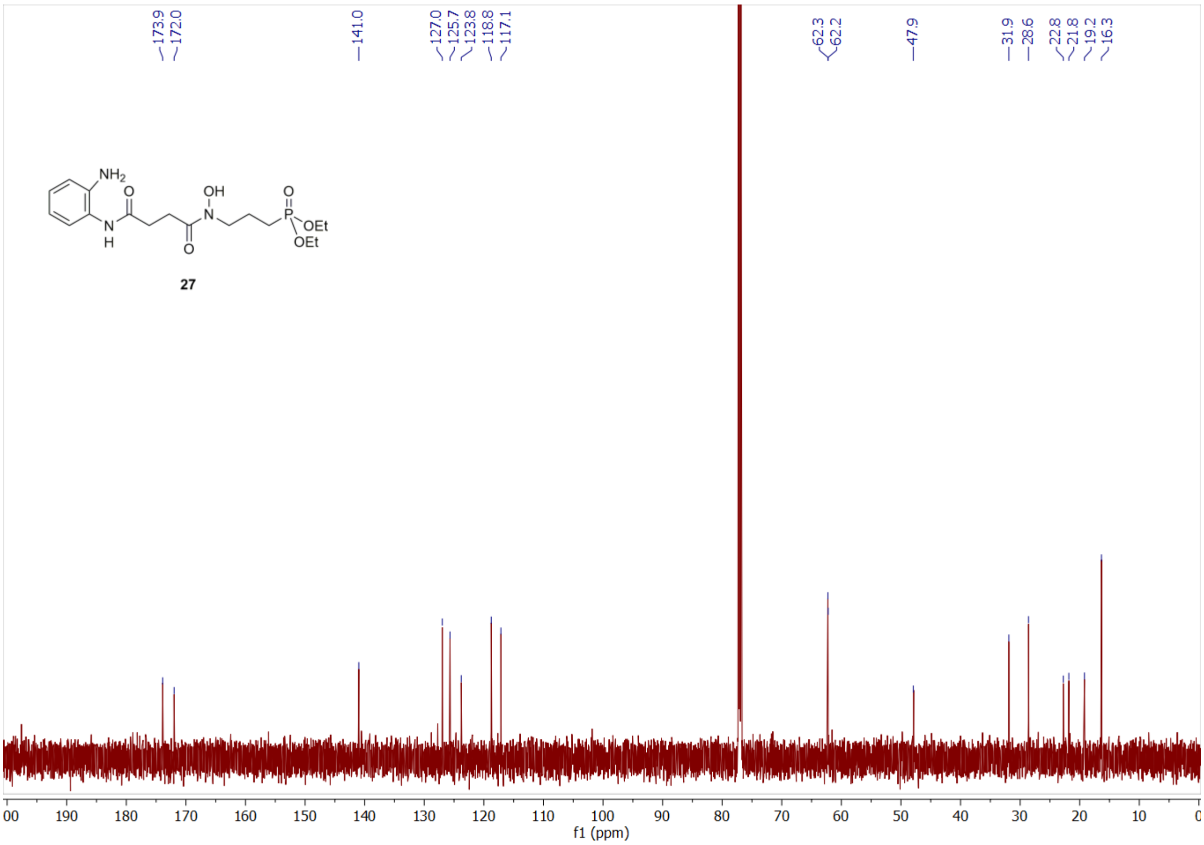

Figure S45. ^{13}C-NMR spectrum of compound 27.

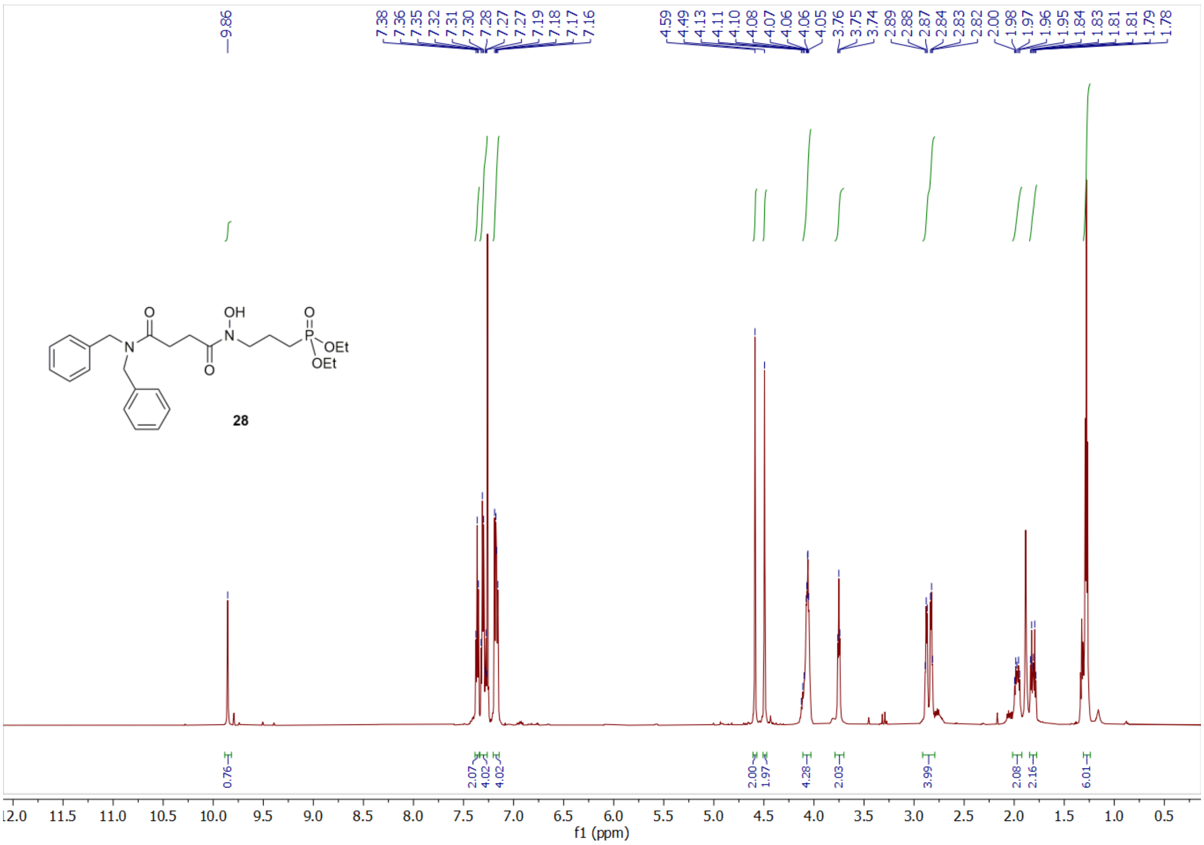

Figure S46. **^{1}H-NMR spectrum of compound 28.**

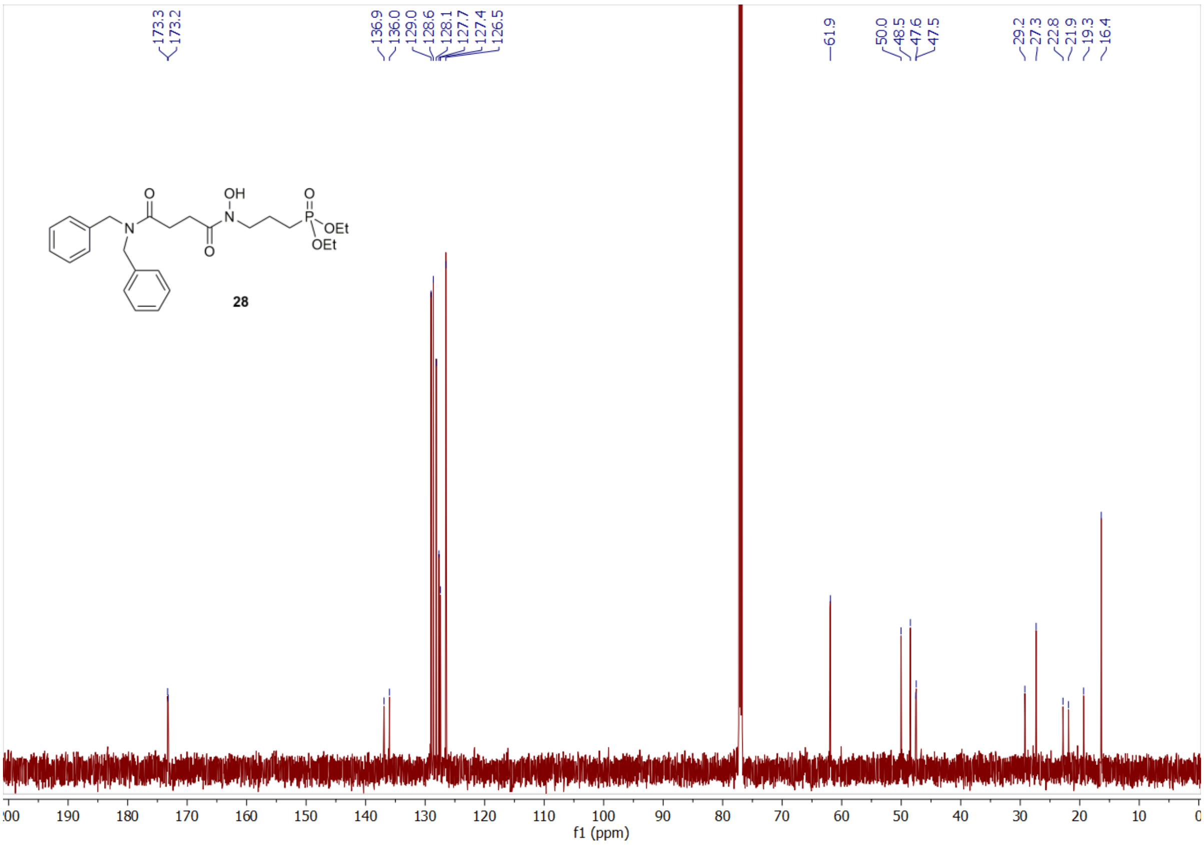

Figure S47. **^{13}C-NMR spectrum of compound 28.**

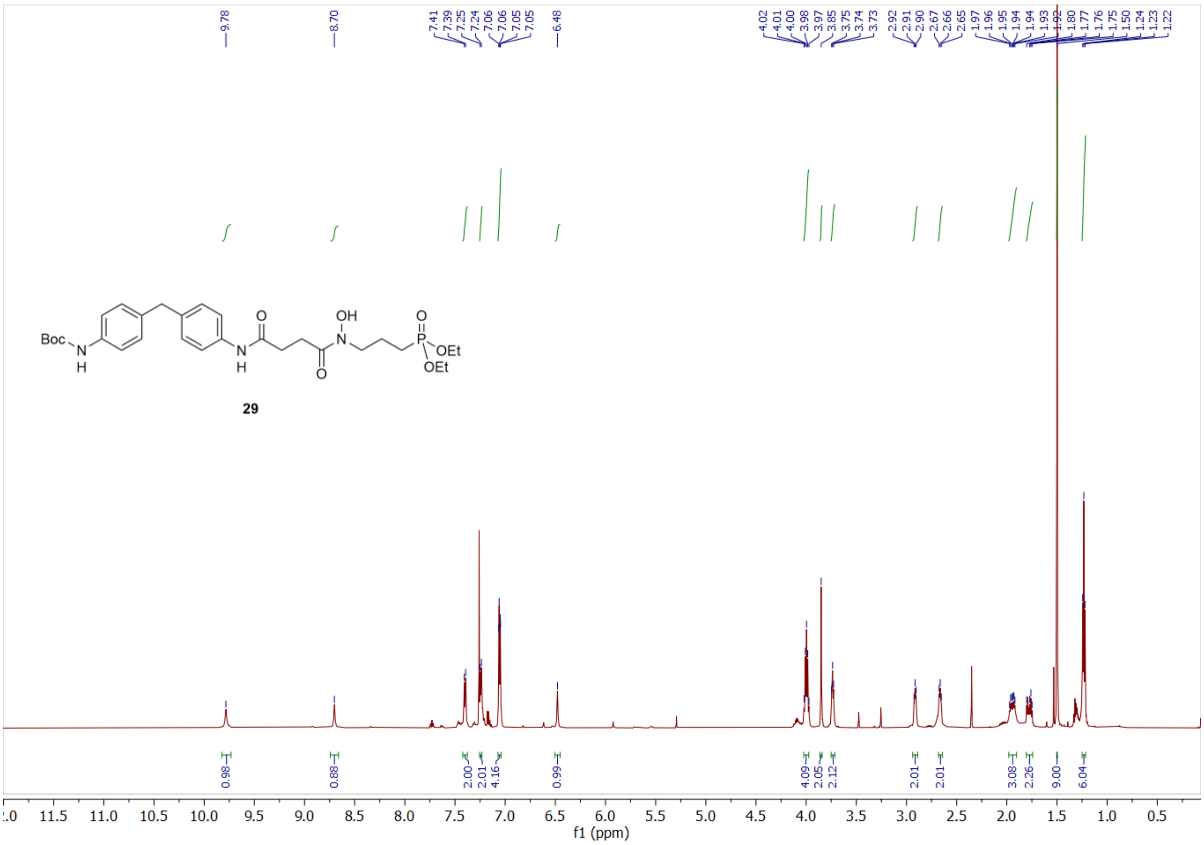

Figure S48. **^{1}H-NMR spectrum of compound 29.**

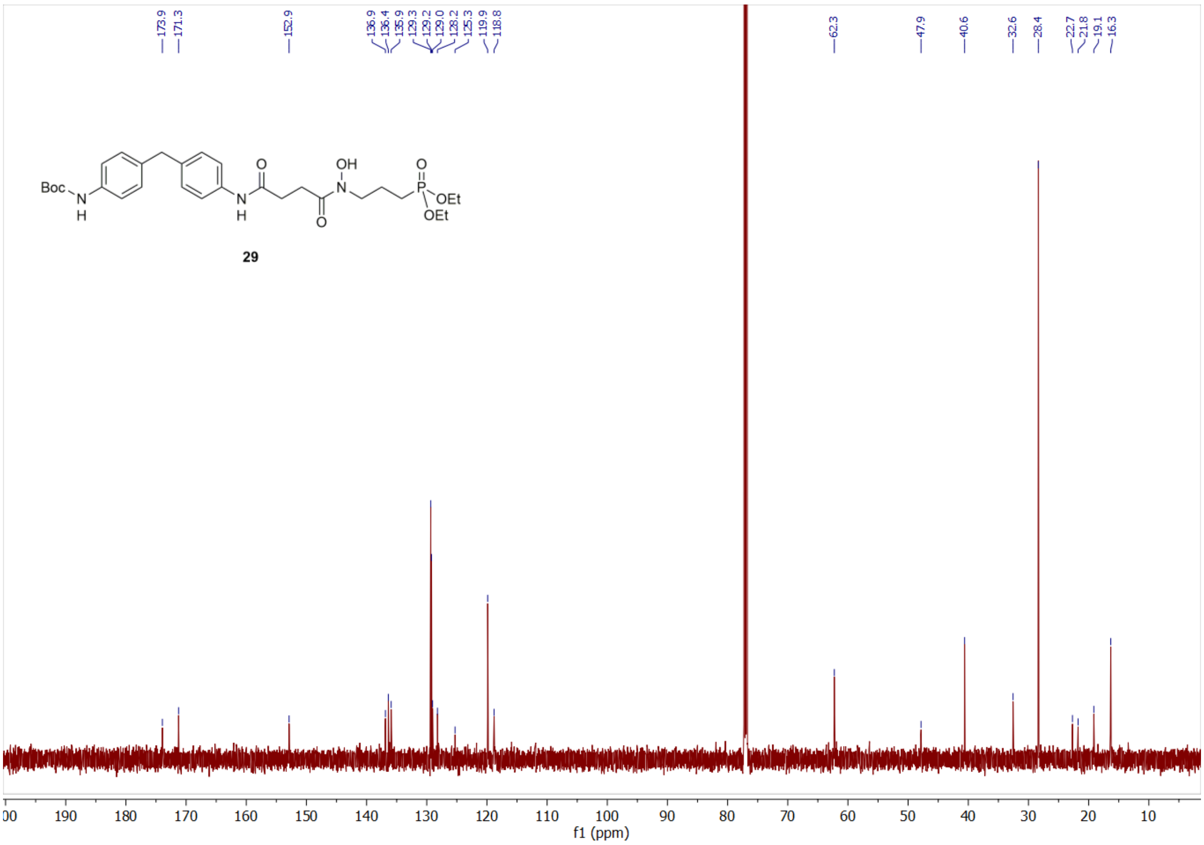

Figure S49. **^{13}C-NMR spectrum of compound 29.**

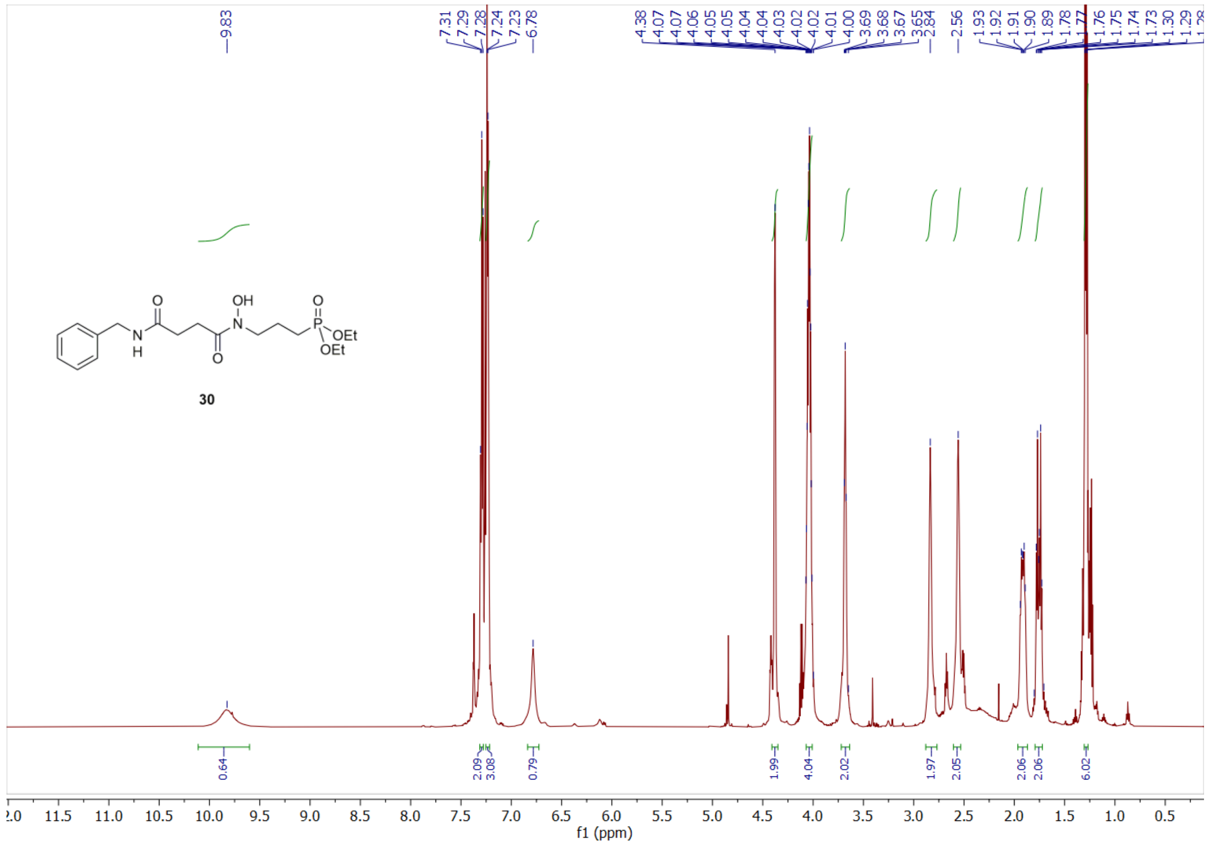

Figure S50. **^{1}H-NMR spectrum of compound 30.**

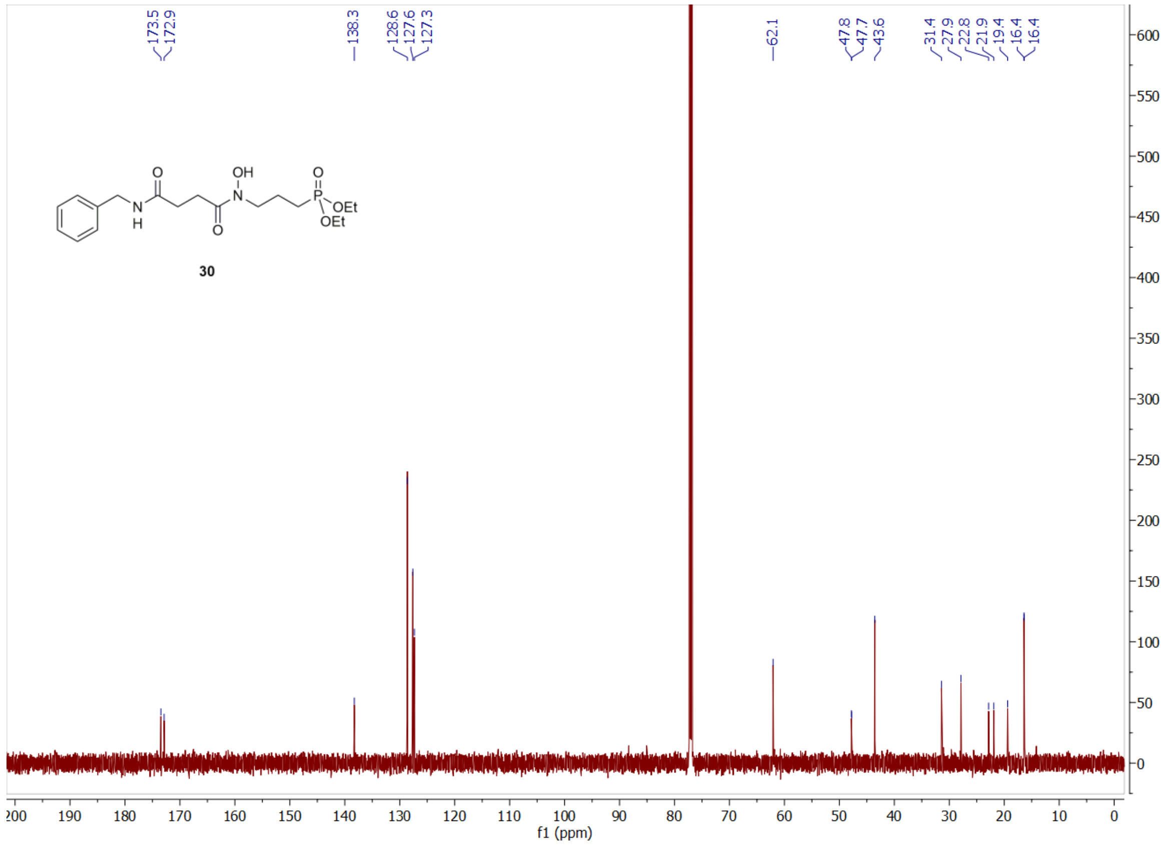

Figure S51. **^{13}C-NMR spectrum of compound 30.**

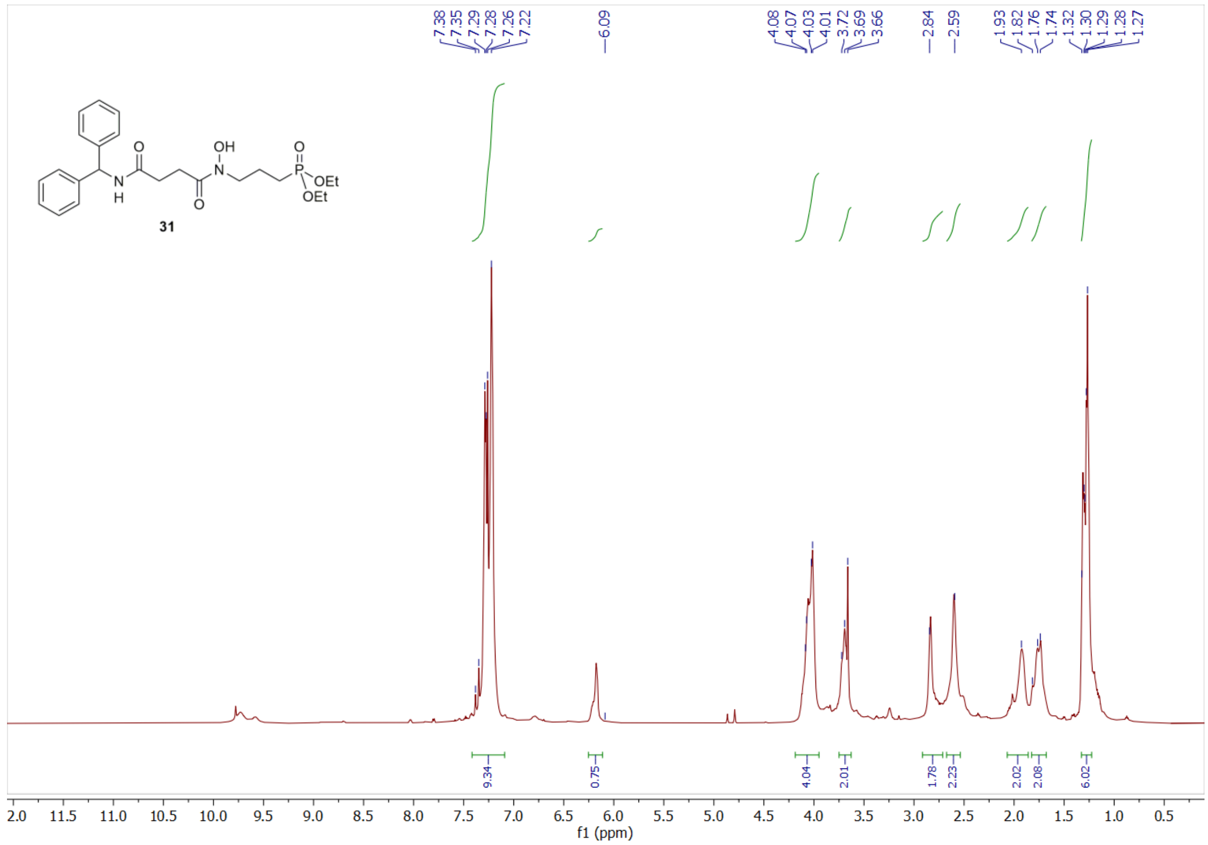

Figure S52. **^{1}H-NMR spectrum of compound 31.**

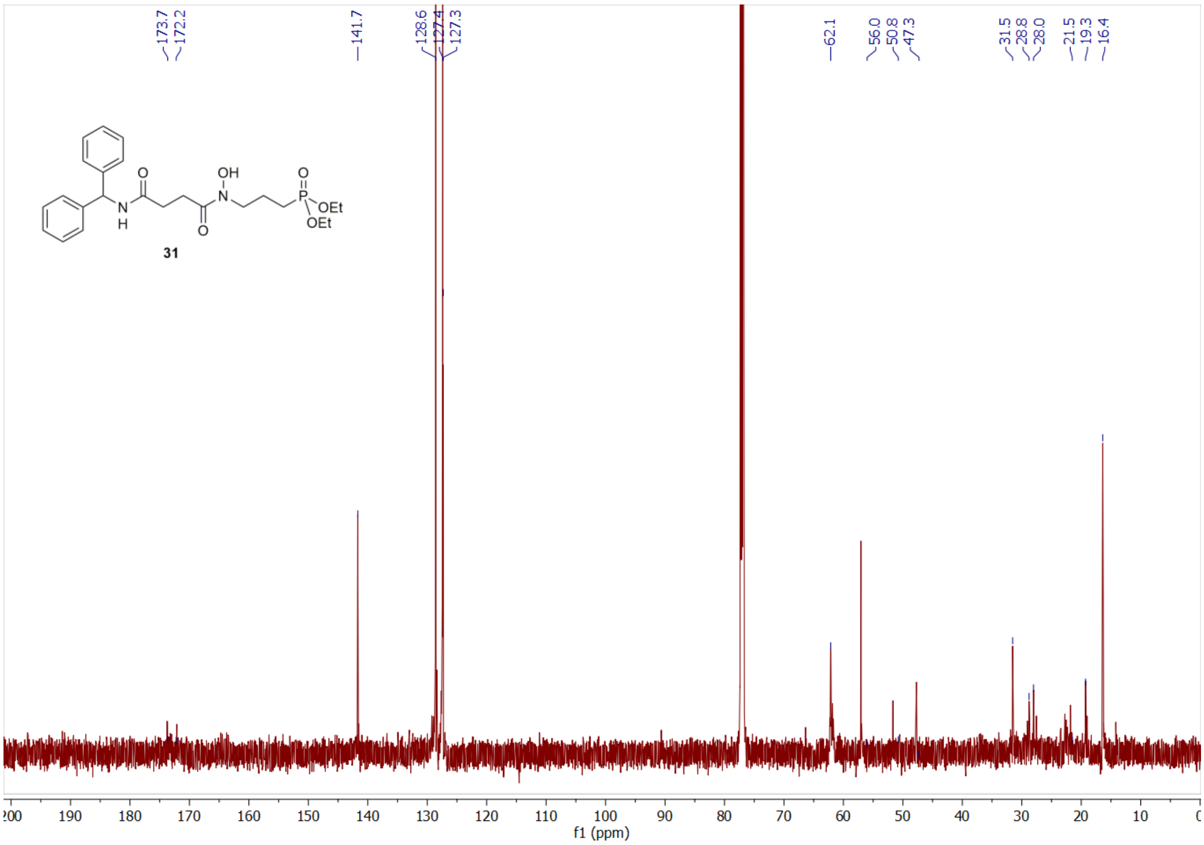

Figure S53. **^{13}C-NMR spectrum of compound 31.**

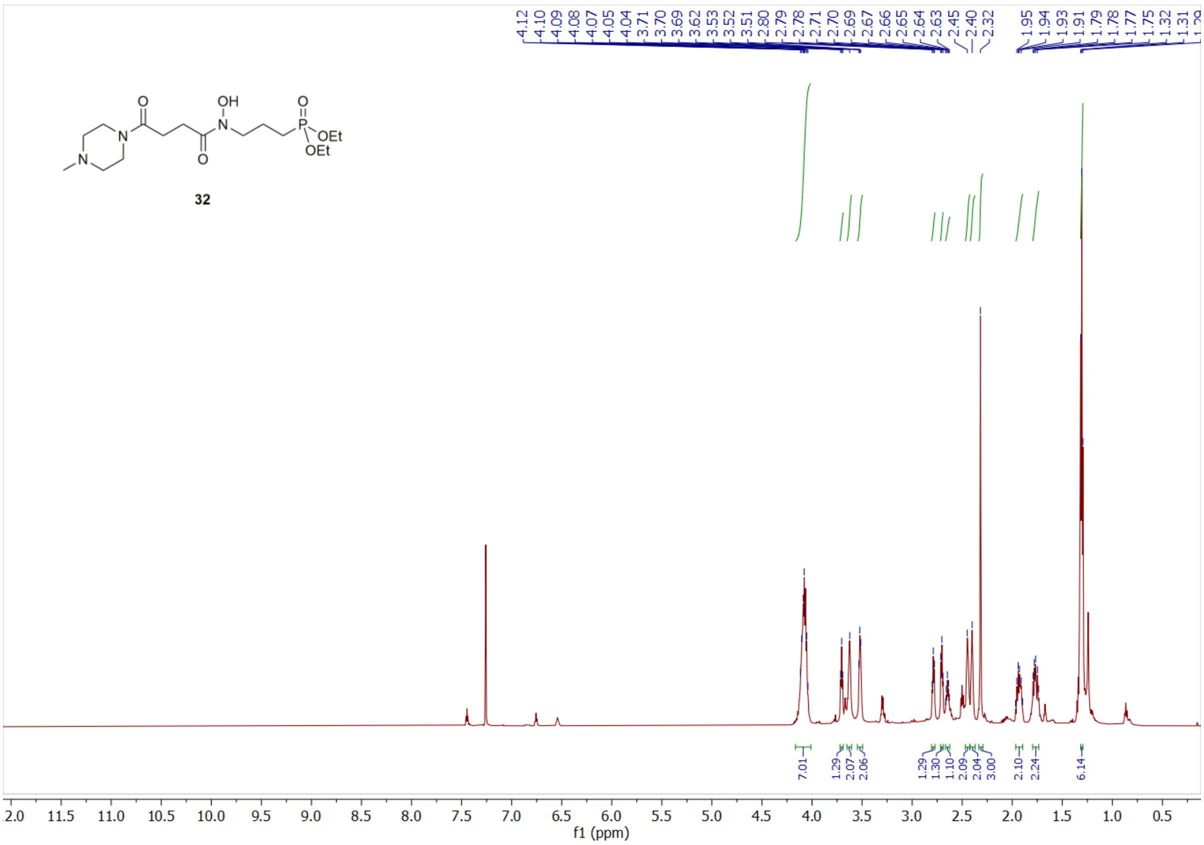

Figure S54. ^{13}**C-NMR spectrum of compound 32.**

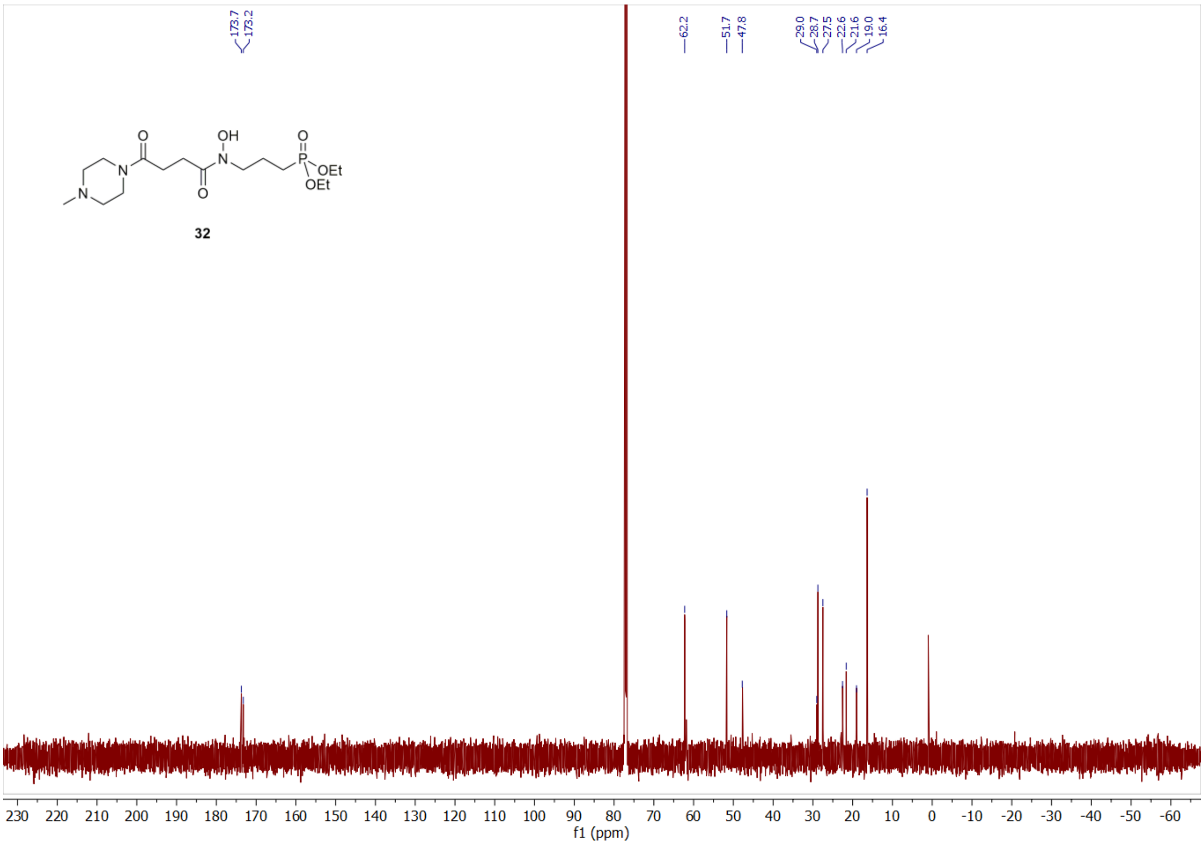

Figure S55. ^{13}**C-NMR spectrum of compound 32.**

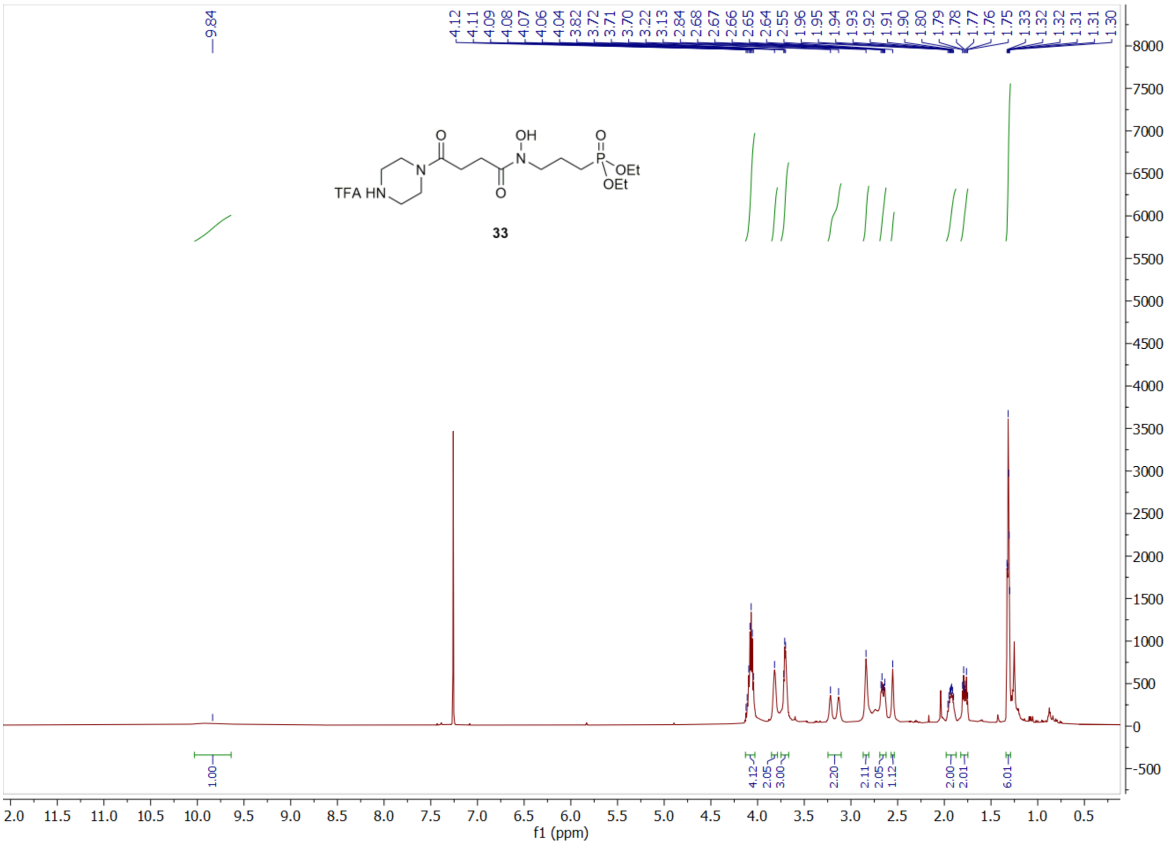

Figure S56. ¹H-NMR spectrum of compound 33.

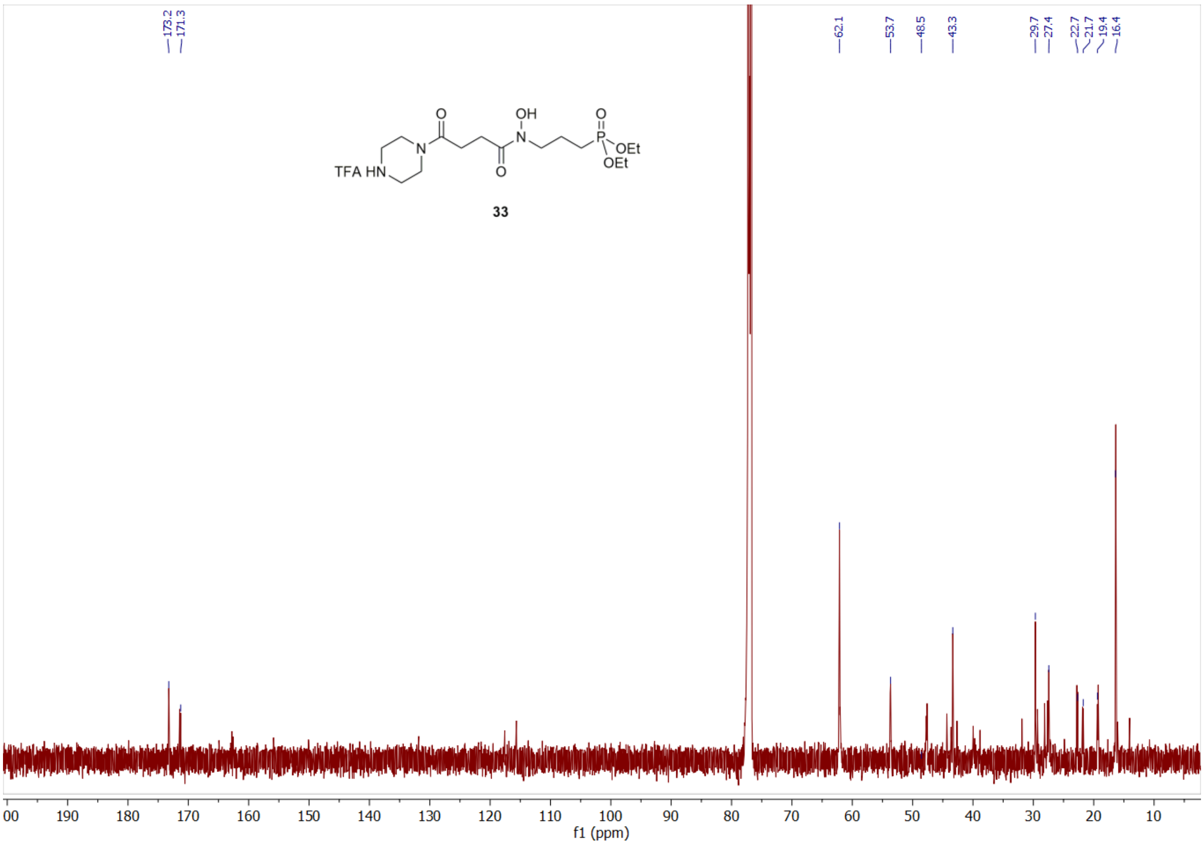

Figure S57. ¹H-NMR spectrum of compound 33.

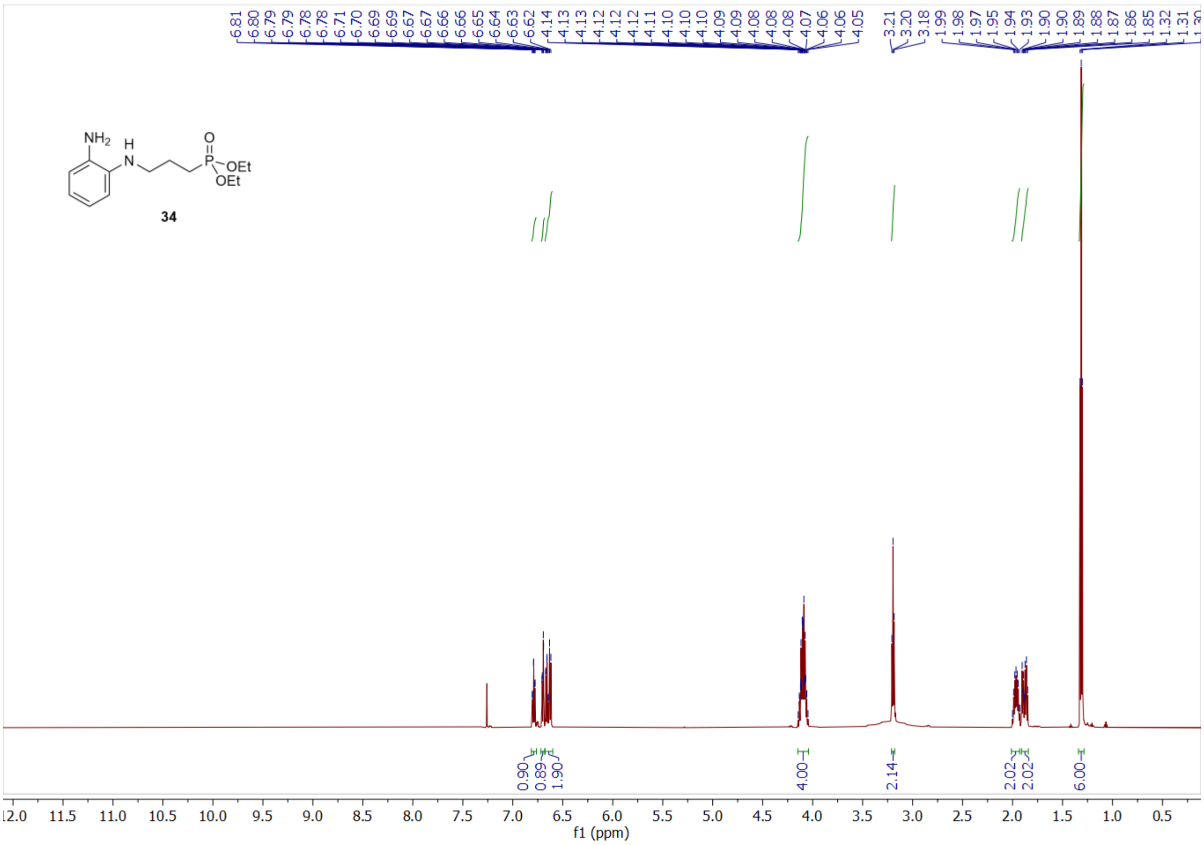

Figure S58. ^{1}H-NMR spectrum of compound 34.

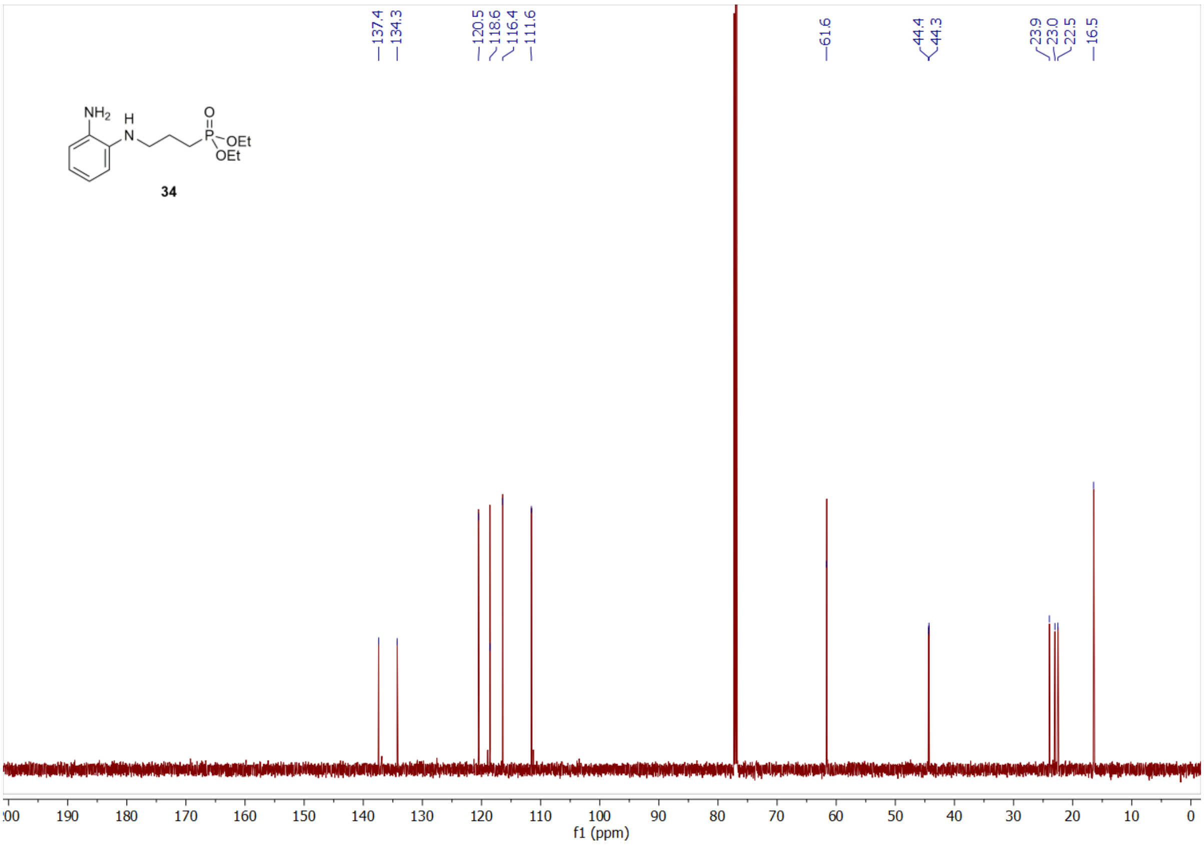

Figure S59. ^{13}C-NMR spectrum of compound 34.

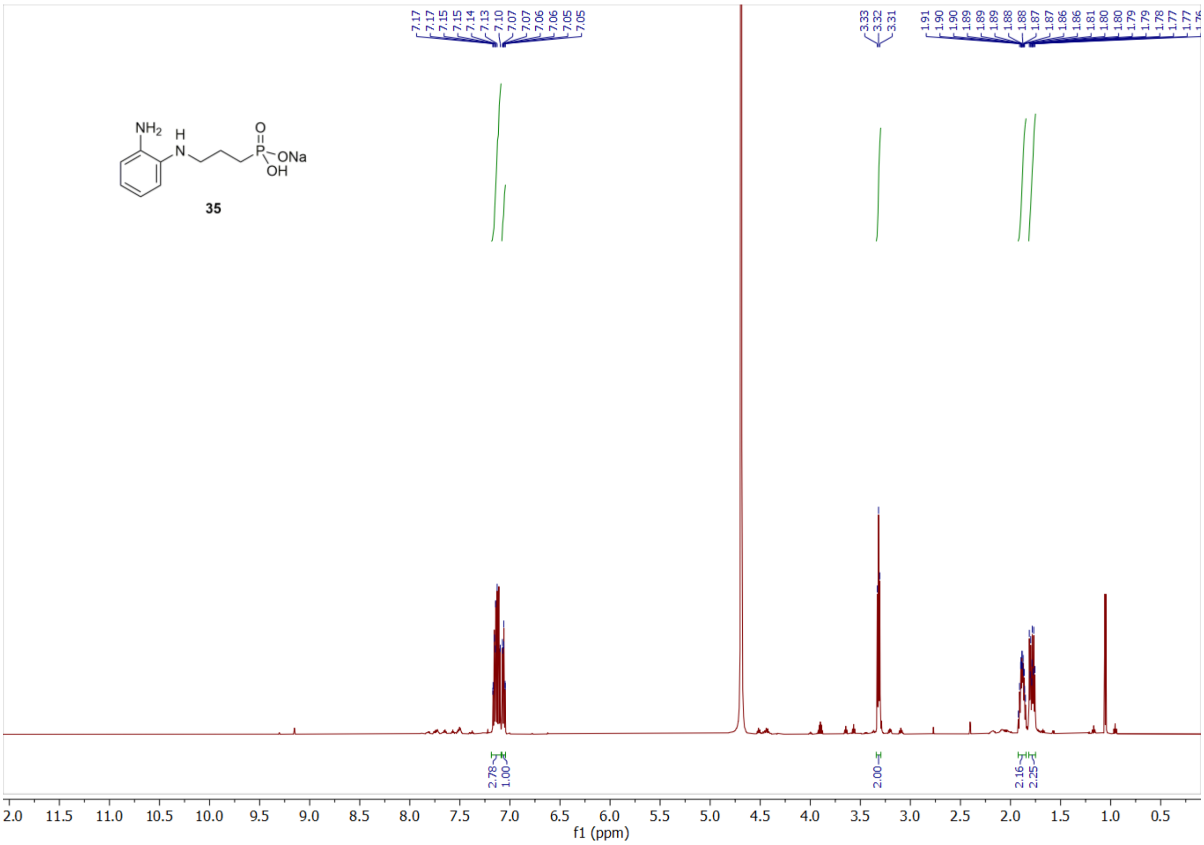

Figure S60. **^{1}H-NMR spectrum of compound 35.**

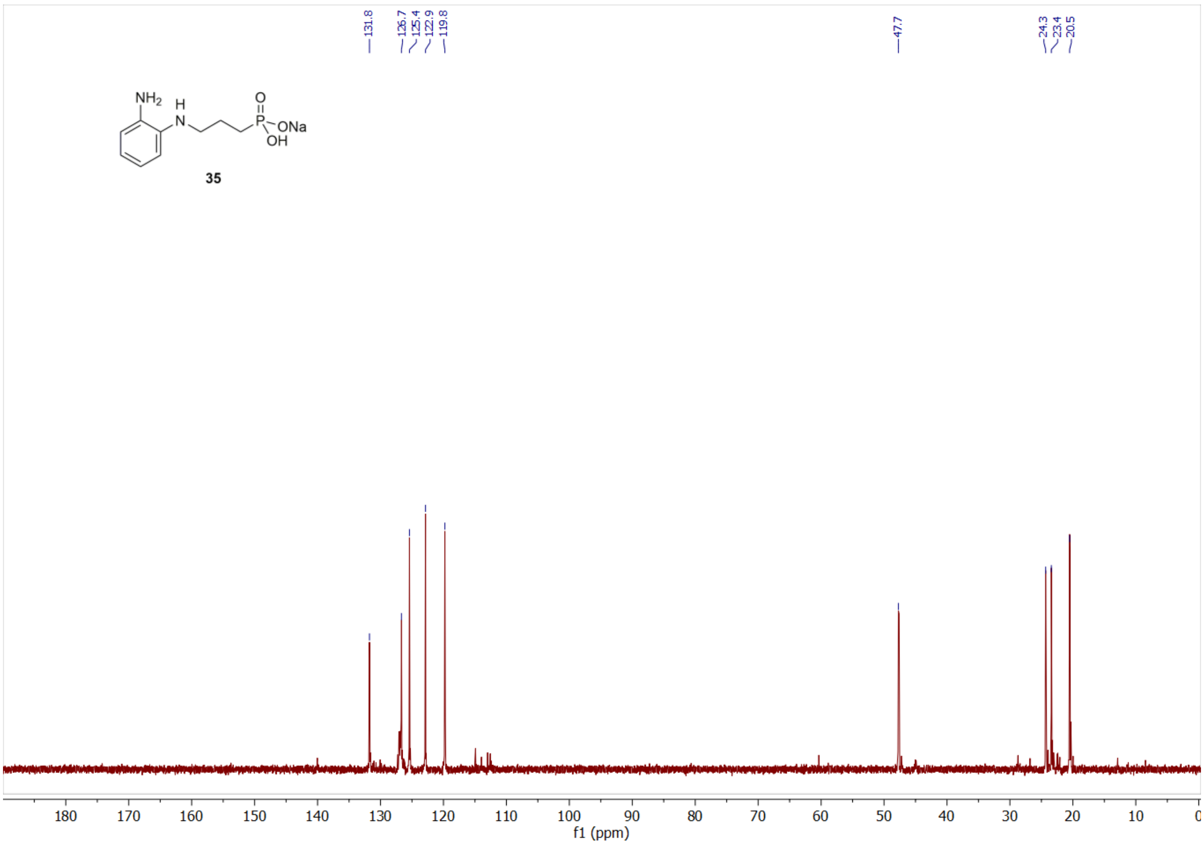

Figure S61. **^{13}C-NMR spectrum of compound 35.**
